# The genus *Macroteleia* Westwood (Hymenoptera, Platygastridae *s. l.*, Scelioninae) from China

**DOI:** 10.3897/zookeys.300.4934

**Published:** 2013-05-15

**Authors:** Hua-yan Chen, Norman F. Johnson, Lubomír Masner, Zai-fu Xu

**Affiliations:** 1 Department of Entomology, College of Natural Resources and Environment, South China Agricultural University, Guangzhou 510640, P. R. China; 2 Department of Evolution, Ecology and Organismal Biology, The Ohio State University, 1315 Kinnear Road, Columbus, Ohio 43212, U.S.A.; 3 Agriculture and Agri-Food Canada, K.W. Neatby Building, Ottawa, Ontario K1A 0C6, Canada

**Keywords:** Platygastridae *s. l.*, Scelioninae, egg parasitoid, key

## Abstract

The genus *Macroteleia* Westwood (Hymenoptera: Platygastridae *s. l.*, Scelioninae) from China is revised. Seventeen species are recognized based on 502 specimens, all of which are new records for China. Seven new species are described: *Macroteleia carinigena* **sp. n.** (China), *Macroteleia flava* **sp. n.** (China), *Macroteleia gracilis* **sp. n.** (China), *Macroteleia salebrosa* **sp. n.** (China), *Macroteleia semicircula* **sp. n.** (China), *Macroteleia spinitibia* **sp. n.** (China) and *Macroteleia striatipleuron* **sp. n.** (China). Ten species are redescribed: *Macroteleia boriviliensis* Saraswat (China, India, Thailand), *Macroteleia crawfordi* Kiefer, **stat. n.** (China, Philippines, Thailand, Vietnam), *Macroteleia dolichopa* Sharma (China, India, Vietnam), *Macroteleia emarginata* Dodd (China, Malaysia), *Macroteleia indica* Saraswat & Sharma (China, India, Vietnam), *Macroteleia lamba* Saraswat & Sharma (China, India, Thailand, Vietnam), *Macroteleia livingstoni* Saraswat (China, India), *Macroteleia peliades* Kozlov & Lê (China, Vietnam), *Macroteleia rufa* Szelényi (China, Egypt, Georgia, Russia, Thailand, Ukraine) and *Macroteleia striativentris* Crawford (China, Philippines, Thailand, Vietnam). The following five new synonyms are proposed: *Macroteleia crates* Kozlov & Lê **syn. n.** and *Macroteleia demades* Kozlov & Lê **syn. n.** of *Macroteleia crawfordi* Kieffer; *Macroteleia cebes* Kozlov & Lê **syn. n.** and *Macroteleia dones* Kozlov & Lê **syn. n.** of *Macroteleia indica* Saraswat & Sharma; *Macroteleia dores* Kozlov & Lê **syn. n.** of *Macroteleia lamba* Saraswat & Sharma. A key to the Chinese species of the genus is provided.

## Introduction

*Macroteleia* Westwood is a cosmopolitan genus in the subfamily Scelioninae, comprising about 131 described species worldwide (including 2 fossil species; Johnson 2012). Although *Macroteleia* are found on every continent except Antarctica, they are centred in the tropics and subtropics (Masner 1976). In the past century since Kieffer’s (1926) comprehensive study of this genus, several regional revisions have done for the New World (Muesebeck 1977), Australia (Galloway 1978), Vietnam (Lê 2000) and the Palearctic region (Kozlov and Kononova 1990; Kononova and Petrov 2003; Kononova and Kozlov 2008). In Asia, *Macroteleia* has been recorded from the Philippines, Borneo, Mongolia, India, Vietnam, Japan, Israel, Kazakhstan, Tajikistan and China (Dodd 1920; Kieffer 1926; Szabó 1973; Saraswat and Sharma 1978; Lê 2000; Kononova and Kozlov 2008; Johnson 2012). No species have been recorded formally from China.

Available host data suggest that species of *Macroteleia* are parasitoids of eggs of long-horned grasshoppers (Orthoptera: Tettigoniidae). In the original description of *Macroteleia virginiensis*, Ashmead (1893) mentioned that the specimens were reared from eggs of *Orchelimum glaberrimum* (Burmeister) (Orthoptera: Tettigoniidae), which was later considered to be *Orthoptera erythrocephalum* Davis (Muesebeck 1977). Morgan (1901) reported that the eggs of *Orthoptera agile* (De Geer) were parasitized by a *Macroteleia* species near *Macroteleia floridana* (Ashmead). In the original description of *Macroteleia surfacei*, Brues (1907) asserted that the specimens were reared during May from eggs of a “locustid”. Cole (1931) also listed *Macroteleia* species near *Macroteleia floridana* (Ashmead) as a parasite of *Conocephalus* sp. (Orthoptera: Tettigoniidae). Priesner (1951) supposed that *Macroteleia eremicola* Priesner, which was later considered to be a junior synonym of *Macroteleia rufa* Szelényi (Kononova and Kozlov 2008), parasitized eggs of Tettigoniidae based on the collecting habitat (halfa grass and sugar cane). In his revision of the New World *Macroteleia*, Muesebeck (1977) stated that *Macroteleia secreta* Muesebeck and *Macroteleia pilosa* Muesebeck were reared from eggs of Tettigoniidae, and some specimens of *Macroteleia punctulata* Kieffer and *Macroteleia macrogaster* Ashmead were reared from eggs of *Bucrates capitatus* (De Geer) (Orthoptera: Tettigoniidae) and *Orchelimum* sp., respectively.

During recent years, we have accumulated many specimens of Platygastridae *s. l.* during surveys of Chinese Hymenoptera. Among them, seventeen species of *Macroteleia* are recognized here, of which seven species are considered to be new to science and a total of 17 species are newly recorded from China. All the Chinese *Macroteleia* species are described and keyed and 6 new synonyms are proposed.

## Materials and methods

This work is based upon specimens deposited in the following collections, with abbreviations used in the text: IEBR, Institute of Ecology and Biological Resources, Hanoi, Vietnam; RABC, personal collection of R. A. Beaver, Chiang Mai, Thailand; SCAU, South China Agricultural University, Guangzhou, China; USNM, National Museum of Natural History, Washington, DC.

**Plate 1.**
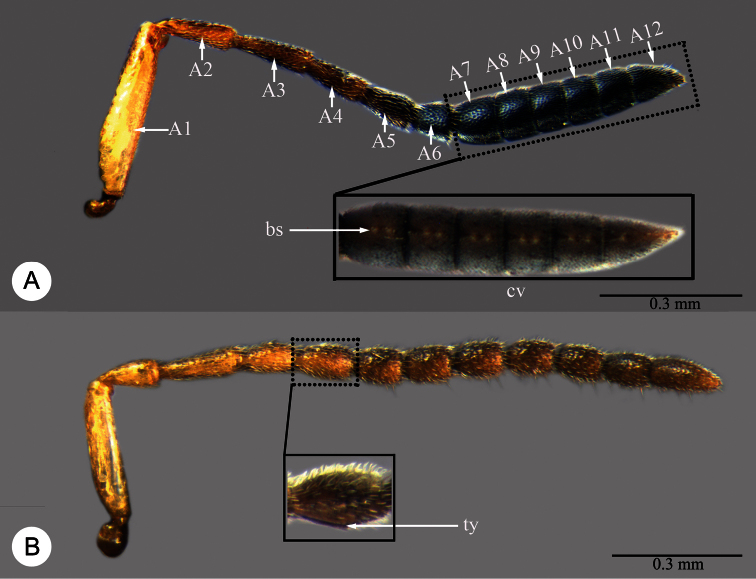
**A** *Macroteleia striatipleuron* sp. n., Antenna, female **B** *Macroteleia carinigena* sp. n., Antenna, male. A1, A2,... A12: antennomeres 1–12; bs: basiconic sensillum; cv: clava; ty: tyloid.

**Plate 2.**
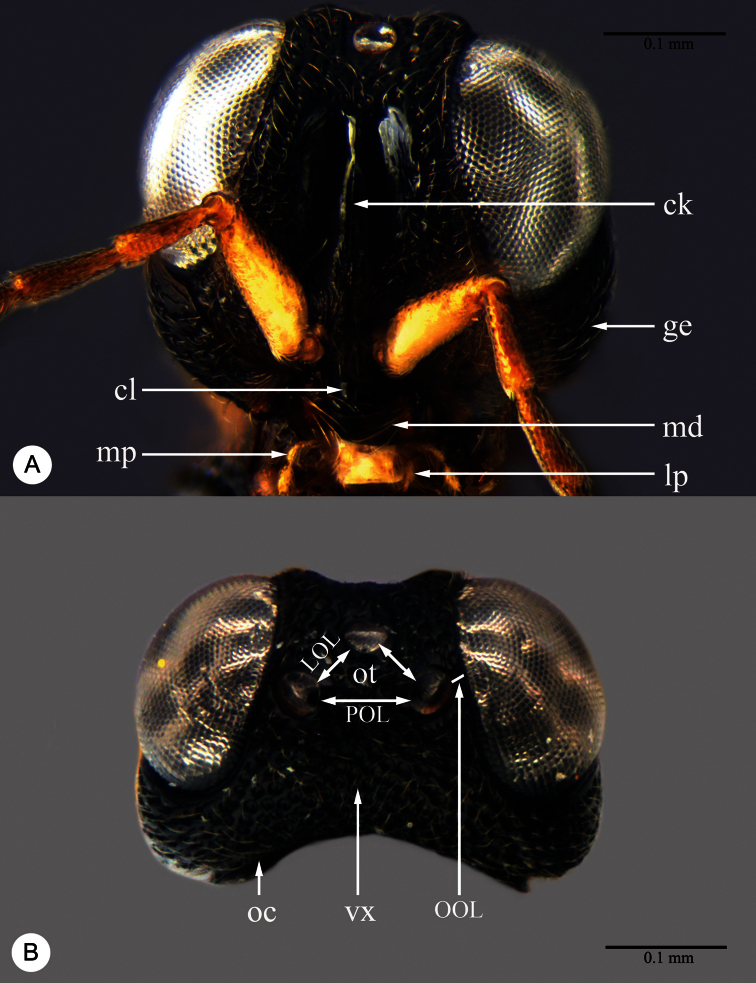
*Macroteleia striatipleuron* sp. n., female. **A** Head, anterior view **B** Head, dorsal view. ck: central keel; cl: clypeus; ge: gena; LOL: lateral ocellar line; lp: labial palpus; md: mandible; mp: maxillary palpus; oc: occipital carina; OOL: ocular ocellar line; ot: ocellar triangle; POL: posterior ocellar line; vx: vertex.

**Plate 3.**
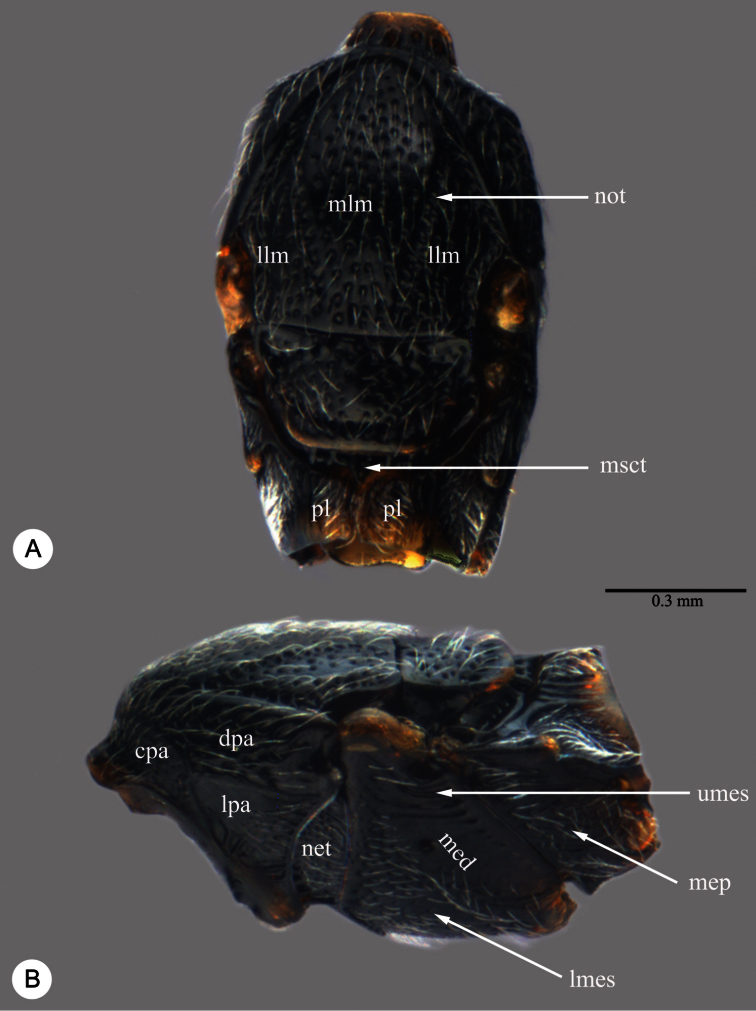
*Macroteleia striativentris* Crawford, female. **A** Mesosoma, dorsal view **B** Mesosoma, lateral view. cpa: cervical pronotal area; dpa: dorsal pronotal area; lpa: lateral pronotal area; llm: lateral lobe of mesoscutum; lmes: lower mesepisternum; med: mesopleural depression; mep: metapleuron; mlm: middle lobe of mesoscutum; msct: metascutellum; net: netrion; not: notauli; pl: propodeal lobe; umes: upper mesepisternum.

Abbreviations and morphological terms used in text: A1, A2,... A12 (Plate 1): antennomere 1, 2, … 12; LOL (Plate 2B): lateral ocellar line, shortest distance between inner margins of median and lateral ocelli (Masner 1980); OOL (Plate 2B): ocular ocellar line, shortest distance from inner orbit and outer margin of posterior ocellus (Masner 1980); POL (Plate 2B): posterior ocellar line, shortest distance between inner margins of posterior ocelli (Masner 1980); T1, T2,... T7: metasomal tergite 1, 2,... 7 (Plates 5, 7); S1, S2, … S7: metasomal sternite 1, 2, … 7 (Plates 5, 7). Morphological terminology otherwise generally follows Masner (1980) and Mikó et al. (2007); the following are illustrated and labeled to aid in the description of *Macroteleia*. Central keel (ck: Plate 2A); Clypeus (cl: Plate 2A); Gena (ge: Plate 2A); Cervical pronotal area (cpa: Plate 3B); Dorsal pronotal area (dpa: Plate 3B); Lateral lobe of mesoscutum (llm: Plate 3A); Longitudinal median sternal carina (lmc: Plate 5C); Lower mesepisternum (lmes: Plate 3B); Labial palpus (lp: Plate 2A); Lateral pronotal area (lpa: Plate 3B); Mandible (md: Plate 2A); Mesopleural depression (med: Plate 3B); Metapleuron (mep: Plate 3B); Middle lobe of mesoscutum (mlm: Plate 3A); Maxillary palpus (mp: Plate 2A); Metascutellum (msct: Plate 3A); Netrion (net: Plate 3B); Notauli (not: Plate 3A); Occipital carina (oc: Plate 2B); Ocellar triangle (ot: Plate 2B); Propodeal lobe (pl: Plate 3A); Sublateral tergal carina (stc: Plate 5A); Transverse sulcus of T2 (trs: Plate 5A); Upper mesepisternum (umes: Plate 3B); Vertex (vx: Plate 2B). Appendix I lists terms associated with identifiers in the Hymenoptera Anatomy Ontology (Yoder et al. 2010).

Descriptions and measurements were made under a stereomicroscope (Olympus SZ61). Images were processed with a Photometrics CoolSNAP Camera attached to a stereomicroscope (Zeiss Stemi 2000-CS) and Image Pro Plus version 6.0 software and figures were post-processed with Adobe Photoshop CS3 Extended.

## Taxonomy

### Macroteleia

Westwood

http://species-id.net/wiki/Macroteleia

Macroteleia Westwood 1835: 70. Original description. Type: *Macroteleia cleonymoides* Westwood, by monotypy. Brullé 1846: 621 (description); Ashmead 1893: 209, 210, 211, 216 (description, keyed, key to species of U.S. and Canada); Ashmead 1894: 216, 222 (key to species of St. Vincent, keyed); Dalla Torre 1898: 501 (catalog of species); Ashmead 1900: 327 (list of species of West Indies); Ashmead 1903: 91, 92, 93, 94 (keyed); Brues 1907: 154 (key to species of United States); Brues 1908: 27, 28, 34, 51 (diagnosis, list of species, keyed); Kieffer 1908a: 22 (key to new species described); Kieffer 1908b: 122, 170 (key to species, keyed); Kieffer 1910a: 316 (key to species of Brazil); Kieffer 1910b: 66, 89 (description, list of species, keyed); Kieffer 1912: 59 (key to species of Seychelles); Dodd 1913a: 131, 151 (key to species of Australia, keyed); Dodd 1913b: 176 (comparison with *Ceratoteleia* Kieffer); Kieffer 1913a: 232 (description); Kieffer 1913b: 323 (key to species of the Philippines); Kieffer 1914a: 312 (description, key to species of Europe and Algeria); Kieffer 1914b: 298 (key to species of the Philippines); Dodd 1915: 12 (key to species of Australia); Kieffer 1917: 55 (key to species of the Philippines); Kieffer 1926: 273, 520 (description, keyed, key to species); Nixon 1931: 367 (keyed, key to species of Africa); Dodd 1933: 75 (comparison with *Prosapegus* Kieffer key to species of Australia); Nixon 1933: 292 (keyed); Maneval 1940: 114 (keyed); Risbec 1950: 598 (key to species of Africa); Muesebeck and Walkley 1951: 706 (catalog of species of U.S. and Canada); Muesebeck and Walkley 1956: 367 (citation of type species); Masner 1964: 137 (description); Baltazar 1966: 183 (catalog of species of the Philippines); Szabó 1966: 421 (key to species of Hungary known to the author); De Santis 1967: 226 (catalog of species of Argentina); Muesebeck and Walkley 1967: 299 (second supplement to Muesebeck and Walkley (1951)); Kozlov 1971: 40 (keyed); Masner 1976: 27 (key to *Macroteleia* Westwood and *Triteleia* Kieffer); Muesebeck 1977: 1 (description, key to species of the New World); Galloway 1978: 298 (description, key to species of Australia); Kozlov 1978: 614 (key to species of the European USSR); Muesebeck 1979: 1153 (catalog of species of U.S. and Canada); De Santis 1980: 313 (catalog of species of Brazil); Mani and Sharma 1982: 168 (description, key to species of India); Saraswat 1982: 343 (key to species of India); Galloway and Austin 1984: 9, 14 (list of species described from Australia, keyed); Kozlov and Kononova 1987: 93 (key to species of Palearctic region); Kozlov and Kononova 1990: 95, 173, 188, 189 (description, key to species of Palearctic, keyed); Carpenter 1992: 471 (fossil references); Johnson 1992: 423 (catalog of world species); Kononova 1995: 61, 69 (keyed, diagnosis, key to species of Russian Far East); Austin and Field 1997: 22, 689 (structure of ovipositor system, discussion of phylogenetic relationships); Lê 2000: 31, 52 (keyed, description, key to species); Loiácono and Margaría 2002: 557 (catalog of Brazilian species); Kononova and Petrov 2003: 604, 605 (description, key to species of Palearctic region); Rajmohana K 2006: 117, 125 (description, keyed); Kononova and Kozlov 2008: 22, 230, 231 (description, keyed, key to species of Palearctic region); Popovici and Johnson 2012: 380 (description of internal genitalia).
Baeoneura Förster 1856: 100, 102. Original description. Type: *Baeoneura floridana* Ashmead, designated by Muesebeck and Walkley (1956). Howard 1886: 172 (keyed); Cresson 1887: 83, 313 (keyed, catalog of species of U.S. and Canada); Provancher 1888: 402 (description); Ashmead 1893: 210, 211, 234 (description, keyed); Ashmead 1894: 217 (keyed); Dalla Torre 1898: 498 (catalog of species); Ashmead 1903: 92, 94 (keyed); Brues 1908: 27, 28, 39 (diagnosis, list of species, keyed); Kieffer 1908b: 115 (keyed); Kieffer 1910b: 63, 72 (description, list of species, keyed); Kieffer 1913a: 223, 235 (description); Kieffer 1926: 267, 351 (description, keyed); Maneval 1940: 112 (keyed); Muesebeck and Walkley 1956: 335 (junior synonym of *Macroteleia* Westwood).
Macrotelia Förster 1856: 105 (diagnosis, spelling error).
Parapegus Kieffer 1908b: 149. Original description. Type: *Apegus (Parapegus) punctatus* Kieffer, designated by Kieffer (1910b). Kieffer 1913a: 232 (description); Kieffer 1914a: 306 (description, key to species of Europe and Algeria); Kieffer 1926: 273, 497 (description, keyed, key to species); Maneval 1940: 114 (keyed); Masner 1956: 235 (diagnosis, key to species); Muesebeck and Walkley 1956: 380 (citation of type species); Kozlov 1971: 40 (keyed); Masner 1976: 27 (junior synonym of *Macroteleia* Westwood); Kozlov 1978: 616 (key to species of the European USSR).
Prosapegus Kieffer 1908b: 121, 147. Original description. Type: *Anteris elongata* Ashmead, by monotypy and original designation, keyed. Brues 1908: 50 (diagnosis, list of species); Kieffer 1910b: 65, 85, 86 (description, list of species, keyed); Kieffer 1913a: 232 (description); Dodd 1920: 321 (diagnosis, taxonomic status); Kieffer 1926: 272, 488 (description, keyed); Dodd 1933: 75, 81 (comparison with *Macroteleia* Westwood, key to species of Australia); Muesebeck and Walkley 1951: 705 (catalog of species of U.S. and Canada); Muesebeck and Walkley 1956: 391 (citation of type species); Masner 1964: 137 (junior synonym of *Macroteleia* Westwood).
Stictoteleia Kieffer 1926: 272, 546. Original description. Type: *Macroteleia virginiensis* Ashmead, by original designation, keyed. Muesebeck and Walkley 1951: 706 (catalog of species of U.S. and Canada); Muesebeck and Walkley 1956: 400 (citation of type species); Masner 1964: 137 (junior synonym of *Macroteleia* Westwood).

#### Diagnosis.

*Macroteleia* is known as one of the longest and often the largest wasps in the subfamily Scelioninae. It may be distinguished from other genera of the subfamily by the combination of the following characters: marginal vein (R) distinctly elongate, as long as or longer than stigmal vein (r-rs) (Plate 4); propodeum without armature, divided into two separated halves (Plates 14B, 16D, 17D, 37B, 38D, 40B, 52B, 54B, 57B, 63B) or continuous medially (Plates 3A, 18D, 20B, 23B, 25D, 31D, 32C, 34B, 43B, 45B, 49B, 65B, 66D, 68B); wings hyaline or subhyaline (Plate 4), rarely with infuscations or darkening (Plate 60D); T6 in females strongly compressed laterally to form a wedge (Plate 5B); apex of T7 in males differentiated, truncate (Plates 6A, 8D, 10D, 12D, 35B, 47F, 50B, 55B, 58B, 61B) or excavate medially in New World species, pointed medially (Plates 6C, 15B, 21B, 24B, 26B, 28E, 41B, 46B, 69B) or cut off in Old World species, but never bidentate or bispinose (Masner 1976; Muesebeck 1977).

**Plate 4.**
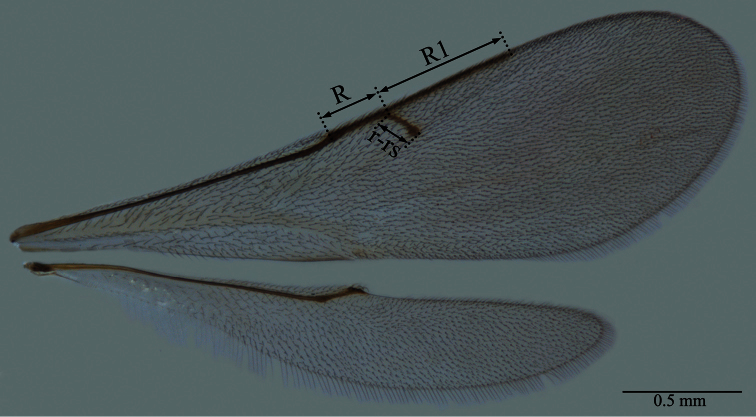
*Macroteleia striativentris* Crawford, fore and hind wing, female. R: marginal vein; R1: postmarginal vein; r-rs: stigmal vein.

**Plate 5.**
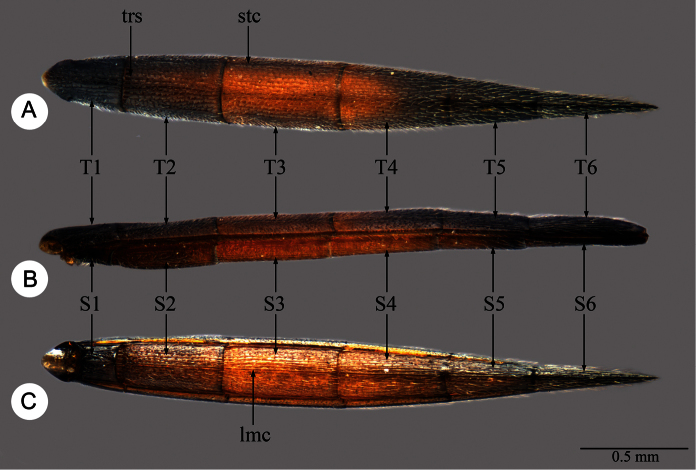
*Macroteleia striativentris* Crawford, female. **A** Metasoma, dorsal view **B** Metasoma, lateral view **C** Metasoma, ventral view. lmc: longitudinal median sternal carina; S1, S2, … S6: metasomal sternite 1–6; stc, sublateral tergal carina; trs: transverse sulcus of T2; T1, T2,... T6: metasomal terga 1–6.

**Plate 6.**
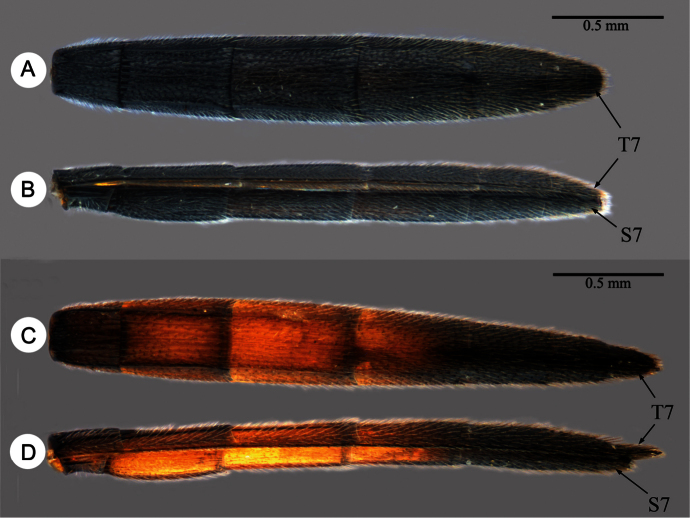
**A** *Macroteleia boriviliensis* Saraswat, male, metasoma, dorsal view **B** *Macroteleia boriviliensis* Saraswat, male, metasoma, lateral view **C** *Macroteleia striativentris* Crawford, male, metasoma, dorsal view **D** *Macroteleia striativentris* Crawford, male, metasoma, lateral view. S7: metasomal sternite 7; T7: metasomal tergite 7.

#### Comments.

*Macroteleia* is mostly similar to *Triteleia* that found in China both in size and body shape. Masner (1976) mentioned that the two genera were often confused, but *Triteleia* can be distinguished by the fact that T6 in females strongly depressed dorsoventrally to form a flat triangle, often spined at the apex; T7 in males armed posterolaterally with 2 sharp spikes or at least tyiny points; propodeum often armed dorsally, protruded into teeth or at least dorsal points or forms a triangular protuberance. *Habroteleia* is also similar to *Macroteleia*, but can bedistinguished by having postmarginal vein (R1) absent or only rudimentary; propodeum spined posteromedially and at posterolateral corners; T6 in females strongly depressed dorsoventrally to form a flat triangle as in *Triteleia*. The following key is used to separate *Triteleia* and *Habroteleia* from *Macroteleia* with the fewest characters possible.

##### Key to separate *Macroteleia*, *Triteleia* and *Habroteleia*

**Table:** 

1	Postmarginal vein in fore wing absent; female ovipositor Ceratobaeus-type	*Habroteleia* Kieffer
–	Postmarginal vein in fore wing well developed, distinctly longer than stigma vein (r-rs); female ovipositor Scelio-type	2
2	Female T6 strongly compressed, wedge like; male apical tergite apically emarginated or with 1 central spine but never bispinose	*Macroteleia* Westwood
–	T6 flatly triangular, not compressed; male apical tergite with postero-lateral corners bispinose or at least pointed	*Triteleia* Kieffer

##### Key to species of the genus *Macroteleia* from China

**Females**

(Unknown for *Macroteleia carinigena*, *Macroteleia gracilis*, *Macroteleia boriviliensis* and *Macroteleia spinitibia*)

**Table:** 

1	Propodeum divided into two separated subtriangular lobes (Plates 14B, 16D, 17D, 37B, 38D, 40B, 52B, 54B, 57B, 63B)	2
–	Propodeum continuous medially, not divided into two separated lobes (Plates 3A, 18D, 20B, 23B, 25D, 31D, 32C, 34B, 43B, 45B, 49B, 65B, 66D, 68B)	7
2	Head and mesosoma variably yellow to dark brown or orange (Plates 52B, 57B)	3
–	Head and mesosoma entirely black (Plates 14B, 37B, 38D, 40B, 54B, 65B, 66D, 68B)	4
3	Metascutellum triangular, strongly produced medially (Plate 52B); central keel weakly developed above interantennal process (Plate 52A)	*Macroteleia rufa* Szelényi
–	Metascutellum semicircular (Plate 57B); central keel well developed, extending onto interantennal process, slightly bifurcating dorsally (Plate 57A)	*Macroteleia semicircula* sp. n.
4	Central keel well developed, extending onto interantennal process (Plates 2A, 54A); legs robust, hind femur strongly swollen (Plates 53B, 62B)	5
–	Central keel weakly developed above interantennal process (Plates 14A, 37A, 38C, 40A); legs slender, hind femur weakly swollen (Plates 13B, 17B, 36B, 38B, 39B)	6
5	Mesopleural depression smooth (Plate 54C); occipital carina interrupted medially (Plate 54B); hind coxa dark brown to nearly black (Plate 54C)	*Macroteleia salebrosa* sp. n.
–	Mesopleural depression longitudinally striate (Plate 63B); occipital carina continuous medially (Plate 2B); hind coxa yellow (Plate 63B)	*Macroteleia striatipleuron* sp. n.
6	Metascutellum tongue-like (Plates 14B, 16D, 17D); metapleuron longitudinally striate dorsally, densely punctate ventrally (Plates 14C, 16E, 17E)	*Macroteleia crawfordi* Kieffer
–	Metascutellum triangular, strongly produced medially (Plates 37B, 38D, 40B); metapleuron longitudinally striate throughout (Plates 37C, 40C)	*Macroteleia lamba* Saraswat & Sharma
7	Head yellow or orange (Plates 25D, 29D, 31D, 32C, 34B)	8
–	Head entirely black (Plates 18D, 20B, 23B, 43B, 45B, 49B, 65B, 66D, 68B)	9
8	Length of T3 1.11–1.39× length of T6; T5 distinctly wider than long (Plates 30C, 31F, 34D); apex of fore wing extending from as far as mid-length of T5 to base of T6	*Macroteleia indica* Saraswat & Sharma
–	Length of T3 0.93–0.99× length of T6; T5 distinctly longer than wide (Plate 25F); apex of fore wing extending as far as posterior third of T4	*Macroteleia flava* sp. n.
9	Metasoma mixed brown and black (Plates 18D, 20D, 65D, 66F, 68D)	10
–	Metasoma entirely black (Plates 23D, 43D, 45D, 49D)	11
10	Frons below median ocellus and vertex densely punctate (Plates 65A, 65B, 66C, 66D, 68A, 68B); metapleuron longitudinally striate (Plates 65C, 66E, 68C); mesosoma black (Plates 65B, 66D, 68B)	*Macroteleia striativentris* Crawford
–	Frons below median ocellus and posterior vertex punctate reticulate (Plates 18C, 18D, 20A, 20B); metapleuron longitudinally striate dorsally, punctate rugulose ventrally (Plate 20C); mesosoma orange (Plates 18D, 20B)	*Macroteleia dolichopa* Sharma
11	Ocellar triangle densely punctate; lower mesepisternum densely punctate to punctate rugulose; posterior margin of transverse sulcus on T2 straight (Plate 49D)	*Macroteleia peliades* Kozlov & Lê
–	Ocellar triangle largely smooth, with scattered punctures; lower mesepisternum variably smooth to punctate rugulose; posterior margin of transverse sulcus on T2 convex (Plates 23D, 43D, 45D)	12
12	Gena punctate rugose; body length 5.48–6.14 mm	*Macroteleia emarginata* Dodd
–	Gena punctate reticulate; body length 3.28–4.08 mm	*Macroteleia livingstoni* Saraswat

**Males**

(Unknown for *Macroteleia striatipleuron* and *Macroteleia rufa*)

**Table:** 

1	T7 transverse, apex truncate (Plates 6A, 8D, 10D, 12D, 35B, 47F, 50B, 55B, 58B, 61B)	2
–	T7 subtriangular, apex pointed (Plates 6C, 15B, 21B, 24B, 26B, 28E, 41B, 46B, 69B)	8
2	Central keel absent; legs slender, hind femur weakly swollen	5
–	Central keel well developed, extending onto interantennal process; legs robust, hind femur strongly swollen	3
3	Hind tibia without spines over outer surface (Plates 55A, 58A); mesopleural depression smooth	4
–	Hind tibia with numerous semi-erect, yellow spines over outer surface (Plate 61D); mesopleural depression longitudinally striate (Plate 60C)	*Macroteleia spinitibia* sp. n.
4	Metascutellum rectangular (Plate 55A); fore wing hyaline throughout	*Macroteleia salebrosa* sp. n.
–	Metascutellum semicircular (Plate 58A); fore wing slightly infuscate in basal half	*Macroteleia semicircula* sp. n.
5	T6 longer than wide (Plates 47F, 50B, 55B); body length 7.00–8.00 mm	*Macroteleia peliades* Kozlov & Lê
–	T6 wider than long (Plates 8D, 10D, 12D, 35B); body less than 7.00 mm	6
6	Gena with a strong carina parallel to occipital carina, punctate rugose dorsally (Plate 12B); propodeal lobe without longitudinally carinae	*Macroteleia carinigena* sp. n.
–	Gena without strong carina, punctate rugose throughout (Plates 8B, 10B); propodeal lobe with several irregular longitudinal carinae medially	7
7	Head and mesosoma black; metapleuron longitudinally striate dorsoventrally, punctate rugulose medially	*Macroteleia boriviliensis* Saraswat
–	Head and mesosoma yellow or orange; metapleuron longitudinally striate throughout	*Macroteleia indica* Saraswat & Sharma
8	Lateral ocellus contiguous with inner orbit of compound eye	9
–	Lateral ocellus separated from inner orbit of compound eye	11
9	T7 sharply pointed medially (Plate 21B); mesosoma orange yellow or brown (Plate 21A)	*Macroteleia dolichopa* Sharma
–	T7 bluntly pointed medially (Plates 26B, 69B); mesosoma entirely black (Plates 41A, 46A)	10
10	Frons below median ocellus densely punctate, interspaces coriaceous; ocellar triangle coriaceous, with scattered punctures	*Macroteleia lamba* Saraswat & Sharma
–	Frons below median ocellus sparsely punctate, interspaces smooth; ocellar triangle smooth, with scattered punctures	*Macroteleia livingstoni* Saraswat
11	T6 wider than long (Plate 15B); apex of T7 sharply pointed to form a spine (Plate 15B)	*Macroteleia crawfordi* Kieffer
–	T6 longer than wide (Plates 24B, 26B, 28E, 69B); apex of T7 pointed, but not forming a spine (Plates 24B, 26B, 28E, 69B)	12
12	Head brown (Plates 26A, 69A)	13
–	Head entirely black (Plates 24A, 28B)	14
13	Length of A3 0.83–0.90× length of A2; mesosoma black (Plate 69A)	*Macroteleia striativentris* Crawford
–	Length of A3 equal to length of A2; mesosoma orange yellow (Plate 26A)	*Macroteleia flava* sp. n.
14	Metapleuron longitudinally striate dorsally, punctate rugulose ventrally; length of T7 1.42–1.87× length of S7 (Plate 24C)	*Macroteleia emarginata* Dodd
–	Metapleuron longitudinally striate throughout; length of T7 2.50–3.22× length of S7 (Plate 28F)	*Macroteleia gracilis* sp. n.

### Macroteleia boriviliensis

Saraswat

http://species-id.net/wiki/Macroteleia_boriviliensis

Plates 7 –10

Macroteleia boriviliensis Saraswat 1982: 344 (original description).

#### Description.

*Male*. Body length 3.77–5.41 mm (n=20).

*Color*. Body black, metasoma somewhat brownish; mandible dark brown; palpi yellow; legs pale brown, becoming darker distally; A1 brown, A2–A4 dark brown, remainder of antenna black; fore wing subhyaline.

*Head*. Transverse in dorsal view, 1.30–1.56× as wide as long, slightly wider than mesosoma; OOL short, 0.13–0.29× minimum ocellar width; POL 1.36–1.58× LOL; occipital carina weakly continuous medially, irregularly punctate; central keel absent (Plates 8C, 10C); medial frons contiguously punctate ventrally, with irregularly shaped smooth area dorsally; frons below median ocellus contiguously punctate; vertex densely punctate, interspaces in part with microsculpture; gena punctate rugose; length of A3 1.00–1.12× length of A2.

*Mesosoma*. Cervical pronotal area densely punctate; dorsal pronotal area areolate; lateral pronotal area smooth dorsally, punctate rugulose ventrally; netrion punctate rugulose; notaulus distinctly foveolate; middle lobe of mesoscutum densely punctate, becoming denser anteriorly and posteriorly; lateral lobes of mesoscutum irregularly punctate; mesoscutellum densely finely punctate throughout; metascutellum transverse, posterior margin straight, longitudinally carinate (Plates 8A, 10A); propodeum continuous medially (Plates 8A, 10A), not divided into two separated lobes, posterior margin narrowly notched medially, each side with several irregular longitudinal carinae medially, otherwise punctate rugulose, covered by dense, recumbent, white setae; upper mesepisternum with a row of somewhat robust longitudinal carinae below subalar pit; lower mesepisternum longitudinally punctate rugulose; mesopleural depression smooth or finely longitudinally striate (Plates 8B, 10B); metapleuron longitudinally striate dorsoventrally, punctate rugulose medially.

*Legs*. Slender; hind femur weakly swollen, 3.75–3.95× as long as its maximum width; hind tibia without spines over outer surface; hind basitarsus 10.83–11.60× as long as its maximum width.

*Wings*. Apex of fore wing extending from as far as posterior fifth of T4 to base of T5; R 1.59–1.94× as long as r-rs, R1 1.91–2.2× length of R.

*Metasoma*. Posterior margin of transverse sulcus on T2 slightly convex (Plates 8F, 10F); sublateral tergal carinae developed on T1–T4; T1–T4 densely longitudinally striate medially, with numerous delicate punctures in interstices, punctate rugulose laterally; T5–T7 longitudinally punctate rugulose throughout; T6 distinctly wider than long; length of T6 2.33–3.00× length of T7; T7 transverse, apex truncate (Plates 8D, 10D); length of T7 0.64–0.88× length of S7; S2–S5 sparsely longitudinally striate, with numerous delicate punctures in interstices; S6–S7 weakly punctate rugulose; prominent longitudinal median carinae present on S2–S5.

*Female*. Unknown.

**Plate 7.**
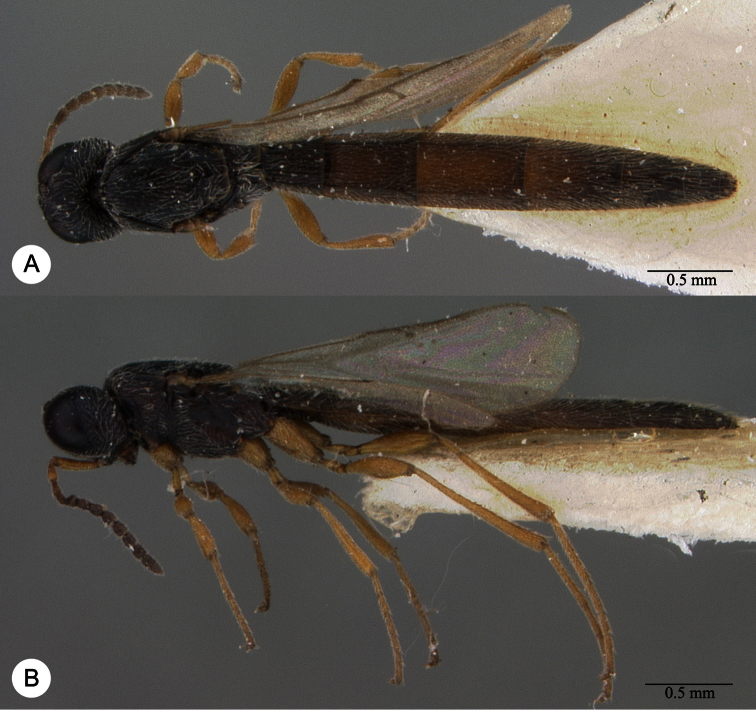
*Macroteleia boriviliensis* Saraswat, holotype, male. **A** Dorsal habitus **B** Lateral habitus.

**Plate 8.**
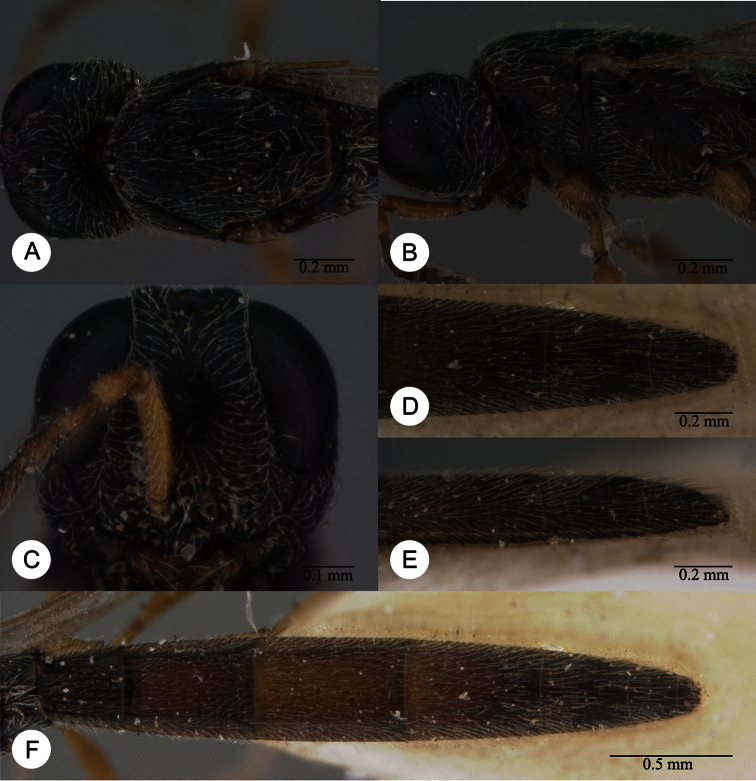
*Macroteleia boriviliensis* Saraswat, holotype, male. **A** Head and mesosoma, dorsal view **B** Head and mesosoma, lateral view **C** Head, anterior view **D** Apex of metasoma, dorsal view **E** Apex of metasoma, lateral view **F** Metasoma, dorsal view.

**Plate 9.**
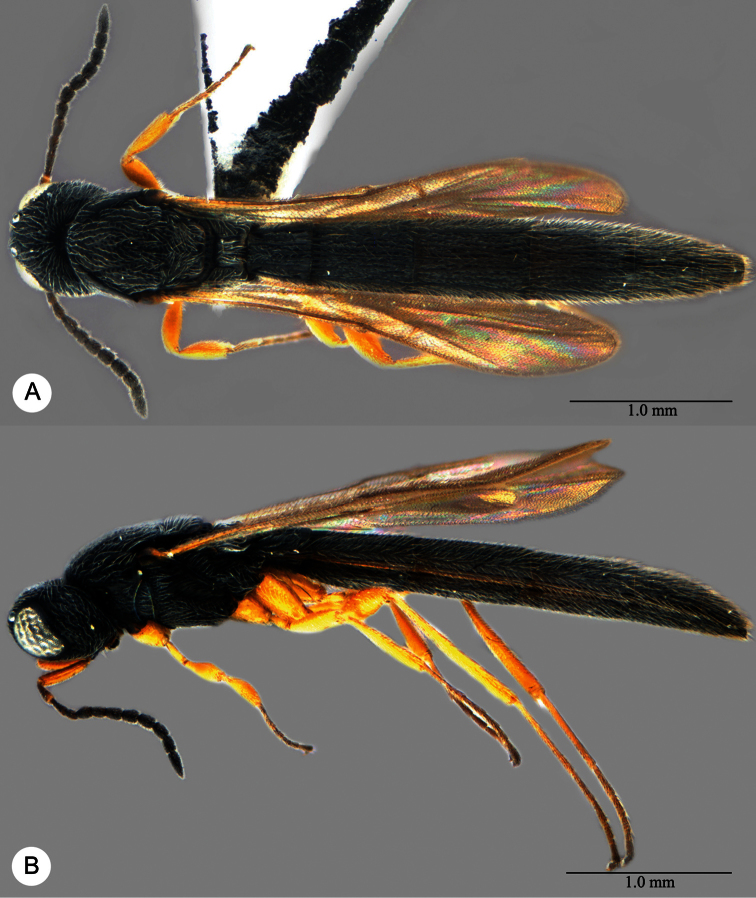
*Macroteleia boriviliensis* Saraswat, male from Guangdong, Fogang, Mt. Guanyin. **A** Dorsal habitus **B** Lateral habitus.

**Plate 10.**
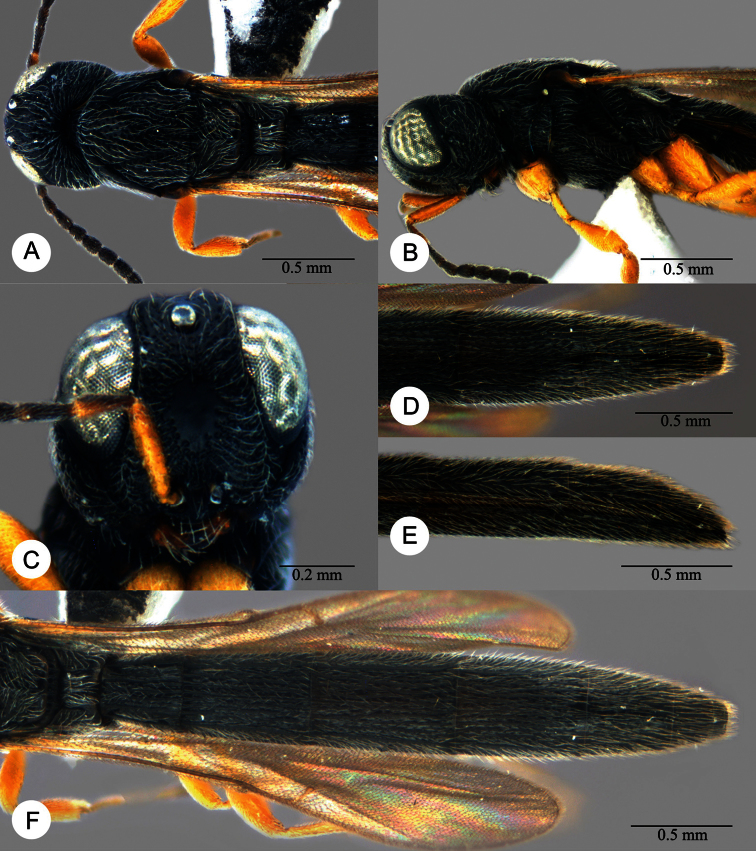
*Macroteleia boriviliensis* Saraswat, male from Guangdong, Fogang, Mt. Guanyin. **A** Head and mesosoma, dorsal view **B** Head and mesosoma, lateral view **C** Head, anterior view **D** Apex of metasoma, dorsal view **E** Apex of metasoma, lateral view **F** Metasoma, dorsal view.

#### Distribution.

China (Guangdong, Hainan, Guangxi, Yunnan); Thailand; India. Link to distribution map [http://hol.osu.edu/map-large.html?id=4784].

#### Material examined.

*Holotype*, ♂: **INDIA**: “School of Entomology, St. John’s College, Agra-2, India”, “4–13. Borivili: Bombay, Coll. Mani & Party, 25.IX.1971”, “Holotype”, “*Macroteleia boriviliensis* Saraswat, ♂” (deposited in USNM).

#### Other material.

**CHINA**: 1 ♂, Guangdong, Nanling National Nature Reserve, 24°54'N, 113°00'E, 4.VIII.2004, Jingxian Liu, SCAU 000057 (SCAU); 1 ♂, Guangdong, Zijin County, Linjiang Town, 23°39'N, 114°41'E, 1.VIII.2003, Jingxian Liu, SCAU 000058 (SCAU); 1 ♂, Guangdong, Fogang, Mt. Guanyin, 23°57'N, 113°32'E, 15.V.2004, Zaifu Xu, SCAU 000059 (SCAU); 2 ♂, Guangdong, Zhaoqing, Xiwanggu, 23°13'N, 112°31'E, 2–6.VIII.2010, sweeping, Huayan Chen, SCAU 000060, 000061 (SCAU); 1 ♂, Guangdong, Zhaoqing, Xiwanggu, 23°13'N, 112°31'E, 2–6.VIII.2010, yellow pan trap, Huayan Chen, SCAU 000062 (SCAU); 1 ♂, Guangdong, Guangzhou, Tianlu Lake, 23°13'N, 113°25'E, 6.X.2002, Zaifu Xu, SCAU 000063 (SCAU); 1 ♂, Guangdong, Suixi County, Huanglue Town, 21°20.36'N, 110°18.61'E, 25.IX.2010, yellow pan trap, Huayan Chen, SCAU 000064 (SCAU); 1 ♂, Hainan, Mt. Yinggeling, 18°49'N, 109°11'E, 28.V.2007, Liqiong Weng, SCAU 000065 (SCAU); 1 ♂, Hainan, Mt. Yinggeling, 18°49'N, 109°11'E, 17–20.VII.2010, Huayan Chen, SCAU 000066 (SCAU); 1 ♂, Hainan, Jianfengling National Nature Reserve, 18°41'N, 108°49'E, 4.V.2008, Huayan Chen, SCAU 000067 (SCAU); 1 ♂, Hainan, Mt. Diaoluo, 18°39'N, 109°53'E, 28.V–1.VI.2007, Jie Zeng, SCAU 000068 (SCAU); 1 ♂, Hainan, Mt. Diaoluo, 18°39'N, 109°53'E, 29.V.2007, Bin Xiao, SCAU 000069 (SCAU); 1 ♂, Guangxi, Guilin, Maoershan National Nature Reserve, 25°53'N, 110°25'E, 2–10.VIII.2005, Bin Xiao, SCAU 000070 (SCAU); 4 ♂, Yunnan, Gaoligongshan National Nature Reserve, 24°49'N, 98°46'E, 1.VIII.2005, Yali Cai, SCAU 000071–000074 (SCAU); 1 ♂, Yunnan, Jinggu County, Weiyuan Town, 23°29'N, 100°42'E, 4.X.2004, Jingxian Liu, SCAU 000075 (SCAU); 2 ♂, Yunnan, Nabanhe Basin National Nature Reserve, 22°15.47'N, 100°36.32'E, 892M, 19–23.VII.2011, yellow pan trap, Zaifu Xu, SCAU000076, 000077 (SCAU). **THAILAND**: 1 ♂, Chiang Mai, Maerim, 20.XII.2002, flight intercept trap, R. A. Beaver, No. 25926 (RABC).

### Macroteleia carinigena sp. n.

urn:lsid:zoobank.org:act:42427976-EE7B-4B81-8910-EF308AE8716E

http://species-id.net/wiki/Macroteleia_carinigena

Plates 11 –12

#### Description.

*Male*. Body length 6.41–6.62 mm (n=3).

*Color*. Body black; mandible reddish brown; palpi yellow; legs yellow throughout; A1–A2 yellow, remainder of antenna brown to dark brown; fore wing hyaline.

*Head*. Transverse in dorsal view, 1.56–1.70× as wide as long, slightly wider than mesosoma; lateral ocellus contiguous with inner orbit of compound eye; POL 1.58–1.67× LOL; occipital carina continuous medially, irregularly crenulate throughout; central keel absent (Plate 12C); medial frons punctate with irregularly shaped smooth area; ventrolateral frons punctate rugulose to densely punctate; frons below median ocellus punctate reticulate; vertex densely punctate with punctures in part contiguous; gena with a strong carina parallel to occipital carina (Plate 12B), punctate rugose dorsally; length of A3 1.30–1.33× length of A2.

*Mesosoma*. Cervical pronotal area densely punctate; dorsal pronotal area areolate; lateral pronotal area smooth dorsally, irregularly depressed ventrally; netrion densely finely punctate; notaulus shallow, irregularly foveolate; middle lobe of mesoscutum densely punctate, becoming denser anteriorly; lateral lobes of mesoscutum densely punctate throughout; mesoscutellum densely punctate, becoming denser laterally; metascutellum transverse, posterior margin slightly pointed medially, longitudinally carinate (Plate 12A); propodeum continuous medially (Plate 12A), not divided into two separated lobes, posterior margin narrowly notched medially, each side with rugose sculpture covered by dense, recumbent, white setae; upper mesepisternum with a row of robust longitudinal carinae below subalar pit; lower mesepisternum densely punctate rugulose; mesopleural depression smooth (Plate 12B); metapleuron longitudinally striate with coarse punctures in interstices, or longitudinally punctate rugose.

*Legs*. Slender; hind femur weakly swollen, 4.00–4.55× as long as its maximum width; hind tibia without spines over outer surface; hind basitarsus 7.67–9.00× as long as its maximum width.

*Wings*. Apex of fore wing extending from as far as basal fifth to mid-length of T5; R 1.46–1.60× as long as r-rs, R1 1.95–2.43× length of R.

*Metasoma*. Posterior margin of transverse sulcus on T2 strongly convex (Plate 12A); sublateral tergal carinae well developed on T1–T3, weakly developed on anterior half of T4; T1–T3 sparsely longitudinally striate medially, with delicate punctures in interstices, punctate rugulose laterally; T4–T6 densely longitudinally striate, with numerous delicate punctures in interstices; T7 finely punctate rugulose posteriorly; T6 slightly wider than long; length of T6 2.35–2.63× length of T7; T7 transverse, apex truncate (Plate 12D); length of T7 equal to length of S7; S2–S3 sparsely longitudinally striate, with scattered delicate punctures and irregular microsculpture in interstices; S4–S6 densely longitudinally striate, with numerous delicate punctures in interstices; S7 densely punctate rugulose throughout; prominent longitudinal median carinae present on S2–S5.

*Female*. Unknown.

**Plate 11.**
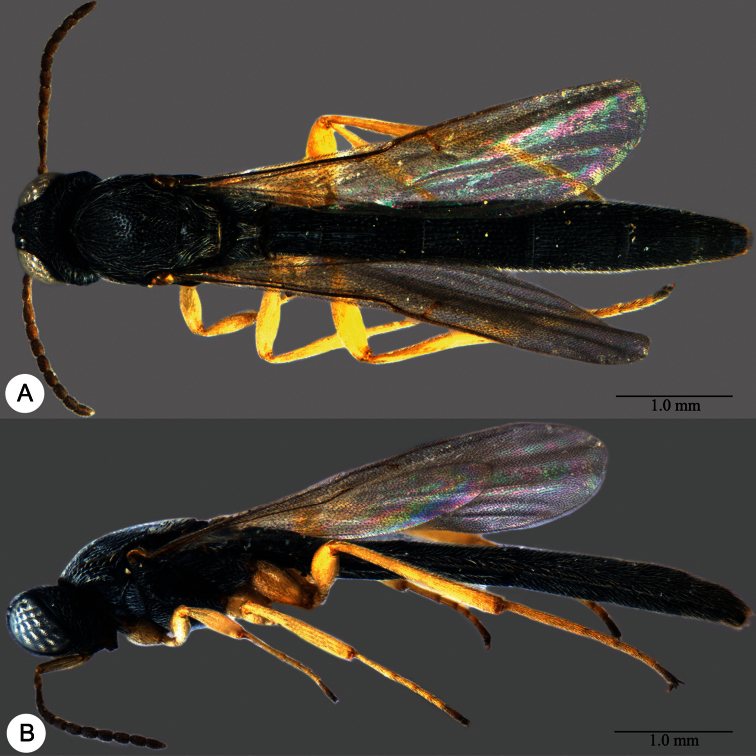
*Macroteleia carinigena* sp. n., holotype, male. **A** Dorsal habitus **B** Lateral habitus.

**Plate 12.**
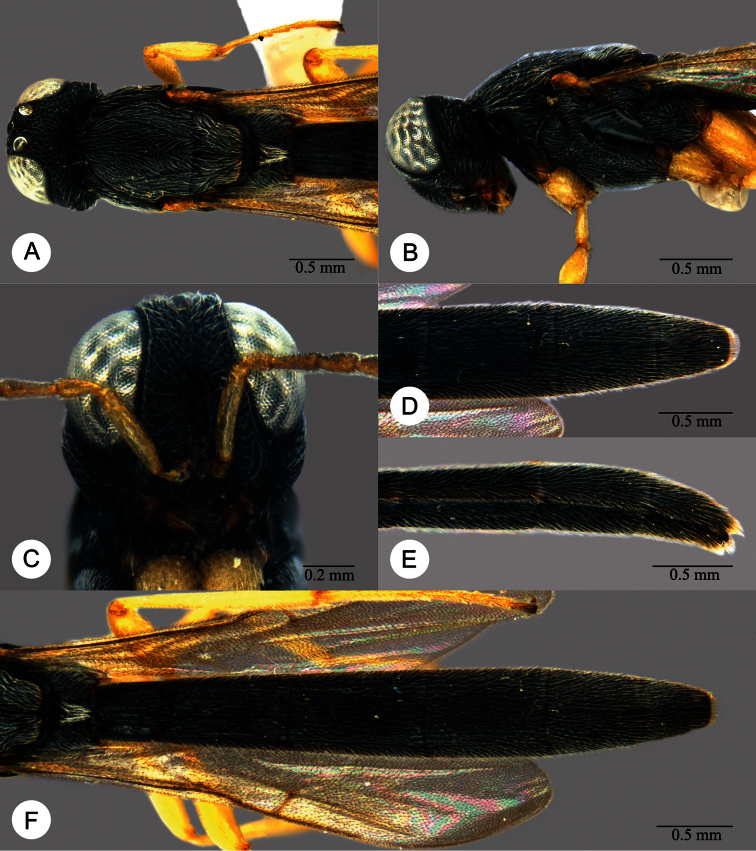
*Macroteleia carinigena* sp. n., holotype, male. **A** Head and mesosoma, dorsal view **B** Head and mesosoma, lateral view **C** Head, anterior view **D** Apex of metasoma, dorsal view **E** Apex of metasoma, lateral view **F** Metasoma, dorsal view.

#### Diagnosis.

This species is similar to *Macroteleia boriviliensis*, but can be distinguished by the presence of a strong carina on the gena (no carina in *Macroteleia boriviliensis*) and the propodeal lobe without longitudinal carinae (with several irregular longitudinal carinae medially in *Macroteleia boriviliensis*).

#### Etymology.

The name *carinigena* refers to the strong carina present on gena of this species. The epithet is used as a noun in apposition.

#### Distribution.

China (Hainan). Link to distribution map [http://hol.osu.edu/map-large.html?id=320503].

#### Material examined.

*Holotype*, ♂: **CHINA**: Hainan, Mt. Yinggeling, 18°49'N, 109°11'E, 28.V.2007, Liqiong Weng, SCAU 000032 (deposited in SCAU). *Paratypes*: 1 ♂, Hainan, Mt. Diaoluo, 18°39'N, 109°53'E, 29.V.2007, Bin Xiao, SCAU 000033 (SCAU); 1 ♂, Hainan, Mt. Diaoluo, 18°39'N, 109°53'E, 29.V.2007, Jingxian Liu, SCAU 000034 (SCAU).

### Macroteleia crawfordi

Kieffer stat. n.

Plates 13 –17

Macroteleia kiefferi Crawford 1910: 127 (original description, preoccupied by *Macroteleia kiefferi* Brues, (1906) ); Baltazar 1966: 184 (listed, junior synonym of *Macroteleia striativentris* Crawford).
Macroteleia crawfordi Kieffer 1910b: 89 (replacement name for *Macroteleia kiefferi* Crawford); Baltazar 1966: 184 (listed, junior synonym of *Macroteleia striativentris* Crawford); Masner and Muesebeck 1968: 39 (type information).
Macroteleia crates Lê 2000: 53, 55, 331 (original description, keyed), **syn. n.**
Macroteleia demades Lê 2000: 53, 56, 332 (original description, keyed), **syn. n.**

#### Description.

*Female*. Body length 3.35–4.76 mm (n=20).

*Color*. Body black; mandible brown; palpi yellow; legs pale brown throughout; A1 brown, A2–A5 dark brown, remainder of antenna black; fore wing hyaline.

*Head*. Transverse in dorsal view, 1.30–1.40× as wide as long, slightly wider than mesosoma; OOL short, 0.20–0.40× times minimum diameter of lateral ocellus; POL 1.27–1.58× LOL; occipital carina continuous medially, irregularly crenulate; central keel weakly developed above interantennal process (Plates 14A, 16C); medial frons obliquely strigose ventrally, variably smooth to coriaceous dorsally; ventrolateral frons punctate rugose; frons below median ocellus densely punctate, interspaces coriaceous; ocellar triangle smooth or coriaceous, with scattered punctures; posterior vertex densely punctate, interspaces coriaceous; gena coarsely punctate rugose; length of A3 0.71–0.86× length of A2.

*Mesosoma*. Cervical pronotal area densely punctate; dorsal pronotal area areolate; lateral pronotal area punctate rugulose; netrion densely punctate or punctate rugulose; notaulus deep, distinctly foveolate; middle lobe of mesoscutum densely punctate, becoming denser anteriorly; lateral lobes of mesoscutum densely punctate throughout; mesoscutellum densely punctate throughout; metascutellum tongue-like (Plates 14B, 16D, 17D), extending into space between propodeal lobes; propodeum narrowly divided into two widely separated subtriangular lobes (Plates 14B, 16D, 17D), each side with punctate rugulose sculpture covered by dense, recumbent, white setae; upper mesepisternum with a row of robust longitudinal carinae below subalar pit; lower mesepisternum densely punctate; mesopleural depression smooth (Plates 14C, 16E, 17E); metapleuron longitudinally striate dorsally, densely punctate ventrally.

*Legs*. Slender; hind femur weakly swollen, 4.06–4.71× as long as its maximum width; hind tibia without spines over outer surface; hind basitarsus 11.00–11.75× as long as its maximum width.

*Wings*. Apex of fore wing extending from as far as posterior margin of T4 to base of T5; R 1.79–2.55× as long as r-rs, R1 1.18–1.77× length of R.

*Metasoma*. Posterior margin of transverse sulcus on T2 straight or slightly convex (Plate 14D); sublateral tergal carinae developed on T1–T4; T1 densely longitudinally striate medially, with numerous punctures in interstices anteriorly, punctate rugulose laterally; T2–T4 densely longitudinally striate medially, with scattered delicate punctures in interstices, punctate rugulose laterally; T5 densely longitudinally striate throughout, with delicate punctures in interstices; T6 finely punctate dorsally, densely longitudinally striate laterally, with scattered delicate punctures in interstices; length of T3 0.82–0.99× length of T6; T5 distinctly longer than wide; S2–S4 densely longitudinally striate, with delicate punctures in interstices; S5–S6 largely smooth, with scattered punctures; prominent longitudinal median carina present on S2–S5.

*Male*. Differing from female as follows: body length 3.57–3.85 mm (n=6); A1 brown, remainder of antenna dark brown to black; metascutellum distinctly transverse (Plate 15A), posterior margin straight, longitudinally carinate; propodeum continuous medially (Plate 15A), not divided into two separated lobes, posterior margin narrowly notched medially, each side with several irregular longitudinal carinae medially, otherwise punctate rugulose, covered by dense, recumbent, white setae; T1–T4 denselylongitudinally striate medially, with delicate punctures in interstices, punctate rugulose laterally; T5–T7 densely longitudinally striate throughout, with delicate punctures in interstices; T6 slightly wider than long; length of T6 1.00–1.13× length of T7; T7 subtriangular, apex sharply pointed to form a spine (Plate 15B, C); length of T7 1.13–1.77× length of S7; S2–S3 densely longitudinally striate, with punctate rugulose sculpture in interstices; S4–S6 densely longitudinally striate, with delicate punctures in interstices; S7 longitudinally punctate rugulose; prominent longitudinal median carina present on S2–S6.

**Plate 13.**
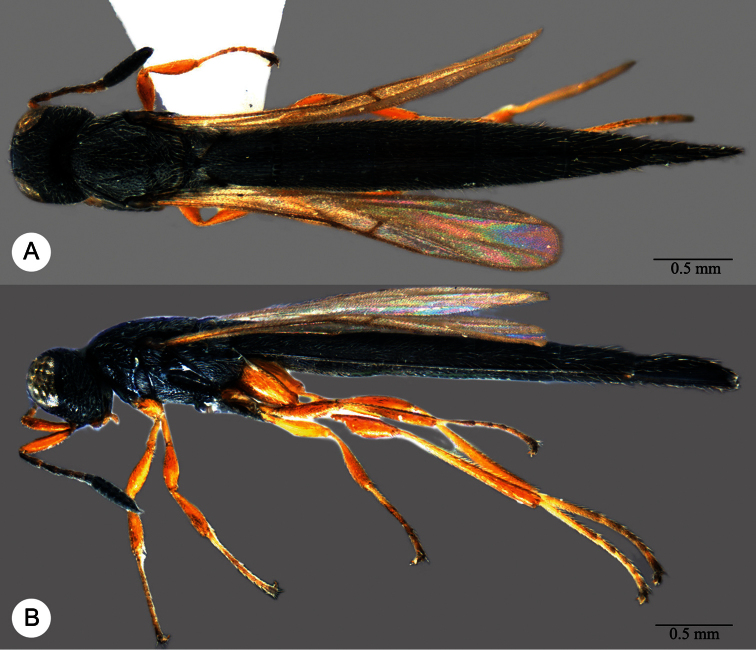
*Macroteleia crawfordi* Kieffer, female from Guangdong, Chebaling National Nature Reserve. **A** Dorsal habitus **B** Lateral habitus.

**Plate 14.**
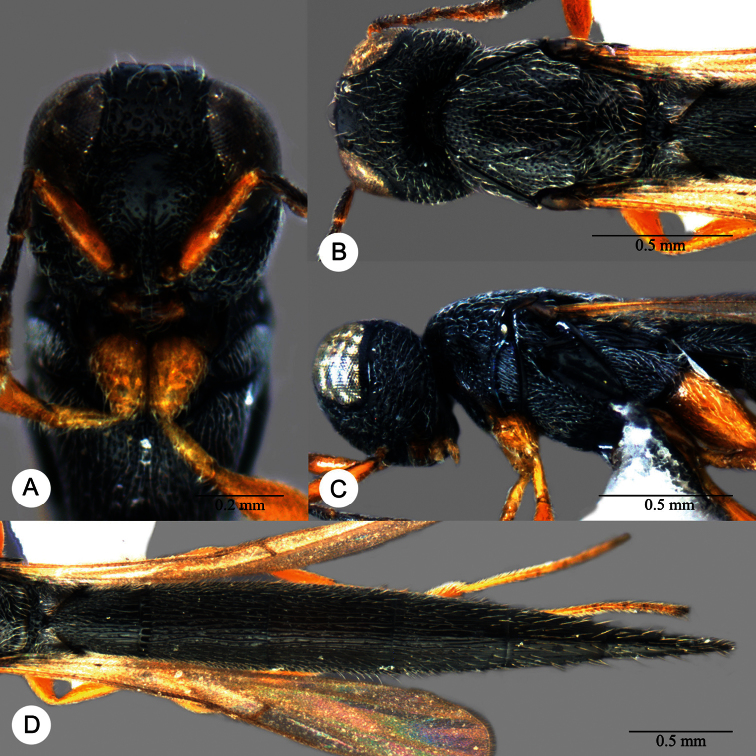
*Macroteleia crawfordi* Kieffer, female from Guangdong, Chebaling National Nature Reserve. **A** Head, anterior view **B** Head and mesosoma, dorsal view **C** Head and mesosoma, lateral view **D** Metasoma, dorsal view.

**Plate 15.**
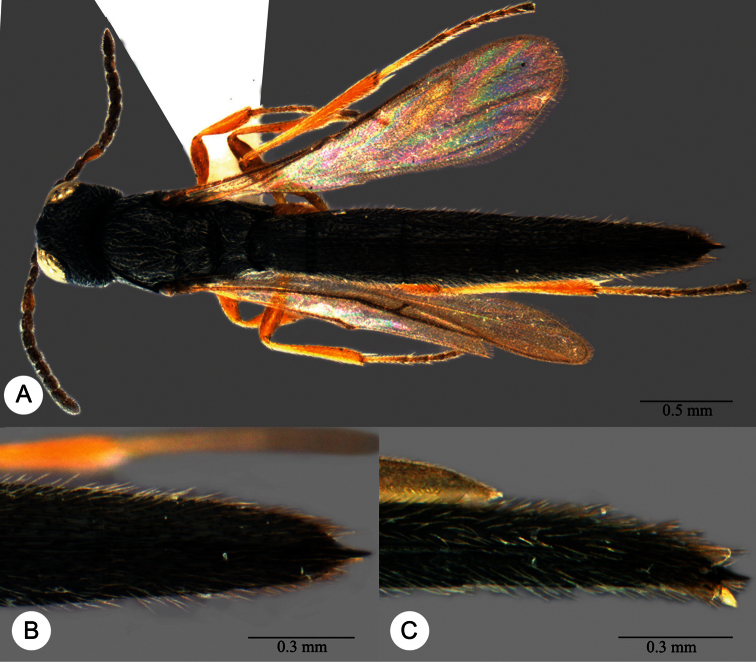
*Macroteleia crawfordi* Kieffer, male from Guangdong, Chebaling National Nature Reserve. **A** Dorsal habitus **B** Apex of metasoma, dorsal view **C** Apex of metasoma, lateral view.

**Plate 16.**
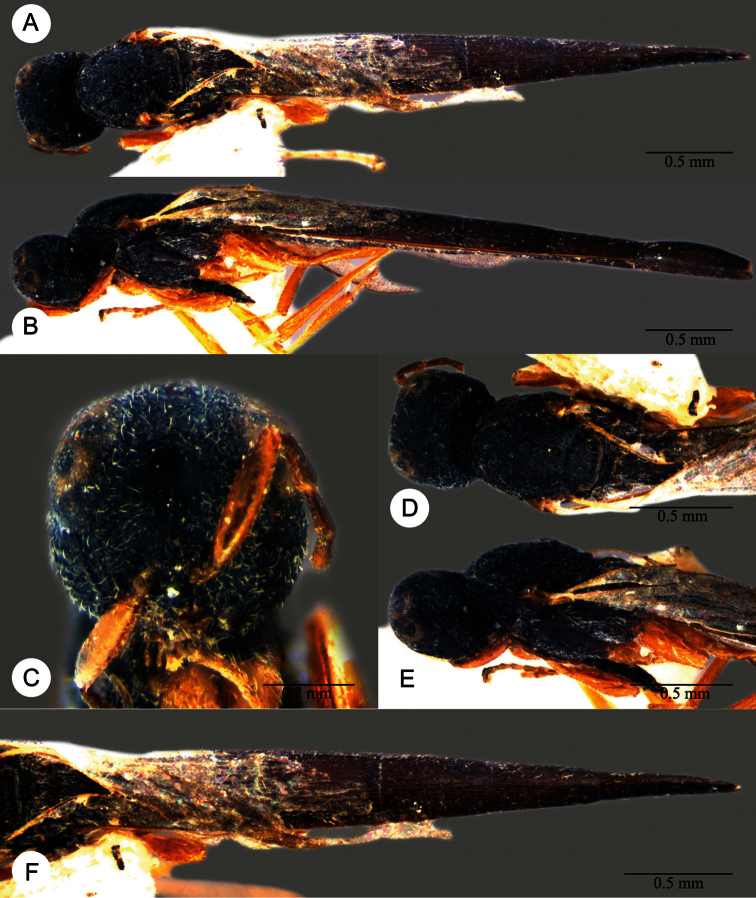
*Macroteleia crates* Kozlov & Lê, holotype, female. **A** Dorsal habitus **B** Lateral habitus **C** Head, anterior view **D** Head and mesosoma, dorsal view **E** Head and mesosoma, lateral view **F** Metasoma, dorsal view.

**Plate 17.**
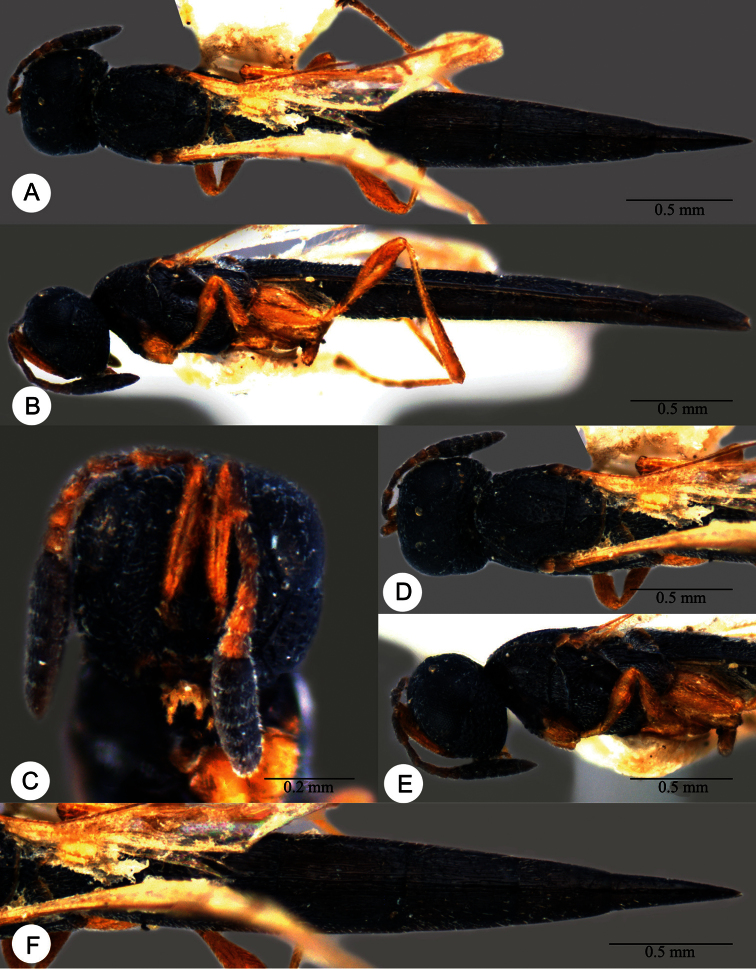
*Macroteleia demades* Kozlov & Lê, holotype, female. **A** Dorsal habitus **B** Lateral habitus **C** Head, anterior view **D** Head and mesosoma, dorsal view **E** Head and mesosoma, lateral view **F** Metasoma, dorsal view.

#### Diagnosis.

The body shape, color and size of *Macroteleia crawfordi* is similar to *Macroteleia lamba* and *Macroteleia livingstoni*. It differs from them by tongue-like metascutellum in female (triangular in *Macroteleia lamba*, and transverse in *Macroteleia linvingstoni*), and apex of T7 sharply pointed to form a spine in male (apex not pointed to form a spine in the latter two speices).

#### Distribution.

China (Guangdong, Hainan); Vietnam; Thailand; Philippines. Link to distribution map [http://hol.osu.edu/map-large.html?id=9295].

#### Material examined.

*Holotype* of *Macroteleia crawfordi*, ♀: **PHILIPPINES**: “Philippines, Manila, R. Brown, USNM No.12897” (deposited in USNM). *Holotype* of *Macroteleia crates*, ♀, **VIETNAM**: “8/4 construction site (Dak Lak Prov.), rice field, 15.V.1979”, “Holotypus ♀ *Macroteleia crates* Kozlov et Lê 84”. *Holotype* of *Macroteleia demades*, ♀ (deposited in IEBR): **VIETNAM**: “Nghia Do, Hanoi, on rice seedling, 24.VII.1978”, “Holotypus ♀ *Macroteleia demades* Kozlov et Lê 84” (deposited in IEBR). *Paratypes* of *Macroteleia demades*, 3 ♀ (IEBR): 2 ♀, **VIETNAM**: “Nghia Do, Hanoi, on rice seedling, 24.VII.1978, Lê Xuan Hue”, “Paratypus *Macroteleia demades* sp. n.”; 1 ♀, **VIETNAM**: “Ha Noi, rice field, 15.VII.1978”, “Paratypus *Macroteleia demades* sp. n.”.

**Other material. CHINA**: 1 ♀, Guangdong, Chebaling National Nature Reserve, 24°43'N, 114°14'E, 25.V.2002, Jingxian Liu, SCAU 000139 (SCAU); 10 ♀ + 1 ♂, Guangdong, Chebaling National Nature Reserve, 24°43'N, 114°14'E, 21–23.VIII.2003, Jingxian Liu, SCAU 000140–000150 (SCAU); 1 ♀ + 1 ♂, Guangdong, Zijin County, Linjiang Town, 23°39'N, 114°41'E, 1.VIII.2003, Jingxian Liu, SCAU 000151, 000152 (SCAU); 1 ♀, Guangdong, Mt. Tongleda, 23°10'N, 111°25'E, 12.VIII.2003, Jujian Chen, SCAU 000153 (SCAU); 1 ♀, Guangdong, Zhuhai, Doumen, paddy field, 22°33.366'N, 113°13.297'E, 11.IX.2010, Xin Yuan, SCAU 000154 (SCAU); 1 ♀, Guangdong, Zhaoqing, Xiwanggu, 23°13'N, 112°31'E, 2–6.VIII.2010, yellow pan trap, Huayan Chen, SCAU 000155 (SCAU); 2 ♀, Hainan, Wuzhishan National Nature Reserve, 18°51'N, 109°39'E, 16–18.V.2007, Jie Zeng, SCAU 000156, 000157 (SCAU); 1 ♀, Hainan, Wuzhishan National Nature Reserve, 18°51'N, 109°39'E, 17–20.V.2007, Bin Xiao, SCAU 000158 (SCAU); 1 ♀, Hainan, Wuzhishan National Nature Reserve, 18°51'N, 109°39'E, 20.V.2007, Jingxian Liu, SCAU 000159 (SCAU); 14 ♀ + 2 ♂, Hainan, Wuzhishan National Nature Reserve, 18°51'N, 109°39'E, 28.X–30.X.2007, Jiemin Yao, SCAU 000160–000175 (SCAU); 7 ♀, Hainan, Wuzhishan National Nature Reserve, 18°51'N, 109°39'E, 29.X.2007, Jingxian Liu, SCAU 000176–000182 (SCAU); 2 ♀, Hainan, Jianfengling National Nature Reserve, 18°41'N, 108°49'E, 23–25.X.2007, Jiemin Yao, SCAU 000183, 000184 (SCAU); 1 ♀, Hainan, Mt. Yinggeling, 18°49'N, 109°11'E, 16–20.XI.2008, Huayan Chen, SCAU 000185 (SCAU); 2 ♀ + 1 ♂, Hainan, Mt. Yinggeling, 18°49'N, 109°11'E, 17–20.VII.2010, Huayan Chen, SCAU 000186–000188 (SCAU). **THAILAND**: 1 ♀, Chiang Mai: Maerim, 29–30.XII.2000, MT, R. A. Beaver, No. 24139 (RABC); 1 ♀, Chiang Mai: Maerim, 24–25.I.2001, MT, R. A. Beaver, No. 24671 (RABC).

### Macroteleia dolichopa

Sharma

urn:lsid:zoobank.org:act:BF0454A0-EF33-4A82-BC87-33ACDF973231

http://species-id.net/wiki/Macroteleia_dolichopa

Plates 18 –21

Macroteleia dolichopa Sharma 1980: 300 (original description); Mani and Sharma 1982: 171 (description).

#### Description.

*Female*. Body length 5.13–7.50 mm (n=8).

*Color*. Head black; mesosoma orange yellow; metasoma with T1, T5 and T6 variably dark brown to black, otherwise orange yellow; mandible dark brown; palpi yellow; legs yellow throughout; A1–A6 yellowish brown, A7–A12 dark brown to black; fore wing hyaline.

**Plate 18.**
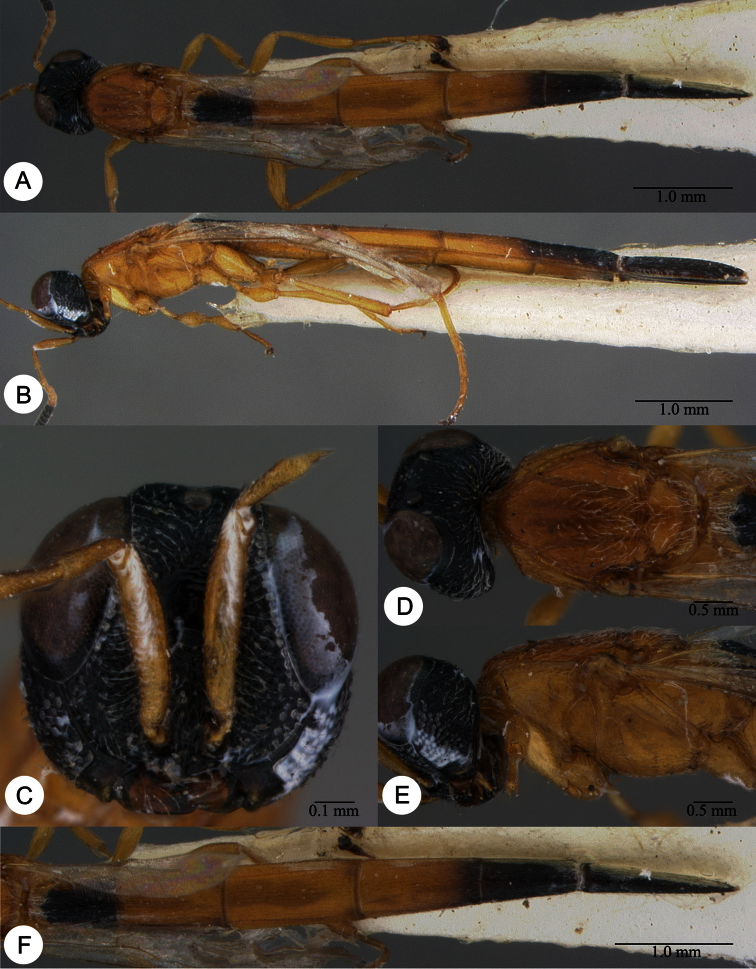
*Macroteleia dolichopa* Sharma, holotype, female. **A** Dorsal habitus **B** Lateral habitus **C** Head, anterior view **D** Head and mesosoma, dorsal view **E** Head and mesosoma, lateral view **F** Metasoma, dorsal view.

*Head*. Transverse in dorsal view, 1.42–1.44× as wide as long, slightly wider than mesosoma; lateral ocellus contiguous with inner orbit of compound eye; POL 1.36–1.67× LOL; occipital carina continuous medially, irregularly punctate; central keel absent (Plates 18C, 20C); medial frons punctate rugulose ventrally, irregularly smooth dorsally; ventrolateral frons punctate rugose; frons below median ocellus punctate reticulate; ocellar triangle largely smooth, with scattered punctures; posterior vertex punctate reticulate; gena punctate rugose; length of A3 equal to length of A2.

*Mesosoma*. Cervical pronotal area densely punctate; dorsal pronotal area punctate rugose; lateral pronotal area smooth anteriorly, punctate rugulose posteriorly; netrion punctate rugulose; notaulus contiguously foveolate; middle lobe of mesoscutum densely punctate, becoming denser anteriorly and at posterior end; lateral lobes of mesoscutum densely punctate throughout; mesoscutellum finely punctate throughout; metascutellum transverse (Plates 18D, 20B), posterior margin slightly pointed medially, longitudinally carinate; propodeum continuous medially (Plates 18D, 20B), not divided into two separated lobes, posterior margin narrowly notched medially, each side with several irregular longitudinal carinae medially, otherwise punctate rugulose, covered by dense, recumbent, white setae; upper mesepisternum with a row of fine longitudinal carinae below subalar pit; lower mesepisternum punctate rugulose; mesopleural depression smooth (Plates 18E, 20C); metapleuron longitudinally striate dorsally, punctate rugulose ventrally.

*Legs*. Slender; hind femur weakly swollen, 3.85–4.38× as long as its maximum width; hind tibia without spines over outer surface; hind basitarsus 9.50–12.33× as long as its maximum width.

*Wings*. Apex of fore wing extending as far as mid-length of T4 to mid-length of T5; R 1.47–2.41× as long as r-rs, R1 1.57–2.40× length of R.

*Metasoma*. Posterior margin of transverse sulcus on T2 slightly convex (Plate 20D); sublateral tergal carinae developed on T1–T3; T1 densely longitudinally striate medially, with scattered punctures in interstices anteriorly, rugulose laterally; T2–T3 densely longitudinally striate medially, with delicate punctures in interstices, punctate rugulose laterally; T4–T5 densely finely longitudinally striate throughout, with delicate punctures in interstices; T6 finely punctate dorsally, densely longitudinally striate laterally, with scattered small punctures in interstices; length of T3 0.95–1.15× length of T6; T5 slightly longer than wide; S2–S4 longitudinally striate, with finely punctate rugulose interstices; S5–S6 longitudinally striate, with finely punctate interstices; prominent longitudinal median carina present on S2–S5.

*Male*. Differing from female as follows: body length 5.21–5.41 mm (n=5); mesosoma orange yellow or brown; metasoma variably brown to black; A1 yellow, remainder of antenna brown to dark brown; T1 sparsely longitudinally striate medially, with punctate rugulose interstices anteriorly, punctate rugulose laterally; T6–T7 longitudinally punctate rugulose; T6 slightly longer than wide; length of T6 1.10–1.33× length of T7; T7 subtriangular, apex sharply pointed (Plate 21B, C); length of T7 1.55–1.80× length of S7; S6–S7 longitudinally punctate rugulose.

**Plate 19.**
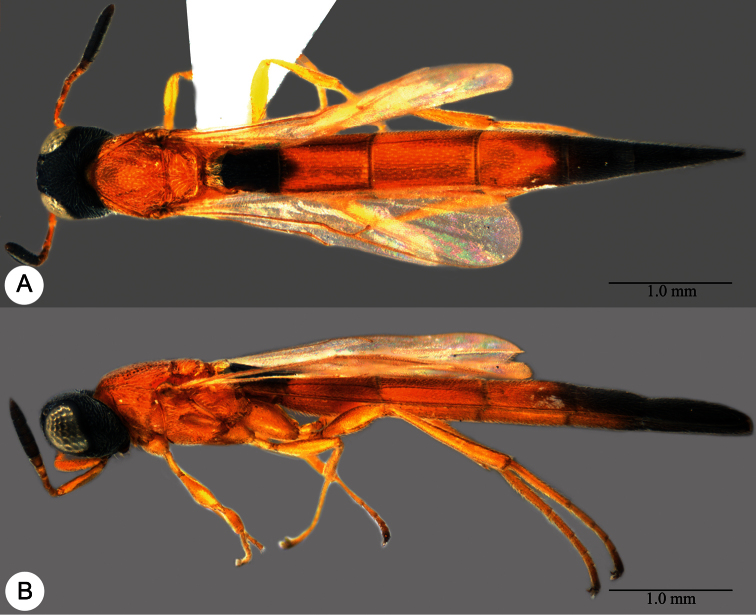
*Macroteleia dolichopa* Sharma, female from Guangdong, Xinfeng, Mt. Yunji. **A** Dorsal habitus **B** Lateral habitus.

**Plate 20.**
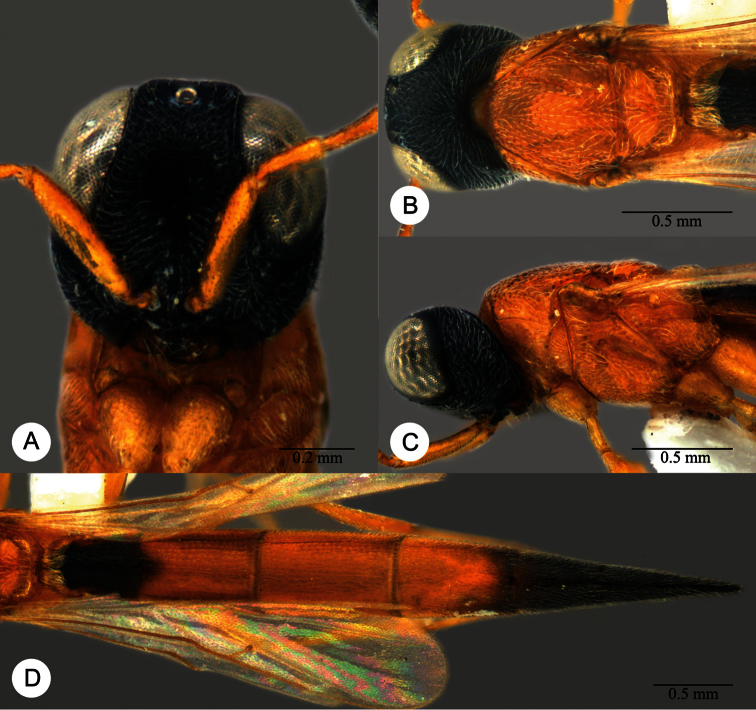
*Macroteleia dolichopa* Sharma, female from Guangdong, Xinfeng, Mt. Yunji. **A** Head, anterior view **B** Head and mesosoma, dorsal view **C** Head and mesosoma, lateral view **D** Metasoma, dorsal view.

**Plate 21.**
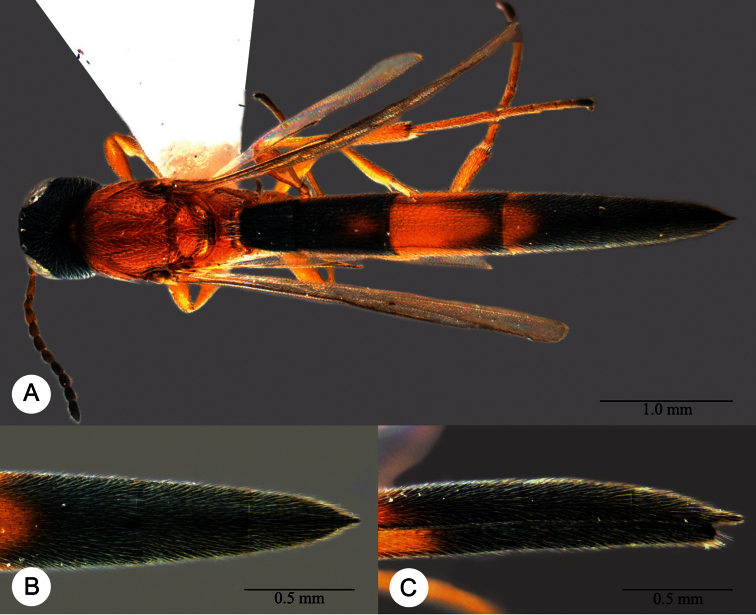
*Macroteleia dolichopa* Sharma, male from Guangdong, Mt. Nankun. **A** Dorsal habitus **B** Apex of metasoma, dorsal view **C** Apex of metasoma, lateral view.

#### Diagnosis.

Themale of this species is similar to that of *Macroteleia striativentris* in body size, but can be separated by the combination of the black head; T7 sharply pointed medially; metapleuron longitudinally striate dorsally, punctate rugulose ventrally.

#### Distribution.

China (Hubei, Guangdong); Vietnam; India. Link to distribution map [http://hol.osu.edu/map-large.html?id=4798].

#### Material examined.

*Holotype*, ♂: **INDIA**: “Vindhya Survey, School of Entomology, St. John’s College, Agra-282002, India, 21.5. Harsa, M.S. Mani & Party, 9–10.IX.1979”, “Holotype”, “*Macroteleia dolichopa* Sharma, S.K., ♀” (deposited in USNM).

Other material. **CHINA**: 1 ♀, Guangdong, Nanling National Nature Reserve, 24°54'N, 113°00'E, 9–18.VII.2004, Juanjuan Ma, SCAU 000035 (SCAU); 1 ♀, Hubei, Huanggang, Mt. Dahu, 31°27'N, 114°32'E, VIII.2009, Chunhong Zheng, SCAU 0000502 (SCAU); 1$, Guangdong, Chebaling National Nature Reserve, 24°43'N, 114°14'E, 23–28.VII.2008, Zaifu Xu, SCAU 000038 (SCAU); 1 ♀, Guangdong, Nanling National Nature Reserve, 24°54'N, 113°00'E, 8–17.VIII.2010, sweeping, Huayan Chen, SCAU 000037 (SCAU); 1 ♀, Guangdong, Nanling National Nature Reserve, 24°54'N, 113°00'E, 16–21.VII.2008, Zaifu Xu, SCAU 000036 (SCAU); 1 ♀ + 1 ♂, Guangdong, Xinfeng, Mt. Yunji, 24°04'N, 114°10'E, 19.VII.2003, Yanxia Song, SCAU 000039, 000040 (SCAU); 3 ♂, Guangdong, Mt. Nankun, 23°37.287'N, 113°51.267'E, 2.VII.2005, Zaifu Xu, SCAU 000041–000043 (SCAU). **VIETNAM**: 1 ♂, “Bac Thai, Phu Luong, Quan Chu, 21.IV.1986, A. Sarkov”, “Paratypus, *Macroteleia fugacious* sp. n.” (IEBR).

### Macroteleia emarginata

Dodd

urn:lsid:zoobank.org:act:76F8C774-CBF3-49EA-BEB5-63E02F148EFF

http://species-id.net/wiki/Macroteleia_emarginata

Plates 22 –24

Macroteleia emarginata Dodd 1920: 326 (original description); Masner, 1965: 82 (type information).

#### Description.

*Female*. Body length 5.48–6.14 mm (n=18).

*Color*. Body black; mandible reddish brown; palpi brown; base of hind coxa blackish, remainder of legs pale brown, becoming darker distally; A1 brown, A2–A5 dark brown, remainder of antenna black; fore wing hyaline.

*Head*. Transverse in dorsal view, 1.30–1.50× as wide as long, slightly wider than mesosoma; OOL short, 0.10–0.31× times minimum diameter of lateral ocellus; POL 1.29–1.64× LOL; occipital carina continuous medially, irregularly punctate; central keel absent (Plate 23A); medial frons obliquely strigose ventrally, irregularly smooth dorsally; ventrolateral frons punctate rugose; frons below median ocellus densely punctate, with punctures in part contiguous; ocellar triangle largely smooth, with scattered punctures; posterior vertex densely punctate, with punctures in part contiguous; gena coarsely punctate rugose; length of A3 equal to length of A2.

*Mesosoma*. Cervical pronotal area densely punctate; dorsal pronotal area densely coarsely punctate; lateral pronotal area smooth anteriorly, punctate rugulose ventrally; netrion densely finely punctate; notaulus deep, distinctly foveolate; middle lobe of mesoscutum densely punctate anteriorly and at posterior end, usually sparsely punctate on a small area across middle; lateral lobe of mesoscutum densely punctate throughout; mesoscutellum densely punctate throughout; metascutellum transverse (Plate 23B), posterior margin slightly pointed medially, longitudinally carinate; propodeum continuous medially (Plate 23B), not divided into two separated lobes, posterior margin narrowly notched medially, each side with several irregular longitudinal carinae medially, otherwise punctate rugulose, covered by dense, recumbent, white setae; upper mesepisternum with a row of robust longitudinal carinae below subalar pit; lower mesepisternum variably smooth to punctate rugulose; mesopleural depression smooth (Plate 23C); metapleuron longitudinally striate dorsally, punctate rugulose ventrally.

*Legs*. Slender; hind femur weakly swollen, 3.68–4.76× as long as its maximum width; hind tibia without spines over outer surface; hind basitarsus 9.44–12.33× as long as its maximum width.

*Wings*. Apex of fore wing extending from as far as posterior fourth of T4 to anterior third of T5; R 1.41–1.94× as long as r-rs, R1 1.73–2.26× length of R.

*Metasoma*. Posterior margin of transverse sulcus on T2 slightly to strongly convex (Plate 23D); sublateral tergal carinae developed on T1–T3; T1 densely longitudinally striate, with punctate rugulose sculpture in interstices anteriorly, punctate rugulose laterally; T2–T3 densely longitudinally striate medially, with delicate punctures in interstices, punctate rugulose laterally; T4–T5 densely longitudinally striate throughout, with delicate punctures in interstices; T6 punctate rugulose dorsally, densely longitudinally striate laterodorsally, with scattered small punctures in interstices; length of T3 0.93–1.03× length of T6; T5 longer than wide or slightly wider than long; S2–S4 sparsely and longitudinally striate, with delicate punctures in interstices; S5–S6 densely longitudinally striate, with delicate punctures in interstices; prominent longitudinal median carina present on S2–S5.

*Male*. Differing from female as follows: body length 4.08–6.26 mm (n=20); A1 brown, remainder of antenna dark brown; T4–T6 densely longitudinally striate throughout, with numerous delicate punctures in interstices; T7 punctate rugulose throughout; T6 slightly longer than wide; length of T6 1.42–1.84× length of T7; T7 subtriangular, apex pointed (Plate 24B, C); length of T7 1.43–1.87× length of S7; S7 longitudinally punctate rugulose; prominent longitudinal median carina present on S2–S6.

**Plate 22.**
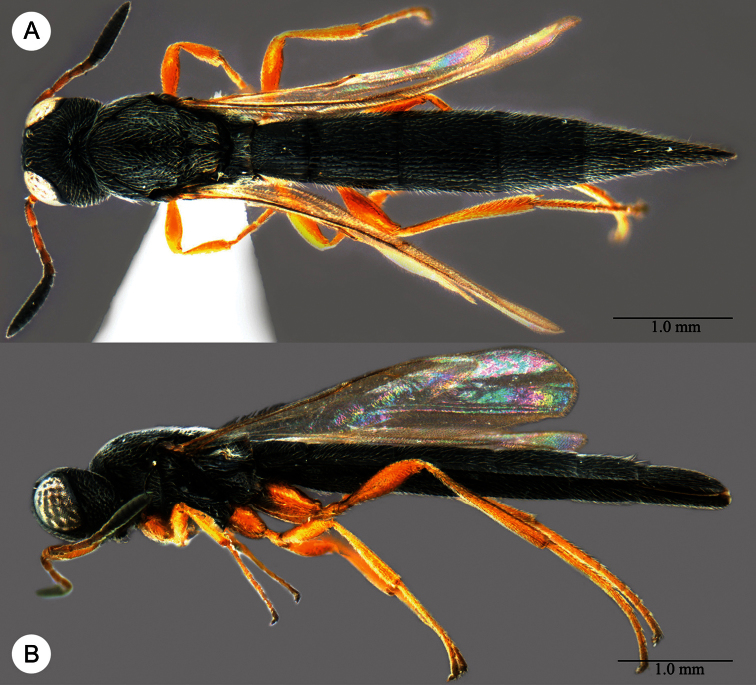
*Macroteleia emarginata* Dodd, female from Guangdong, Mt. Nankun. **A** Dorsal habitus **B** Lateral habitus.

**Plate 23.**
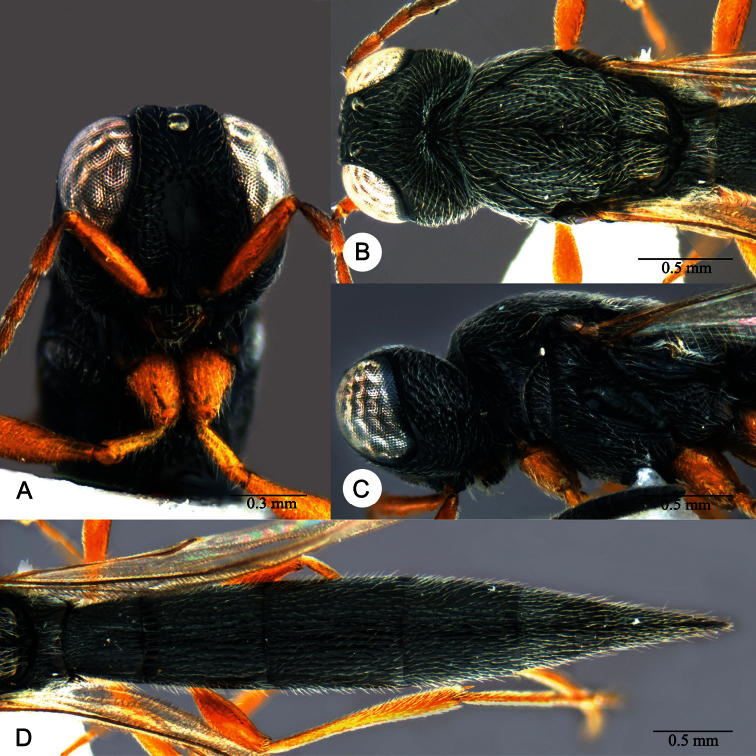
*Macroteleia emarginata* Dodd, female from Guangdong, Mt. Nankun. **A** Head, anterior view **B** Head and mesosoma, dorsal view **C** Head and mesosoma, lateral view **D** Head, anterior view **E** Metasoma, dorsal view.

**Plate 24.**
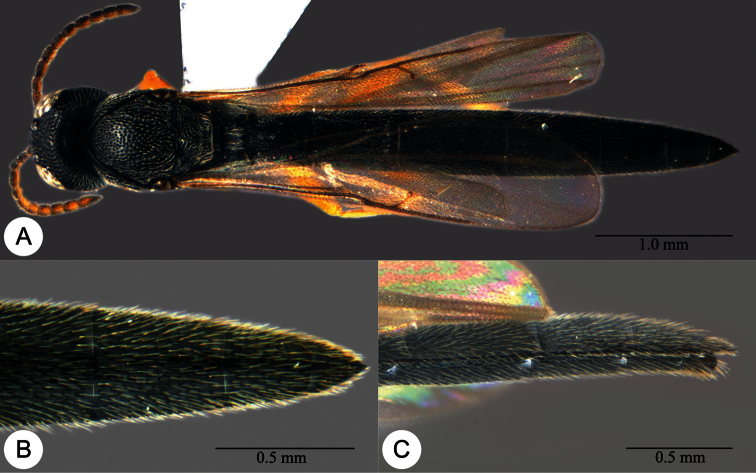
*Macroteleia emarginata* Dodd, male from Hunan, Mangshan Nature Reserve. **A** Dorsal habitus **B** Apex of metasoma, dorsal view **C** Apex of metasoma, lateral view.

#### Distribution.

China (Fujian, Hunan, Guangdong, Hainan, Guizhou, Yunnan); Malaysia. Link to distribution map [http://hol.osu.edu/map-large.html?id=4802].

#### Material Examined.

Other material. **CHINA**: 1 ♀, Zhejiang, Mt. Fengyang, 27°56'N, 119°12'E, 18.VIII.2003, Wuqing Fang, SCAU 000324 (SCAU); 1 ♂, Fujian, Huangzhulin Nature Reserve, 26°12'N, 118°51'E, 5.VII.2005, Jingxian Liu, SCAU 000325 (SCAU); 1 ♂, Hunan, Nanyue, Mt. Heng, 27°15'N, 112°43'E, 4.IX.1980, Xinwang Tong, SCAU 000326 (SCAU); 1 ♀, Hunan, Nanyue, Mt. Heng, 27°15'N, 112°43'E, 5.IX.1980, Xinwang Tong, SCAU 000327 (SCAU); 2 ♀ + 14 ♂, Hunan, Mangshan Nature Reserve 24°56'N, 112°69'E, 13.VIII.2010, sweeping, Huayan Chen, SCAU 000328–000343 (SCAU); 1 ♀ + 1 ♂, Guangdong, Chebaling National Nature Reserve, 24°43'N, 114°14'E, 23–28.VII.2008, Zaifu Xu, SCAU 000344, 000345 (SCAU); 1 ♀, Guangdong, Nanling National Nature Reserve, 24°54'N, 113°00'E, 5.VI.2010, Huayan Chen, SCAU 000346 (SCAU); 1 ♂, Guangdong, Nanling National Nature Reserve, 24°54'N, 113°00'E, 9–18.VII.2004, Juanjuan Ma, SCAU 000347 (SCAU); 1 ♀, Guangdong, Mt. Nankun, 23°37.941'N, 113°50.182'E, 12.V.2004, Zaifu Xu, SCAU 000348 (SCAU); 1 ♀, Guangdong, Mt. Nankun, 23°37.941'N, 113°50.182'E, 2–3.VII.2005, Zaifu Xu, SCAU 000349 (SCAU); 3 ♀, Guangdong, Mt. Nankun, 23°37.941'N, 113°50.182'E, 15.VI.2007, Zaifu Xu, SCAU 000350–000352 (SCAU); 1 ♀, Guangdong, Mt. Nankun, 23°37.941'N, 113°50.182'E, 6.V.2010, Jingxian Liu, SCAU 000353 (SCAU); 1 ♀, Guangdong, Mt. Nankun, 23°37.941'N, 113°50.182'E, 23.V.2010, Zaifu Xu, SCAU 000354 (SCAU); 2 ♀, Guangdong, Mt. Nankun, 23°37.941'N, 113°50.182'E, 540m, 4.VI.2011, yellow pan trap, Huayan Chen, SCAU 000355, 000356 (SCAU); 1 ♀, Guangdong, Guangzhou, Liuxihe, 23°44'N, 113°47'E, 29.VIII.2004, Zaifu Xu, SCAU 000357 (SCAU); 1 ♂, Hainan, Mt. Diaoluo, 18°39'N, 109°53'E, 1–2.VI.2007, Jingxian Liu, SCAU 000358 (SCAU); 2 ♀, Guizhou, Guiyang Forest Park, 26°33'N, 106°44'E, 23.IX.2007, Cuihong Xie, SCAU 000359, 000360 (SCAU); 1 ♂, Guizhou, Leishan County, Fangxiang Town, 26°25'N, 108°16'E, 2.VI.2005, Jingxian Liu, SCAU 000361 (SCAU); 1 ♀, Guizhou, Leigongshan National Nature Reserve, Xiaodanjiang, 25°53'N, 108°24'E, 150 m, 4.VI.2005, Jingxian Liu, SCAU 000362 (SCAU); 1 ♂, Guizhou, Leigongshan National Nature Reserve, Xiaodanjiang, 25°53'N, 108°24'E, 150M, 4.VI.2005, Hongying Zhang, SCAU 000363 (SCAU); 2 ♂, Guizhou, Dashahe Nature Reserve, 29°00'N, 107°36'E, 18.VIII.2004, Qiong Wu, SCAU 000364, 000365 (SCAU); 1 ♂, Yunnan, Nabanhe Basin National Nature Reserve, 22°15.474'N, 100°36.322'E, 892M, 19–23.VII.2011, yellow pan trap, Zaifu Xu, SCAU 000366 (SCAU).

#### Comments.

We did not examine the holotype of this species. The identification is based upon the careful description provided by Alan Dodd in the original publication.

### Macroteleia flava sp. n.

urn:lsid:zoobank.org:act:8594A627-E6B3-4740-908D-676EFF342C08

http://species-id.net/wiki/Macroteleia_flava

Plates 25 –26

#### Description.

*Female*. Body length 4.35–5.76 mm (n=5).

*Color*. Head and mesosoma orange yellow; base of T1 and T6 variably dark brown to black, otherwise orange yellow; mandible dark brown; palpi yellow; legs yellow throughout; A1–A6 brown, A7–A12 black; fore wing hyaline.

**Plate 25.**
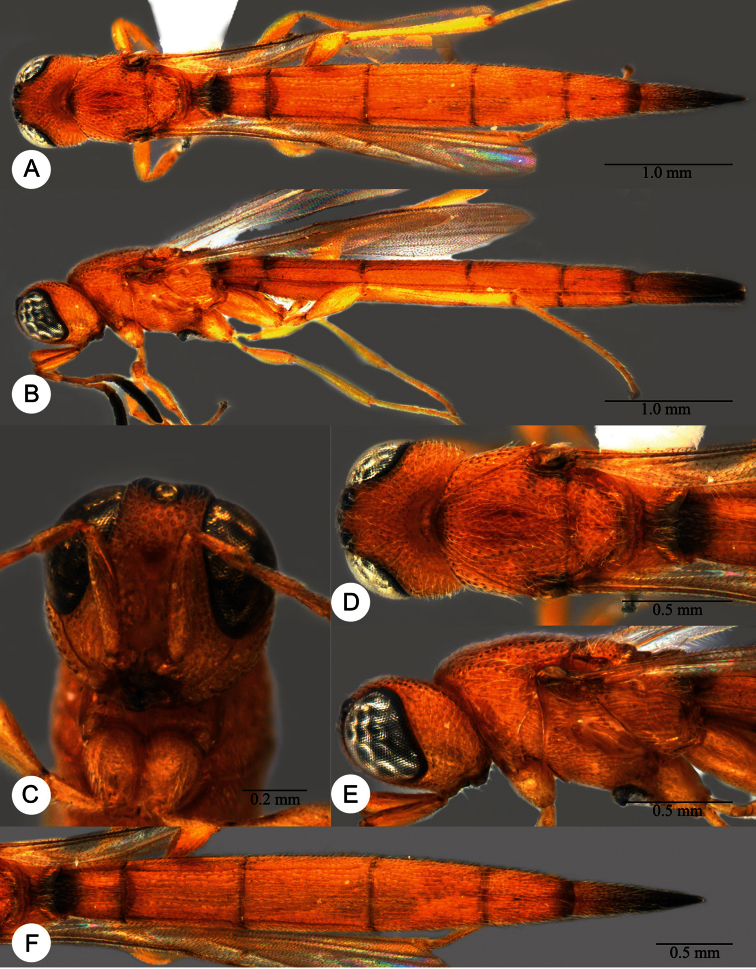
*Macroteleia flava* sp. n., holotype, female. **A** Dorsal habitus **B** Lateral habitus. **C** Head, anterior view **D** Head and mesosoma, dorsal view **E** Head and mesosoma, lateral view **F** Metasoma, dorsal view.

*Head*. Transverse in dorsal view, 1.40–1.44× as wide as long, slightly wider than mesosoma; OOL short, 0.14–0.31× minimum ocellar width; POL 1.20–1.31× LOL; occipital carina weakly continuous or interrupted medially; central keel absent (Plate 25C); medial frons punctate rugulose ventrally, irregularly smooth dorsally; ventrolateral frons punctate rugose; frons below median ocellus densely punctate; vertex densely punctate, with punctures in part contiguous; gena punctate rugose; length of A3 equal to length of A2.

*Mesosoma*. Cervical pronotal area densely punctate; dorsal pronotal area punctate rugose; lateral pronotal area smooth anteriorly, punctate rugulose posteriorly; netrion punctate rugulose; notaulus narrow, but distinctly foveolate; middle lobe of mesoscutum densely punctate, becoming denser anteriorly and at posterior end; lateral lobes of mesoscutum densely punctate throughout; mesoscutellum densely punctate throughout; metascutellum distinctly transverse (Plate 25D), posterior margin slightly pointed medially, longitudinally carinate; propodeum continuous medially (Plate 25D), not divided into two separated lobes, posterior margin narrowly notched medially, each side with several irregular longitudinal carinae medially, otherwise punctate rugulose, covered by dense, recumbent, white setae; upper mesepisternum with a row of fine longitudinal carinae below subalar pit; lower mesepisternum punctate rugulose; mesopleural depression smooth (Plate 25E); metapleuron longitudinally striate throughout.

*Legs*. Slender; hind femur weakly swollen, 3.60–4.39× as long as its maximum width; hind tibia without spines over outer surface; hind basitarsus 11.66–12.83× as long as its maximum width.

*Wings*. Apex of fore wing extending from as far as posterior third to posterior margin of T4; R 1.76–2.25× as long as r-rs, R1 1.39–2.00× length of R.

*Metasoma*. Posterior margin of transverse sulcus on T2 strongly convex (Plate 25F); sublateral tergal carinae developed on T1–T3; T1 densely longitudinally striate medially, with scattered punctures in interstices anteriorly, rugulose laterally; T2–T3 densely longitudinally striate medially, with delicate punctures in interstices, punctate rugulose laterally; T4–T5 densely finely longitudinally striate throughout, with delicate punctures in interstices; T6 finely punctate dorsally, densely longitudinally striate laterally, with scattered small punctures in interstices; length of T3 0.83–0.99× length of T6; T5 distinctly longer than wide; S2–S4 longitudinally striate, with finely punctate rugulose interstices; S5–S6 longitudinally striate, with finely punctate interstices; prominent longitudinal median carinae present on S2–S5.

**Plate 26.**
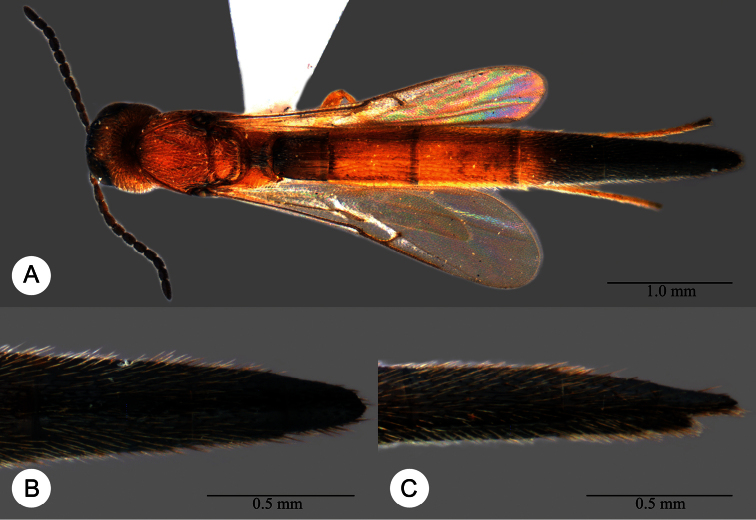
*Macroteleia flava* sp. n., paratype, male. **A** Dorsal habitus **B** Apex of metasoma, dorsal view **C** Apex of metasoma, lateral view.

*Male*. Differing from female as follows: body length 4.55–5.26 mm (n=6); head brown; mesosoma darker than female; metasoma variably brown to black; A1 yellow, remainder of antenna brown to dark brown; T1–T3 densely longitudinally striate medially, with delicate punctures in interstices, rugulose laterally; T4–T7 longitudinally punctate rugulose; T6 slightly longer than wide; length of T6 1.09–1.27× length of T7; T7 subtriangular, apex pointed (Plate 26B, 26C); length of T7 1.75–2.29× length of S7; S2–S5 longitudinally striate, with delicate punctures in interstices; S6–S7 longitudinally punctate rugulose.

#### Diagnosis.

The body shape, color and size of *Macroteleia flava* is similar to *Macroteleia rufa* and *Macroteleia chandelii*. It differs from them in that metascutellum is distinctly transverse (triangular in *Macroteleia rufa*, tongue-like in *Macroteleia chandelii*), and the propodeum is continuous medially, not divided into two separated lobes (divided into two subtriangular lobes in the latter two species).

#### Etymology.

The name *flava* refers to orange yellow body color of this species and is used as an adjective.

#### Distribution.

China (Hebei, Hunan, Guangdong); Thailand. Link to distribution map [http://hol.osu.edu/map-large.html?id=320504].

#### Material examined.

*Holotype*, ♀: **CHINA**: Hebei, Mt. Dongling, 40°02'N, 115°27'E, 11.VIII.2005, Ming Shi, SCAU 000078 (deposited in SCAU). *Paratypes*: 1 ♂, Hunan, Nanyue, Mt. Heng, 27°15'N, 112°43'E, 20.VI.1980, Xinwang Tong, SCAU 000079 (SCAU);1 ♂, Hunan, Nanyue, Mt. Heng, 27°15'N, 112°43'E, 17.VIII.1980, Xinwang Tong, SCAU 000080 (SCAU); 1 ♀, Hunan, Nanyue, Mt. Heng, 27°15'N, 112°43'E, 4.IX.1980, Xinwang Tong, SCAU 000081 (SCAU); 1 ♂, Hunan, Nanyue Town, 27°14'N, 112°44'E, 8.IX.1980, Xinwang Tong, SCAU 000082 (SCAU); 1 ♂, Hunan, Liuyang City, 28°09'N, 113°38'E, 17.VIII.1985, Xinwang Tong, SCAU 000083 (SCAU); 1 ♀, Guangdong, Meizhou, Fenxi Forestry Farm, 24°38'N, 116°47'E, 28.VII.2003, Jianuan Zhou, SCAU 000084 (SCAU); 1 ♂, Guangdong, Xinfeng, Mt. Yunji, 24°04'N, 114°10'E, 19.VII.2003, Yanxia Song, SCAU 000496 (SCAU); 1 ♂, Guangdong, Mt. Nankun, 23°37.941'N, 113°50.182'E, 2.VII.2005, Zaifu Xu, SCAU 000085 (SCAU). **THAILAND**: 1 ♀, Chiang Mai: Maerim, 11.XI.2002, MT, R. A. Beaver, No. 25682 (RABC); 1 ♀, Chiang Mai: Maerim, 5–8.IV.2003, MT, R. A. Beaver, No. 27038 (RABC).

### Macroteleia gracilis sp. n.

urn:lsid:zoobank.org:act:FC1AC5B9-9F13-4AC7-9057-7DD106F227AB

http://species-id.net/wiki/Macroteleia_gracilis

Plates 27 –28

#### Description.

*Male*. Body length 4.76–6.05 mm (n=4).

*Color*. Body black; mandible reddish brown; palpi yellow; hind coxa blackish, tarsi yellow, remainder of legs pale brown; A1 brown, remainder of antenna dark brown; fore wing hyaline.

*Head*. Transverse in dorsal view, 1.44–1.48× as wide as long, slightly wider than mesosoma; OOL short, 0.17–0.29× minimum ocellar width; POL 1.54–1.57× LOL; occipital carina weakly continuous medially, irregular punctate; central keel weakly developed, extending onto interantennal process (Plate 28A); medial frons punctate rugulose ventrally, with irregularly shaped smooth area dorsally; frons below median ocellus sparsely punctate medially, densely punctate laterally; vertex punctate rugulose; gena punctate rugose; length of A3 1.25–1.31× length of A2.

*Mesosoma*. Cervical pronotal area densely punctate; dorsal pronotal area punctate rugulose; lateral pronotal area smooth dorsally, punctate rugulose ventrally; netrion finely punctate rugulose; notaulus shallow, foveolate; middle lobe of mesoscutum densely punctate, sculpture becoming denser anteriorly; lateral lobes of mesoscutum densely finely punctate throughout; mesoscutellum densely finely punctate throughout; metascutellum transverse (Plate 28B), posterior margin slightly pointed medially, longitudinally carinate; propodeum continuous medially (Plate 28B), not divided into two separated lobes, posterior margin narrowly notched medially, each side with several irregular longitudinal carinae medially, otherwise punctate rugulose, covered by dense, recumbent, white setae; upper mesepisternum with a row of somewhat robust longitudinal carinae below subalar pit; lower mesepisternum variably smooth to punctate rugulose; mesopleural depression smooth (Plate 28C); metapleuron longitudinally striate throughout.

*Legs*. Slender; hind femur weakly swollen, 4.23–4.80× as long as its maximum width; hind tibia without spines over outer surface; hind basitarsus 12.60–14.00× as long as its maximum width.

*Wings*. Apex of fore wing extending from as far as posterior fourth of T3 to base of T4; R 2.06–2.46× as long as r-rs, R1 1.63–1.90× length of R.

*Metasoma*. Posterior margin of transverse sulcus on T2 slightly convex (Plate 28D); sublateral tergal carinae developed on T1–T3; T1–T2 sparsely and longitudinally striate medially, with delicate punctures in interstices, punctate rugulose laterally; T3–T6 densely longitudinally striate, with numerous delicate punctures in interstices; T7 punctate rugulose throughout, with longitudinal tendency; T6 distinctly longer than wide; length of T6 0.93–1.18× length of T7; T7 subtriangular, apex pointed (Plate 28E, 28F); length of T7 2.50–3.22× length of S7; S2–S3 sparsely longitudinally striate, with numerous delicate punctures and irregular microsculptures in interstices; S4–S7 densely longitudinally striate, with numerous delicate punctures in interstices; prominent longitudinal median carina present on S2–S5.

*Female*. Unknown.

**Plate 27.**
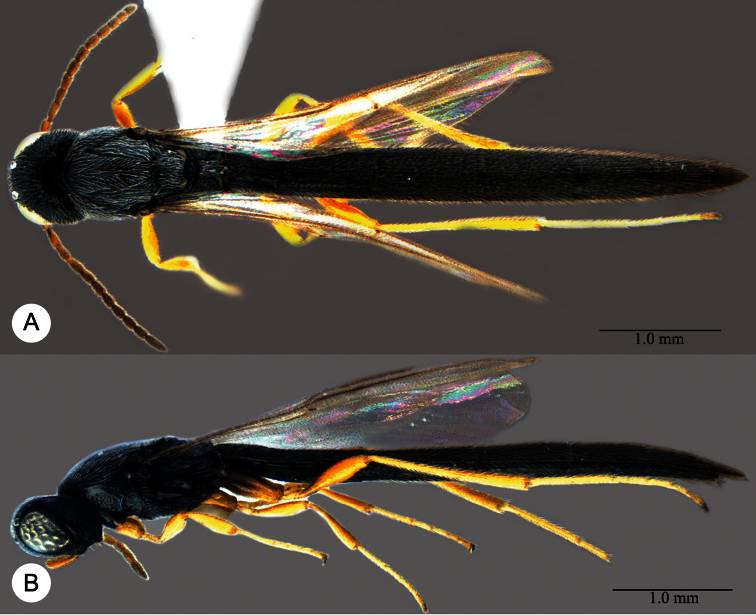
*Macroteleia gracilis* sp. n., holotype, male. **A** Dorsal habitus **B** Lateral habitus.

**Plate 28.**
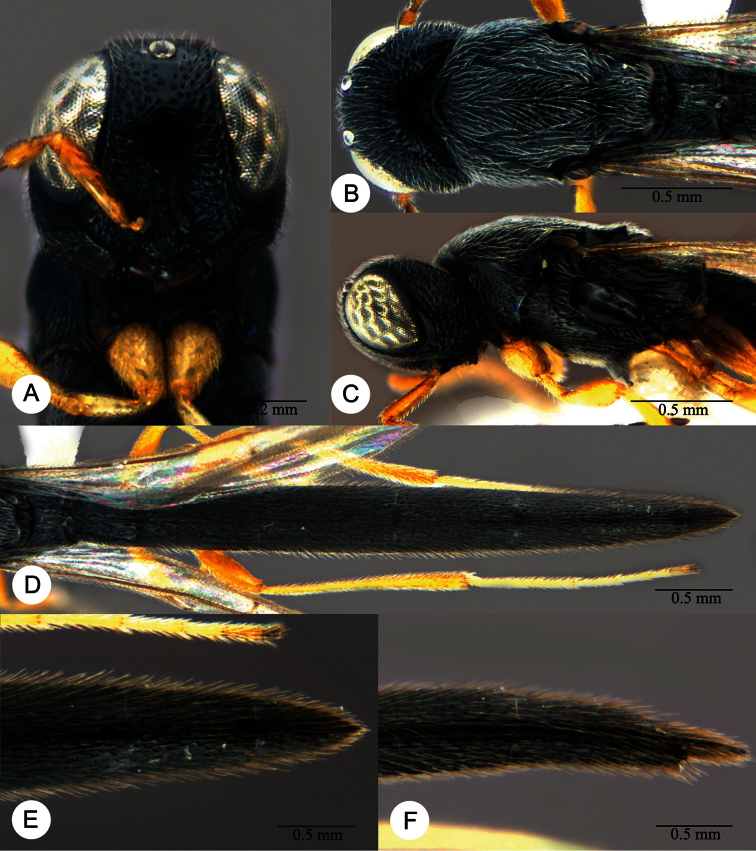
*Macroteleia gracilis* sp. n., holotype, male. **A** Head, anterior view **B** Head and mesosoma, dorsal view **C** Head and mesosoma, lateral view **D** Metasoma, dorsal view **E** Apex of metasoma, dorsal view **F** Apex of metasoma, lateral view.

#### Diagnosis.

The male of this species is similar to that of *Macroteleia emarginata* in size and color, but can be distinguished in having the metapleuron longitudinally striate throughout (*Macroteleia emarginata* longitudinally striate dorsally, punctate rugulose ventrally); and the length of T7 2.50–3.22× length of S7 (distinctly shorter, 1.42–1.87× in *Macroteleia emarginata*).

#### Etymology.

The name *gracilis* refers to the slender body of this species and is used as an adjective.

#### Distribution.

China (Guangdong). Link to distribution map [http://hol.osu.edu/map-large.html?id=320505].

#### Material examined.

*Holotype*, ♂: **CHINA**: Guangdong, Mt. Nankun, 23°37.941'N, 113°50.182'E, 1.VI.2009, Huayan Chen, SCAU 000053 (deposited in SCAU). *Paratypes*: 2 ♂, Guangdong, Mt. Nankun, 23°37.941'N, 113°50.182'E, 540 m, 4.VI.2011, yellow pan trap, Huayan Chen, SCAU 000054, 000055 (SCAU); 1 ♂, Guangdong, Shimen National Forest Park, 23°37.287'N, 113°51.267'E, 4.VI.2009, Huayan Chen, SCAU 000056 (SCAU).

### Macroteleia indica

Saraswat & Sharma

http://species-id.net/wiki/Macroteleia_indica

Plates 29 –35

Macroteleia indica Saraswat and Sharma 1978: 11 (original description); Mani and Sharma 1982: 171 (description); Saraswat 1982: 346 (description of male); Mukerjee 1994: 4 (color variation).
Macroteleia cebes Lê 2000: 52, 54, 331 (original description, keyed), **syn. n.**
Macroteleia dones Lê 2000: 52, 53, 57, 333 (original description, keyed), **syn. n.**
Macroteleia domes Lê 2000: 52 (keyed, misspelling).

#### Description.

*Female*. Body length 3.13–4.76 mm (n=20).

*Color*. Head yellow or orange throughout, or dark orange, becoming darker dorsally; mesosoma yellow or orange, becoming darker dorsally; base of T1, T5 and T6 brown to black, remainder of metasoma yellow or orange; mandible yellow with teeth black; palpi yellow; legs yellow throughout; A1 yellow, A2–A6 yellow or brown, remainder of antenna black; fore wing hyaline.

*Head*. Transverse in dorsal view, 1.26–1.40× as wide as long, slightly wider than mesosoma; OOL short, 0.10–0.40× times minimum diameter of lateral ocellus; POL 1.14–1.36× LOL; occipital carina interrupted medially; central keel absent (Plate 34A); medial frons obliquely strigose ventrally, irregularly smooth dorsally; ventrolateral frons punctate rugose; frons below median ocellus densely punctate, punctures in part contiguous; vertex densely punctate, interspaces in part with microsculpure; gena coarsely punctate rugose; length of A3 0.80–0.94× length of A2.

*Mesosoma*. Cervical pronotal area densely punctate; dorsal pronotal area densely coarsely punctate; lateral pronotal area smooth on upper anterior angle, punctate rugose posteriorly; netrion punctate rugulose; notaulus shallow, irregularly foveolate; middle lobe of mesoscutum moderately punctate; lateral lobes of mesoscutum densely punctate; mesoscutellum densely punctate throughout; metascutellum transverse (Plates 31D, 32C, 34B), posterior margin slightly pointed medially, longitudinally carinate; propodeum continuous medially (Plates 31D, 32C, 34B), not divided into two separated lobes, posterior margin narrowly notched medially, each side with several irregular longitudinal carinae submedially, otherwise punctate rugulose, covered by dense, recumbent, white setae; upper mesepisternum with a row of weak longitudinal carinae or a ledge below subalar pit; lower mesepisternum punctate rugulose; mesopleural depression smooth (Plates 31E, 32D, 34C); metapleuron longitudinally striate throughout.

*Legs*. Slender; hind femur weakly swollen, 3.75–4.33× as long as its maximum width; hind tibia without spines over outer surface; hind basitarsus 10.00–13.43× as long as its maximum width.

*Wings*. Apex of fore wing extending as far as posterior margin of T4 to posterior margin of T5; R 1.70–2.25× as long as r-rs, R1 1.57–2.05× length of R.

*Metasoma*. Posterior margin of transverse sulcus on T2 slightly convex (Plates 31F, 34D); sublateral tergal carinae developed on T1–T4; T1–T4 densely longitudinally striate medially, with scattered delicate punctures in interstices, punctate rugulose laterally; T5 densely longitudinally striate throughout, with delicate punctures in interstices; T6 finely punctate dorsally, densely longitudinally striate laterally, with scattered small punctures in interstices; length of T3 1.11–1.39× length of T6; T5 distinctly wider than long; S2–S3 moderately longitudinally striate, with punctate rugulose sculpture in interstices; S4–S6 densely longitudinally striate, with delicate punctures in interstices; prominent longitudinal median carina present on S2–S5.

*Male*. Differing from female as follows: body length 2.94–4.17 mm (n=20); body darker than female; A1 pale brown, remainder of antenna brown to dark brown, becoming darker apically; T5–T6 densely longitudinally striate, with numerous delicate punctures in interstices; T7 punctate rugulose throughout; T6 distinctly wider than long; length of T6 1.82–2.57× length of T7; T7 transverse, apex truncate (Plate 35B); length of T7 0.73–0.91× length of S7; S6–S7 longitudinally punctate rugulose; prominent longitudinal median carina present on S2–S6.

**Plate 29.**
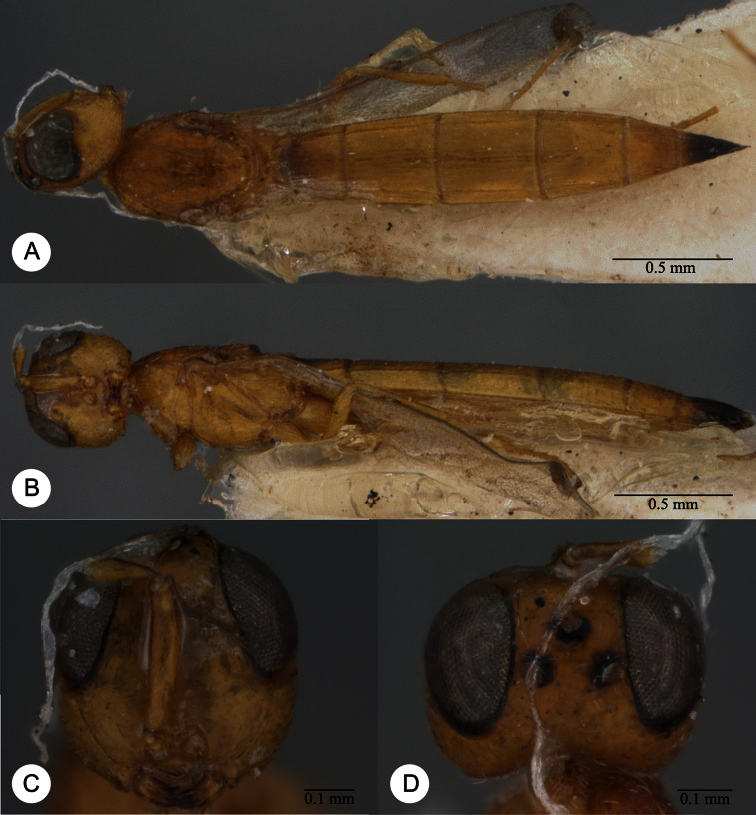
*Macroteleia indica* Saraswat & Sharma, holotype, female. **A** Dorsal habitus **B** Lateral habitus **C** Head, anterior view **D** Head, dorsal view.

**Plate 30.**
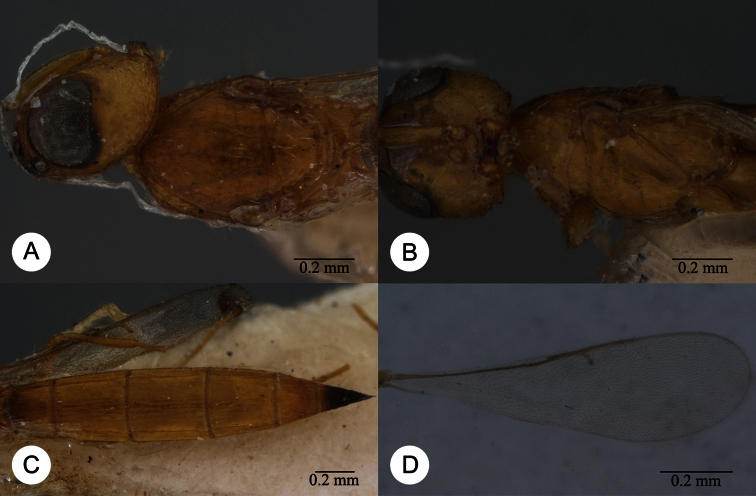
*Macroteleia indica* Saraswat & Sharma, holotype, female. **A** Head and mesosoma, dorsal view **B** Head and mesosoma, lateral view **C** Metasoma, dorsal view **D** Fore wing.

**Plate 31.**
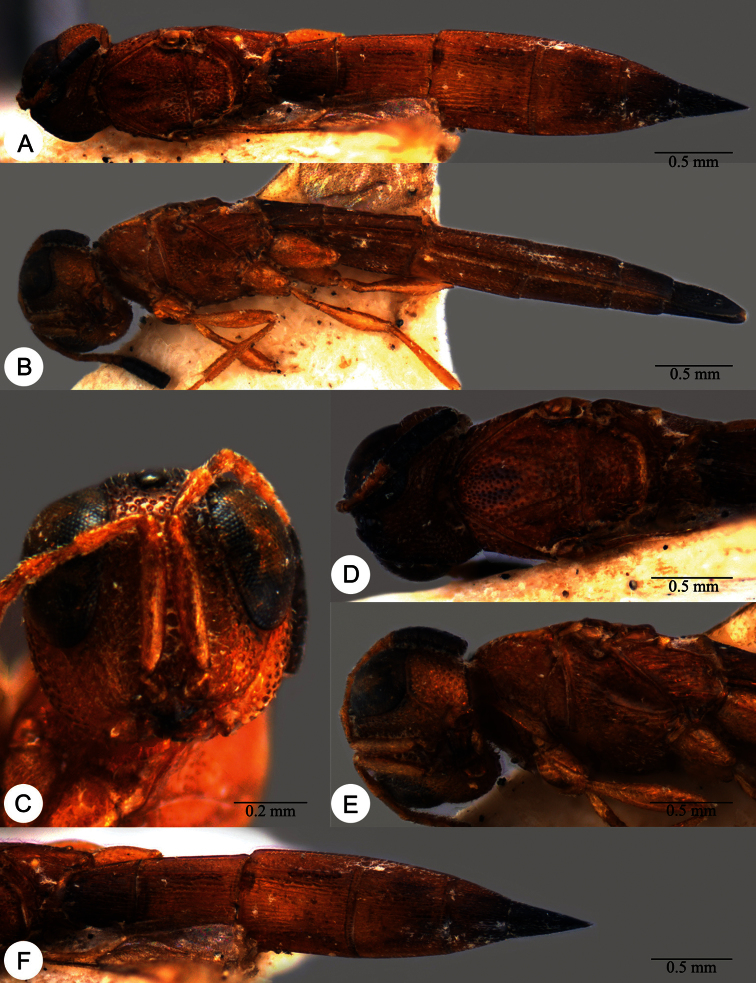
*Macroteleia cebes* Kozlov & Lê, holotype, female. **A** Dorsal habitus **B** Lateral habitus **C** Head, anterior view **D** Head and mesosoma, dorsal view **E** Head and mesosoma, lateral view **F** Metasoma, dorsal view.

**Plate 32.**
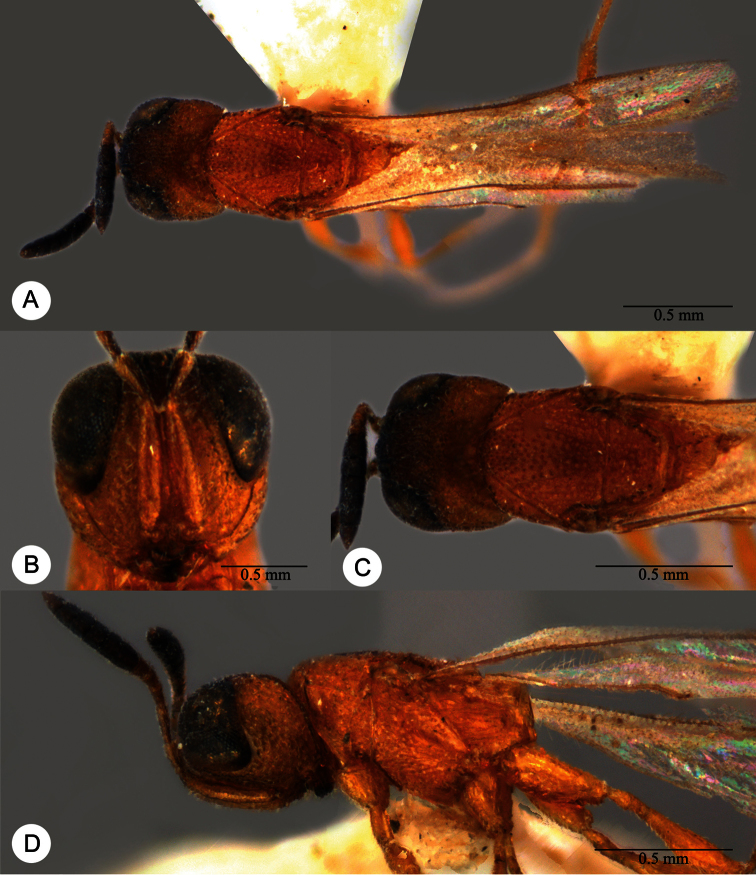
*Macroteleia dones* Kozlov & Lê, holotype, female. **A** Dorsal habitus **B** Head, anterior view **C** Head and mesosoma, dorsal view **D** Head and mesosoma, lateral view.

**Plate 33.**
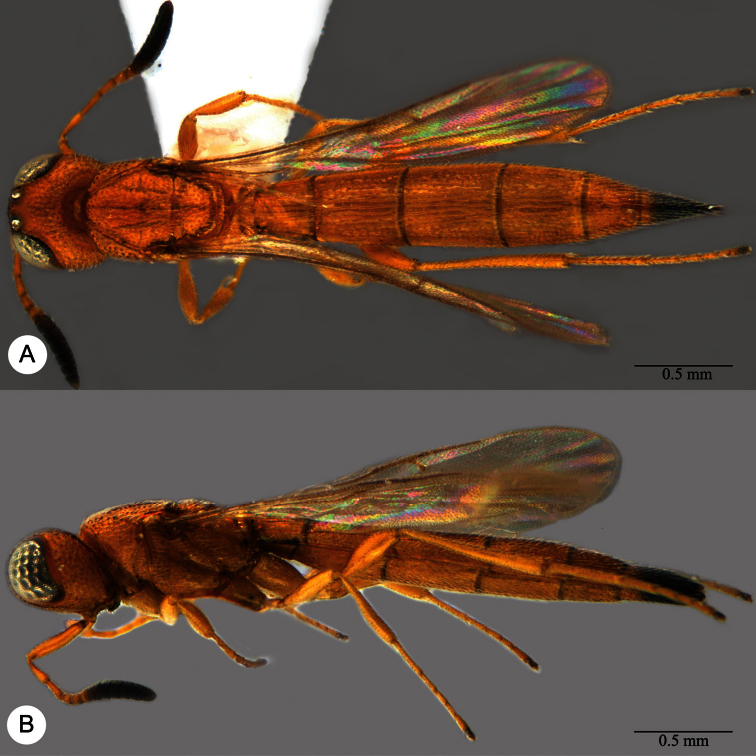
*Macroteleia indica* Saraswat & Sharma, female from Hainan, Baisha, Mt. Jiujialing. **A** Dorsal habitus **B** Lateral habitus.

**Plate 34.**
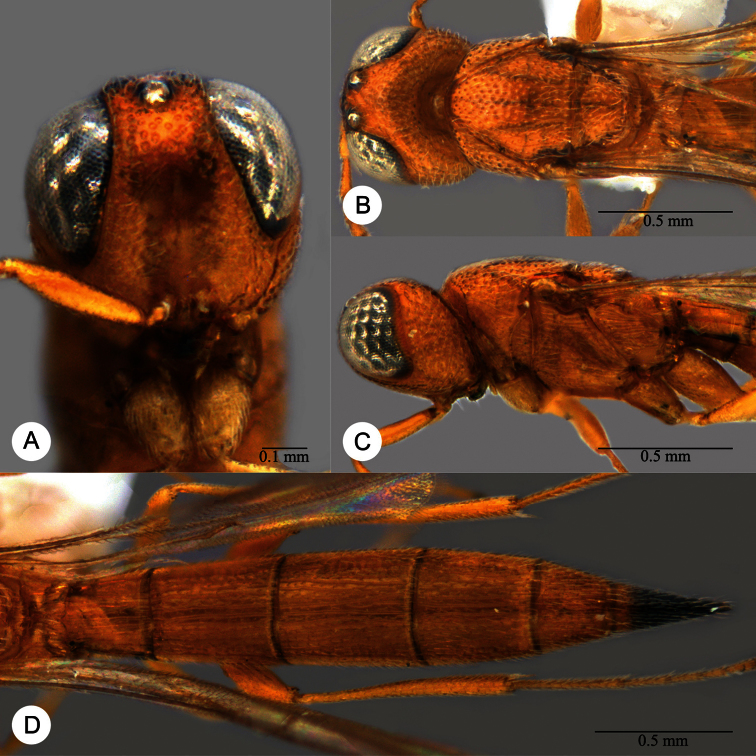
*Macroteleia indica* Saraswat & Sharma, female from Hainan, Baisha, Mt. Jiujialing. **A** Head, anterior view **B** Head and mesosoma, dorsal view **C** Head and mesosoma, lateral view **D** Metasoma, dorsal view.

**Plate 35.**
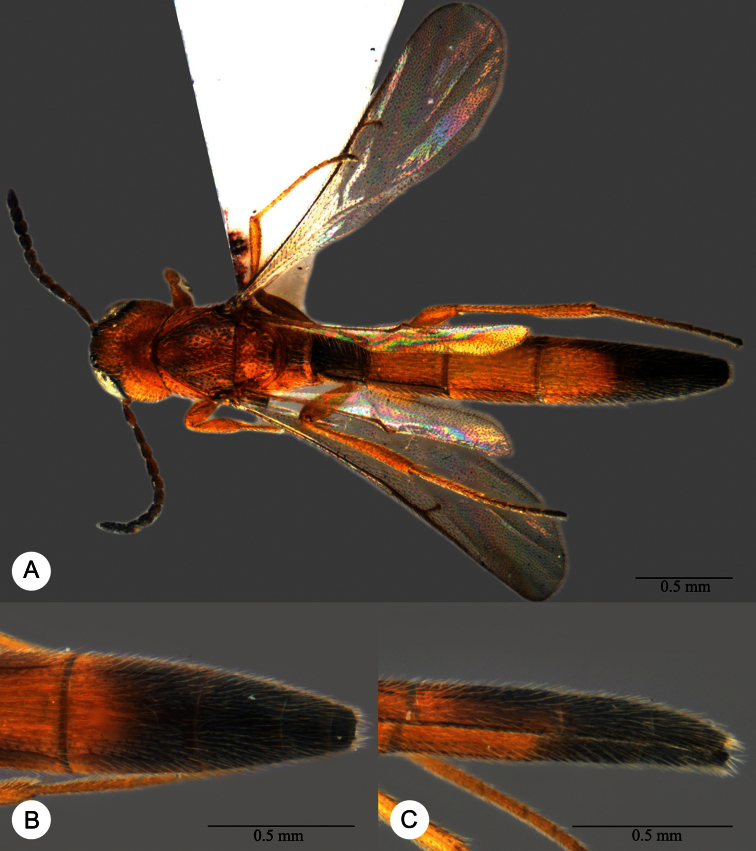
*Macroteleia indica* Saraswat & Sharma, male from Hainan, Baisha, Mt. Jiujialing. **A** Dorsal habitus **B** Apex of metasoma, dorsal view **C** Apex of metasoma, lateral view.

#### Diagnosis.

This species is similar to *Macroteleia flava* in body shape and color, but can be distinguished by its smaller size and its T5 distinctly wider than long (T5 distinctly longer than wide in *Macroteleia flava*).

#### Distribution.

China (Zhejiang, Taiwan, Fujian, Hunan, Guangdong, Hainan, Guangxi, Yunnan); India; Vietnam. Link to distribution map [http://hol.osu.edu/map-large.html?id=4819].

#### Material examined.

*Holotype* of *Macroteleia indica*, ♀: **INDIA**: “North-Bengal Survey, School of Entomology, St. John’s College, Agra-282002, India, 16.1. Alipur Duar, M.S. Mani & Party, 1–19.IV.1976”, “Holotype”, “*Macroteleia indica* Sharma, NO. SA+1, ♀” (deposited in USNM). *Holotype* of *Macroteleia cebes*, ♀, **VIETNAM**: “Thuong Tien, HSB [=Hoa Binh], rice field, 8.XI.1978, Lê Xuan Hue”, “Holotypus ♀ *Macroteleia cebes* Kozlov et Lê 84” (deposited in IEBR). *Holotype* of *Macroteleia dones*, ♀, **VIETNAM**: “Suoi Trai, Tra Mi, QN-DN [=Quang Nam-Da Nang], on grass in forest, 17.IV.1983, Lê Xuan Hue”, “Holotypus ♀ *Macroteleia dones* Kozlov et Lê 84” (deposited in IEBR). *Paratypes* of *Macroteleia dones*, 1 ♀, **VIETNAM**: “next to An Khe, on grass in forest, 4.XII.1978, Lê Xuan Hue” (IEBR); 1 ♂, “Son La Province, on grass, 2.V.1986, V. Triapitzin”, “Paratypus *Macroteleia dones* sp. n.” (IEBR).

Other material. **CHINA**: 3 ♀ + 2 ♂, Zhejiang, Hangzhou, West Lake, 30°15'N, 120°07'E, 25.VIII.2003, Qiong Wu, SCAU 000367–000371 (SCAU); 1 ♀, Fujian, Mt. Wuyi, 27°43'N, 117°42'E, 22–25.VIII.2007, Jie Zeng, SCAU 000372 (SCAU); 1 ♂, Taiwan, Kenting National Park, 21°57'N, 120°48'E, 31.V.2011, sweeping, Pu Tang, SCAU 000373 (SCAU); 1 ♀, Hunan, Changsha, 28°13'N, 112°55'E, 27.IX.1980, Xinwang Tong, SCAU 000374 (SCAU); 1 ♀, Guangdong, Chebaling National Nature Reserve, 24°43'N, 114°14'E, 25.V.2003, Jingxian Liu, SCAU 000375 (SCAU); 1 ♀ + 2 ♂, Guangdong, Chebaling National Nature Reserve, 24°43'N, 114°14'E, 21–23.VIII.2003, Jingxian Liu, SCAU 000376–000378 (SCAU); 1 ♂, Guangdong, Zijin County, Linjiang Town, 23°39'N, 114°41'E, 1.VIII.2003, Jingxian Liu, SCAU 000379 (SCAU); 1 ♀ + 1 ♂, Guangdong, Meizhou, Fenxi Forestry Farm, 24°38'N, 116°47'E, 28.VII.2003, Jianuan Zhou, SCAU 000380, 000381 (SCAU); 1 ♀, Guangdong, Qimuzhang Nature Reserve, 23°46'N, 115°18'E, 31.VII.2003, Jingxian Liu, SCAU 000382 (SCAU); 1 ♀ + 1 ♂, Guangdong, Xinfeng, Mt. Yunji, 24°04'N, 114°10'E, 19.VII.2003, Yanxia Song, SCAU 000383, 000384 (SCAU); 1 ♀, Guangdong, Mt. Nankun, 23°37.941'N, 113°50.182'E, 14.VII.2003, Jingxian Liu, SCAU 000385 (SCAU); 2 ♂, Guangdong, Mt. Nankun, 23°37.941'N, 113°50.182'E, 2.VII.2005, Zaifu Xu, SCAU 000386, 000387 (SCAU); 2 ♀, Guangdong, Mt. Nankun, 23°37.941'N, 113°50.182'E, 23.V.2010, Zaifu Xu, SCAU 000388, 000389 (SCAU); 1 ♀ + 1 ♂, Guangdong, Mt. Nankun, 23°37.941'N, 113°50.182'E, 25–30.VII.2010, yellow pan trap, Huayan Chen, SCAU 000390, 000391 (SCAU); 1 ♀, Guangdong, Mt. Nankun, 23°37.941'N, 113°50.182'E, 540 m, 4.VI.2011, yellow pan trap, Huayan Chen, SCAU 000392 (SCAU); 4 ♀, Guangdong, Zhaoqing, Xiwanggu, 23°13'N, 112°31'E, 2–6.VIII.2010, sweeping, Huayan Chen, SCAU 000393–000396 (SCAU); 1 ♀ + 1 ♂, Guangdong, Zhaoqing, Xiwanggu, 23°13'N, 112°31'E, 2–6.VIII.2010, yellow pan trap, Huayan Chen, SCAU 000397, 000398 (SCAU); 1 ♀, Guangdong, Guangzhou, Zengcheng, 23°14'N, 113°38'E, 15.XI.2010, Huiting Chen, SCAU 000399 (SCAU); 1 ♀ + 2 ♂, Guangdong, Guangzhou, Tianlu Lake, 23°13'N, 113°25'E, 6.X.2002, Zaifu Xu, SCAU 000400–000402 (SCAU); 1 ♀ + 1 ♂, Guangdong, Guangzhou, Tianlu Lake, 23°13'N, 113°25'E, 24.VI.2003, Jingxian Liu, SCAU 000403, 000404 (SCAU); 1 ♂, Guangdong, Guangzhou, Liuxihe, 23°44'N, 113°47'E, 1–4.VI.2003, Zaifu Xu, SCAU 000405 (SCAU); 1 ♂, Guangdong, Suixi County, Huanglue Town, 21°20.36'N, 110°18.61'E, 25.IX.2010, yellow pan trap, Huayan Chen, SCAU 000406 (SCAU); 2 ♀ + 1 ♂, Hainan, Mt. Yinggeling, 18°49´N, 109°11'E, 24–25.V.2007, Jingxian Liu, SCAU 000407–000409 (SCAU); 3 ♀, Hainan, Mt. Yinggeling, 18°49'N, 109°11'E, 22–25.V.2007, Jie Zeng, SCAU 000410–000412 (SCAU); 2 ♀, Hainan, Mt. Yinggeling, 18°49´N, 109°11'E, 23–25.V.2007, Bin Xiao, SCAU 000413, 000414 (SCAU); 1 ♀, Hainan, Mt. Yinggeling, 18°49´N, 109°11'E, 28.V.2007, Liqiong Weng, SCAU 000415 (SCAU); 1 ♀, Hainan, Mt. Yinggeling, 18°49'N, 109°11'E, 18.X.2007, Jingxian Liu, SCAU 000416 (SCAU); 3 ♀ + 1 ♂, Hainan, Mt. Yinggeling, 18°49'N, 109°11'E, 16–20.XI.2008, Huayan Chen, SCAU 000417–000420 (SCAU); 1 ♂, Hainan, Mt. Yinggeling, 18°49'N, 109°11'E, 18.XI.2008, Jiangli Tang, SCAU 000421 (SCAU); 5 ♀ + 7 ♂, Hainan, Mt. Yinggeling, 18°49'N, 109°11'E, 17–20.VII.2010, Huayan Chen, SCAU 000422–000433 (SCAU); 4 ♀ + 5 ♂, Hainan, Baisha, Mt. Jiujialing, 19°14'N, 109°24'E, 18.VII.2010, sweeping, Huayan Chen, SCAU 000434–000442 (SCAU); 2 ♀ + 1 ♂, Hainan, Baisha, Mt. Jiujialing, 19°14'N, 109°24'E, 17.VII.2010, yellow pan trap, Huayan Chen, SCAU 000443–000445 (SCAU); 8 ♀ + 4 ♂, Hainan, Wuzhishan National Nature Reserve, 18°51'N, 109°39'E, 16–18.V.2007, Jingxian Liu, SCAU 000446–000457 (SCAU); 1 ♀ + 2 ♂, Hainan, Wuzhishan National Nature Reserve, 18°51'N, 109°39'E, 16–18.V.2007, Jie Zeng, SCAU 000458–000460 (SCAU); 1 ♂, Hainan, Wuzhishan National Nature Reserve, 18°51'N, 109°39'E, 29.X.2007, Jingxian Liu, SCAU 000461 (SCAU); 2 ♀, Hainan, Wuzhishan National Nature Reserve, 18°51'N, 109°39'E, 28–30.X.2007, Jiemin Yao, SCAU 000462, 000463 (SCAU); 1 ♂, Hainan, Bawangling National Nature Reserve, 19°07'N, 109°03'E, 9.VI.2007, Jie Zeng, SCAU 000464 (SCAU); 1 ♂, Hainan, Bawangling National Nature Reserve, 19°07'N, 109°03'E, 1.V.2008, Chundan Hong, SCAU 000465 (SCAU); 2 ♀, Hainan, Jianfengling National Nature Reserve, 18°41'N, 108°49'E, 4.V.2008, Huayan Chen, SCAU 000466, 000467 (SCAU); 1 ♂, Hainan, Jianfengling National Nature Reserve, 18°41'N, 108°49'E, 12–14.VII.2008, Jingxian Liu, SCAU 000468 (SCAU); 1 ♀, Hainan, Mt. Diaoluo, 18°39'N, 109°53'E, 28.V–1.VI.2007, Jie Zeng, SCAU 000469 (SCAU); 5 ♀ + 5 ♂, Hainan, Mt. Diaoluo, 18°39'N, 109°53'E, 12–13.VII.2010, sweeping, Huayan Chen, SCAU 000470–000479 (SCAU); 1 ♀, Hainan, Sanya, Yacheng County, 18°22'N, 109°10'E, 21.XI.2008, Manman Wang, SCAU 000480 (SCAU); 1 ♂, Guangxi, Longshan Nature Reserve, 23°24.73'N, 108°31.92'E, 370m, 1.VII–2.VII.2011, yellow pan trap, Zaifu Xu, SCAU 000481 (SCAU); 2 ♂, Guangxi, Longshan Nature Reserve, 23°24.73'N, 108°31.92'E, 370m, 1–2.VII.2011, sweeping, Huiting Chen, SCAU 000482, 000483 (SCAU); 3 ♀ + 2 ♂, Yunnan, Gejiu, Manhao Town, 23°01'N, 103°20'E, 23.VII.2003, Jingxian Liu, SCAU 000484–000488 (SCAU); 1 ♀, Yunnan, Dehong County, Nabang Town, 24°43'N, 97°34'E, 15.V.2009, Manman Wang, SCAU 000489 (SCAU); 2 ♀, Yunnan, Xishuangbanna Primeval Forest Park, 22°01'N, 100°52'E, 30.VII.2003, Long Hu, SCAU 000490, 000491 (SCAU); 2 ♀ + 2 ♂, Yunnan, Hekou County, Nanxi Town, 22°37'N, 103°56'E, 20.VII.2003, Long Hu, SCAU 000492–000495 (SCAU).

### Macroteleia lamba

Saraswat & Sharma

http://species-id.net/wiki/Macroteleia_lamba

Plates 36 –41

Macroteleia lamba Saraswat and Sharma 1978: 13 (original description); Mani and Sharma 1982: 172 (description); Mukerjee 1994: 4 (variation); Lê 2000: 53, 61 (description, keyed, type information); Rajmohana 2006: 127 (description); Rajmohana 2007: 64 (description).
Macroteleia dores Lê 2000: 53, 58, 333 (original description, keyed), **syn. n.**

#### Description.

*Female*. Body length 3.33–5.26 mm (n=18).

*Color*. Body black; mandible dark brown; palpi yellow; legs pale brown throughout; A1 brown, A2–A5 dark brown, remainder of antenna black; fore wing hyaline.

*Head*. Transverse in dorsal view, 1.24–1.36× as wide as long, slightly wider than mesosoma; lateral ocellus contiguous with inner orbit of compound eye; POL 1.31–1.58× LOL; occipital carina continuous medially, irregularly crenulate throughout; central keel absent or weakly developed above interantennal process (Plates 37A, 38C, 40A); medial frons obliquely strigose ventrally, irregularly smooth dorsally; ventrolateral frons punctate rugose; frons below median ocellus densely punctate, interspaces coriaceous; ocellar triangle coriaceous, with scattered punctures; posterior vertex punctate reticulate; gena coarsely punctate rugose; length of A3 equal to length of A2.

*Mesosoma*. Cervical pronotal area densely punctate; dorsal pronotal area areolate; lateral pronotal area rugulose anteriorly, punctate rugulose posteriorly; netrion punctate rugulose; notaulus deep, distinctly foveolate; middle lobe of mesoscutum densely punctate, becoming denser anteriorly and at posterior end; lateral lobes of mesoscutum densely punctate, interspaces in part coriaceous; mesoscutellum densely punctate throughout; Metascutellum triangular (Plates 37B, 38D, 40B), strongly produced medially, extending into space between propodeal lobes; propodeum narrowly divided into two subtriangular lobes (Plates 37B, 38D, 40B), each side with several irregular longitudinal carinae medially, otherwise punctate rugulose; upper mesepisternum with a row of robust longitudinal carinae below subalar pit; lower mesepisternum punctate rugulose; mesopleural depression smooth (Plates 37C, 38E, 40C); metapleuron longitudinally striate throughout.

*Legs*. Slender; hind femur weakly swollen, 4.00–5.00× as long as its maximum width; hind tibia without spines over outer surface; hind basitarsus 11.00–11.80× as long as its maximum width.

*Wings*. Apex of fore wing extending from as far as anterior third to posterior margin of T4; R 1.62–2.44× as long as r-rs, R1 1.91–2.24× length of R.

*Metasoma*. Posterior margin of transverse sulcus on T2 straight or slightly convex (Plates 37D, 38F, 40D); sublateral tergal carinae developed on T1–T3; T1–T3 densely longitudinally striate medially, with scattered delicate punctures in interstices, punctate rugulose laterally; T4–T5 densely longitudinally striate throughout, with delicate punctures in interstices; T6 finely punctate dorsally, densely longitudinally striate laterally, with scattered small punctures in interstices; length of T3 0.91–1.15× length of T6; T5 distinctly longer than wide; S2–S6 densely longitudinally striate, with delicate punctures in interstices; prominent longitudinal median carina present on S2–S5.

*Male*. Differing from female as follows: body length 3.57–5.26 mm (n=6); A1 brown, remainder of antenna dark brown to black; hind coxa blackish; metascutellum distinctly transverse (Plate 41A), posterior margin slightly pointed medially, longitudinally carinate; propodeum continuous medially, not divided into two separated lobes, posterior margin narrowly notched medially, each side with several irregular longitudinal carinae medially, otherwise punctate rugulose, covered by dense, recumbent, white setae; T1 sparsely longitudinally striate medially, with rugulose sculpture in interstices anteriorly, punctate rugulose laterally; T2–T3 densely longitudinally striate medially, with numerous delicate punctures in interstices, punctate rugulose laterally; T4–T5 densely longitudinally striate throughout, with numerous delicate punctures in interstices; T6–T7 longitudinally punctate rugulose; T6 distinctly longer than wide; length of T6 1.07–1.18× length of T7; T7 subtriangular, apex pointed (Plate 41B); length of T7 2.15–2.53× length of S7; S7 longitudinally punctate rugulose; prominent longitudinal median carina present on S2–S6.

**Plate 36.**
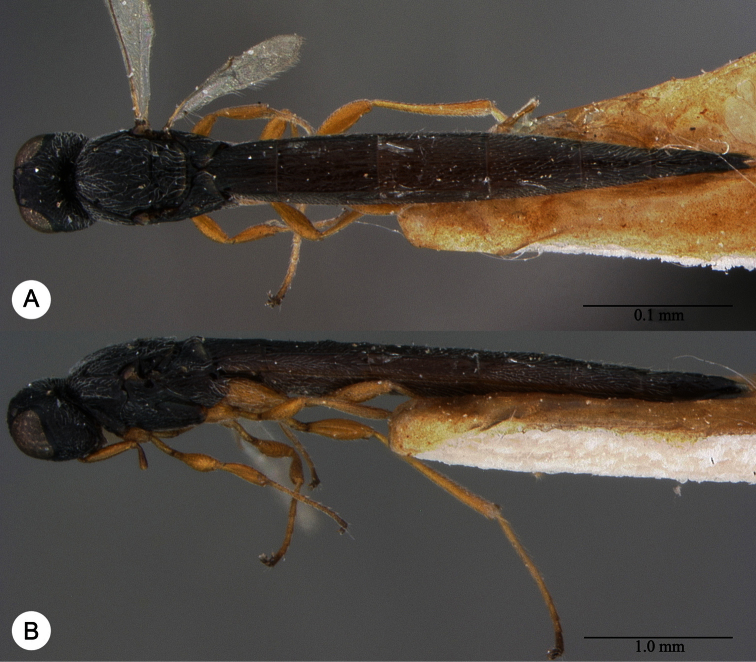
*Macroteleia lamba* Saraswat & Sharma, holotype, female. **A** Dorsal habitus **B** Lateral habitus.

**Plate 37.**
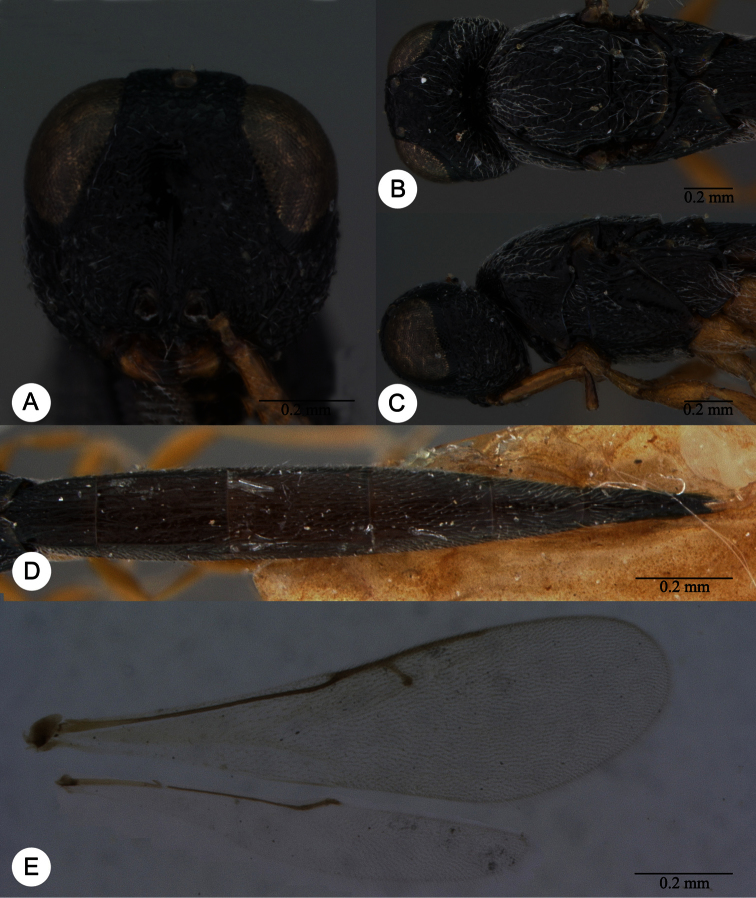
*Macroteleia lamba* Saraswat & Sharma, holotype, female. **A** Head, anterior view **B** Head and mesosoma, dorsal view **C** Head and mesosoma, lateral view **D** Metasoma, dorsal view **E** Fore and hind wing.

**Plate 38.**
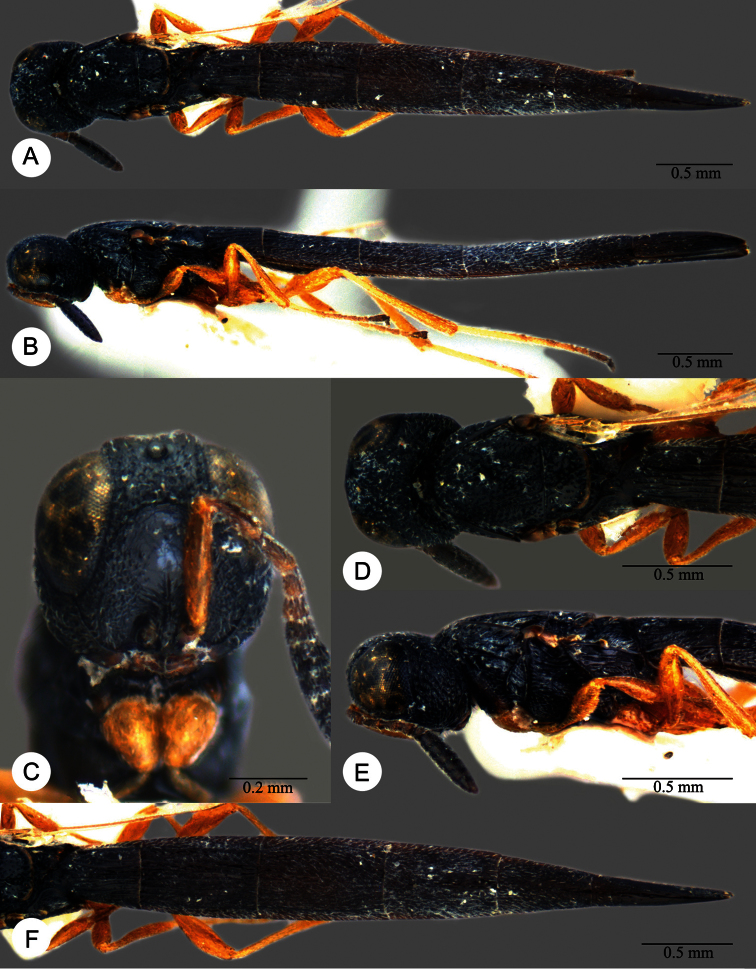
*Macroteleia dores* Kozlov & Lê, holotype, female. **A** Dorsal habitus **B** Lateral habitus **C** Head, anterior view **D** Head and mesosoma, dorsal view **E** Head and mesosoma, lateral view **F** Metasoma, dorsal view.

**Plate 39.**
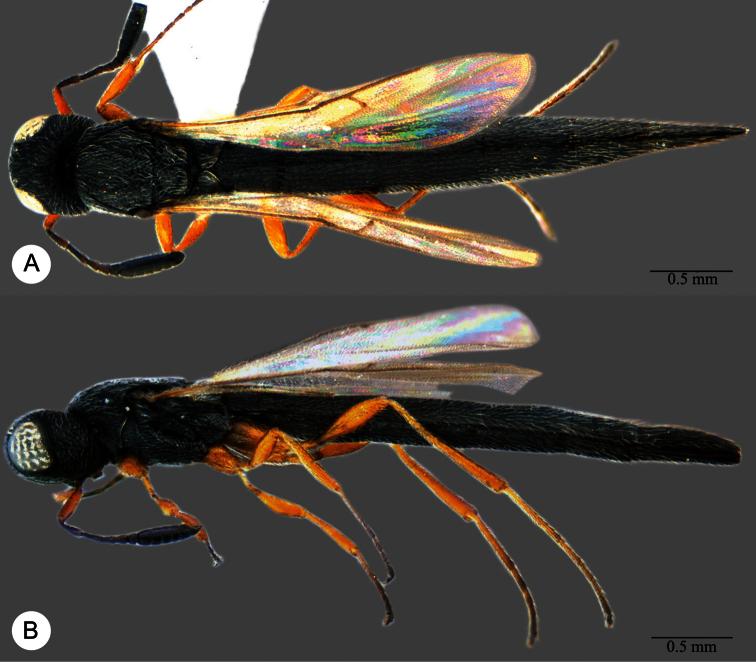
*Macroteleia lamba* Saraswat & Sharma, female from Yunnan, Jinggu County, Weiyuan Town. **A** Dorsal habitus **B** Lateral habitus.

**Plate 40.**
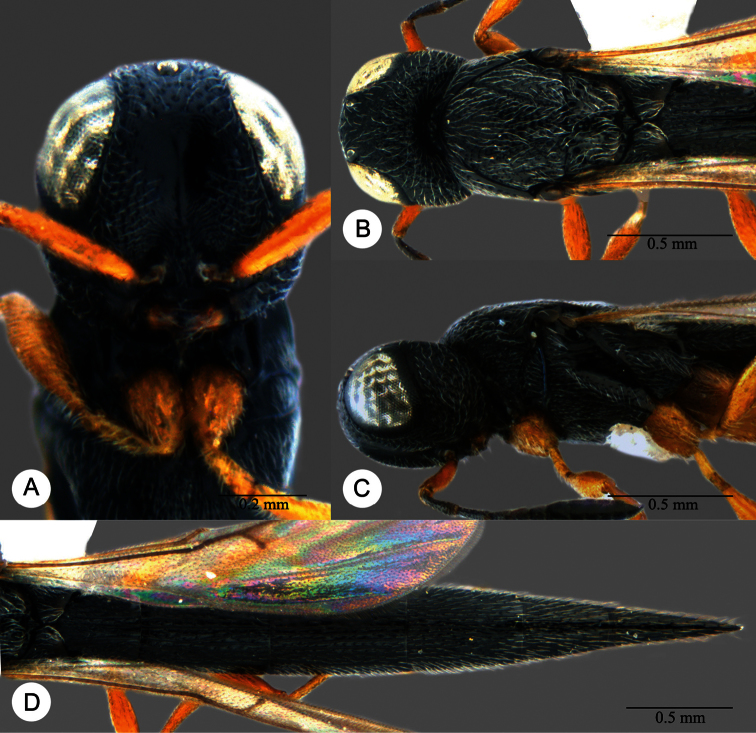
*Macroteleia lamba* Saraswat & Sharma, female from Yunnan, Jinggu County, Weiyuan Town. **A** Head, anterior view **B** Head and mesosoma, dorsal view **C** Head and mesosoma, lateral view **D** Metasoma, dorsal view.

**Plate 41.**
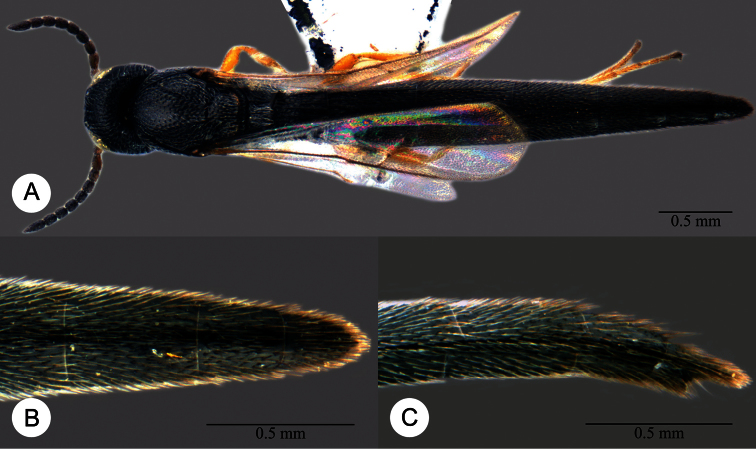
*Macroteleia lamba* Saraswat & Sharma, male from Guangdong, Zijin County, Linjiang Town. **A** Dorsal habitus **B** Apex of metasoma, dorsal view **C** Apex of metasoma, lateral view.

#### Distribution.

China (Guangdong, Hainan, Yunnan); India; Vietnam; Thailand. Link to distribution map [http://hol.osu.edu/map-large.html?id=4825].

#### Material examined.

*Holotype* of *Macroteleia lamba*, ♀: **INDIA**: “Coorg Survey, School of Entomology, St. John’s College, Agra-282002, India, 18.1. Kasaragod, M.S. Mani & Party, 31.III–1–3.IV.1977”, “Holotype”, “*Macroteleia lamba* Saraswat, $” (deposited in USNM). *Holotype* of *Macroteleia dores*, $: **VIETNAM**: “Buon Luoi, grass, 16.VI.1982”, “Holotypus ♀ *Macroteleia dores* Kozlov et Lê 84” (deposited in IEBR). *Paratype* of *Macroteleia dores*, 1 ♂, **VIETNAM**: “Buon Luoi, GL-KT [=Gia Lai-Kon Tum], grass, 14.VII.1981”, “Paratypus ♂ *Macroteleia dores* sp. n. Kozlov et Lê” (IEBR).

#### Other material.

**CHINA**: 1 ♂, Guangdong, Chebaling National Nature Reserve, 24°43'N, 114°14'E, 10.VII.2003, Zaifu Xu, SCAU 000118 (SCAU); 4 ♀, Guangdong, Chebaling National Nature Reserve, 24°43'N, 114°14'E, 21–23.VIII.2003, Jingxian Liu, SCAU 000119–000122 (SCAU); 1 ♀, Guangdong, Nanling National Nature Reserve, 24°54'N, 113°00'E, 11.IX.2004, Yanjing Wen, SCAU 000123 (SCAU); 1 ♀, Guangdong, Qimuzhang Nature Reserve, 23°46'N, 115°18'E, 31.VII.2003, Jingxian Liu, SCAU 000124 (SCAU); 1 ♂, Guangdong, Zijin County, Linjiang Town, 23°39'N, 114°41'E, 28.VII.2003, Jingxian Liu, SCAU 000125 (SCAU); 3 ♀ + 3 ♂, Guangdong, Zijin County, Linjiang Town, 23°39'N, 114°41'E, 1.VIII.2003, Jingxian Liu, SCAU 000126–000131 (SCAU); 1 ♀, Guangdong, paddy field, Zhuhai, Doumen, 22°33.366'N, 113°13.297'E, 11.IX.2010, Xin Yuan, SCAU 000132 (SCAU); 1 ♀, Hainan, Mt. Yinggeling, 18°49'N, 109°11'E, 28.V.2007, Liqiong Weng, SCAU 000133 (SCAU); 1 ♀, Hainan, Jianfengling National Nature Reserve, 18°41'N, 108°49'E,22–23.X.2007, Jingxian Liu, SCAU 000134 (SCAU); 1 ♀, Yunnan, Jinggu County, Weiyuan Town, 23°39'N, 100°42'E, 4.X.2004, Jingxian Liu, SCAU 000135 (SCAU); 3 ♀, Yunnan, Jinghong, on the bank of Lancang River, 21°59'N, 100°49'E, 2.X.2004, Jingxian Liu, SCAU 000136–000138 (SCAU). **THAILAND**: 1 ♀, Chiang Mai: Maerim, 21–22.XII.2000, MT, R. A. Beaver, No. 24062 (RABC); 1 ♀, Chiang Mai: Maerim, 20.XII.2002, FIT, R. A. Beaver, No. 25927 (RABC).

#### Comments.

*Macroteleia superans* Kieffer was described from a male collected at Mt. Makiling in the Philippine Islands by C.F. Baker. Later, in Kieffer’s Das Tierreich (Kieffer 1926) treatment of the species he added the locality of Mt. Banahao for the species. We searched for the type material at the U.S. National Museum of Natural History (Washington, DC) where much of Baker’s material is now held. Only a single specimen of *Macroteleia superans* was found, the specimen from Mt. Banahao. The location of the holotype is unknown. This non-type specimen is conspecific with *Macroteleia lamba*. Without the holotype, though, we prefer not to propose that the name is synonymous.

### Macroteleia livingstoni

Saraswat

http://species-id.net/wiki/Macroteleia_livingstoni

Plates 42 46

Macroteleia livingstoni Saraswat 1982: 348 (original description).

#### Description.

*Female*. Body length 3.28–4.08 mm (n=20).

*Color*. Body black; mandible dark brown; palpi light brown; legs pale brown throughout; A1 pale brown, A2–A6 brown, remainder of antenna black; fore wing hyaline.

*Head*. Transverse in dorsal view, 1.20–1.34× as wide as long, slightly wider than mesosoma; lateral ocellus contiguous with inner orbit of compound eye; POL 1.31–1.45× LOL; occipital carina continuous medially; central keel weakly developed above interantennal process (Plates 43A, 45A); medial frons obliquely strigose ventrally, irregularly smooth dorsally; ventrolateral frons punctate rugose; frons below median ocellus sparsely punctate; ocellar triangle smooth, with scattered punctures; posterior vertex densely punctate; gena punctate reticulate; length of A3 0.83–1.00× length of A2.

*Mesosoma*. Cervical pronotal area densely punctate; dorsal pronotal area densely coarsely punctate; lateral pronotal area smooth in upper anterior angle, punctate rugose posteriorly; netrion punctate rugulose; notaulus narrow, but distinctly foveolate; middle lobe of mesoscutum densely punctate anteriorly and at posterior end, usually sparsely punctate on a small area across middle; lateral lobe of mesoscutum densely punctate throughout; mesoscutellum densely punctate throughout; metascutellum transverse (Plates 43B, 45B), posterior margin slightly pointed medially, longitudinally carinate; propodeum continuous medially (Plates 43B, 45B), not divided into two separated lobes, posterior margin narrowly notched medially, each side with several irregular longitudinal carinae medially, otherwise punctate rugulose, covered by dense, recumbent, white setae; upper mesepisternum with a row of weak longitudinal carinae or a ledge below subalar pit; lower mesepisternum variably smooth to punctate rugulose; mesopleural depression smooth (Plates 43C, 45C); metapleuron longitudinally striate dorsally, punctate rugulose ventrally.

*Legs*. Slender; hind femur weakly swollen, 3.86–4.69× as long as its maximum width; hind tibia without spines over outer surface; hind basitarsus 10.50–13.00× as long as its maximum width.

*Wings*. Apex of fore wing extending from as far as posterior fifth to posterior margin of T4; R 1.82–2.18× as long as r-rs, R1 1.65–2.18× length of R.

*Metasoma*. Posterior margin of transverse sulcus on T2 slightly convex (Plates 43D, 45D); sublateral tergal carinae developed on T1–T3; T1–T3 densely longitudinally striate medially, with scattered delicate punctures in interstices, punctate rugulose laterally; T4–T5 densely longitudinally striate throughout, with delicate punctures in interstices; T6 finely punctate dorsally, densely longitudinally striate laterally, with scattered small punctures in interstices; length of T3 1.00–1.13× length of T6; T5 slightly longer than wide; S2–S6 densely longitudinally striate, with delicate punctures in interstices; prominent longitudinal median carina present on S2–S5.

*Male*. Differing from female as follows: body length 3.29–3.92 mm (n=20); A1 pale brown, remainder of antenna dark brown, becoming darker apically; T5–T6 moderately longitudinally striate, with delicate punctures in interstices; T7 punctate rugulose throughout; T6 distinctly longer than wide; length of T6 1.00–1.19× length of T7; T7 subtriangular, apex pointed (Plate 46B); length of T7 1.56–2.40× length of S7; S6–S7 longitudinally punctate rugulose; prominent longitudinal median carina present on S2–S6.

**Plate 42.**
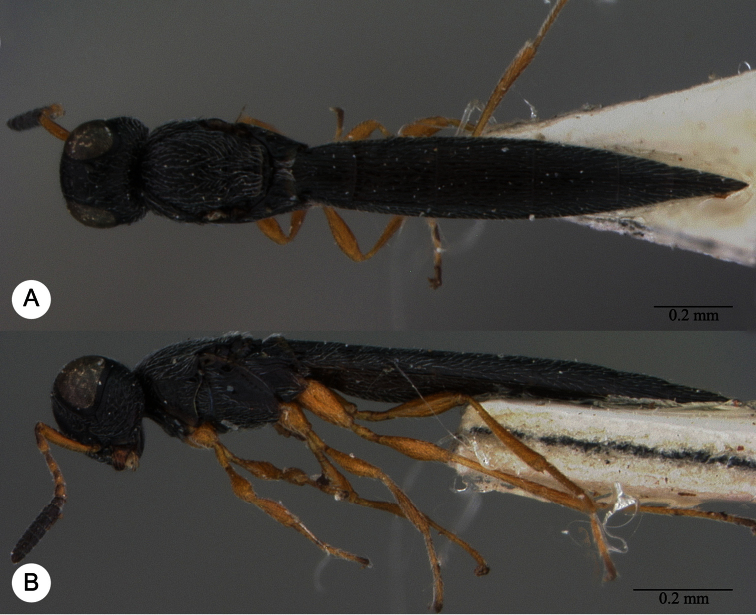
*Macroteleia livingstoni* Saraswat, holotype, female. **A** Dorsal habitus **B** Lateral habitus.

**Plate 43.**
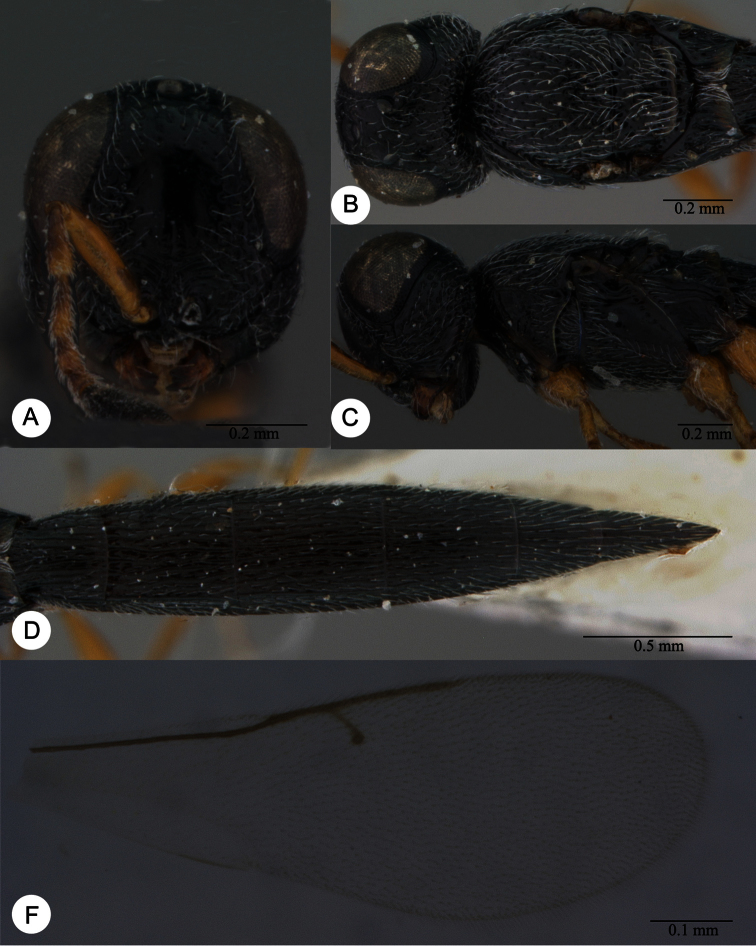
*Macroteleia livingstoni* Saraswat, holotype, female. **A** Head, anterior view **B** Head and mesosoma, dorsal view **C** Head and mesosoma, lateral view **D** Metasoma, dorsal view **E** Fore wing.

**Plate 44.**
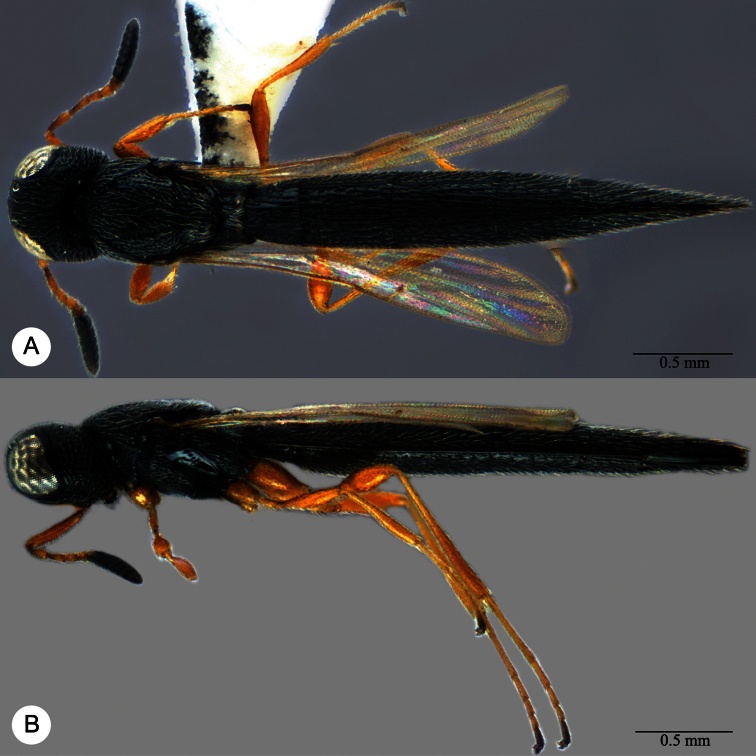
*Macroteleia livingstoni* Saraswat, female from Guangdong, Zhaoqing, Xiwanggu. **A** Dorsal habitus **B** Lateral habitus.

**Plate 45.**
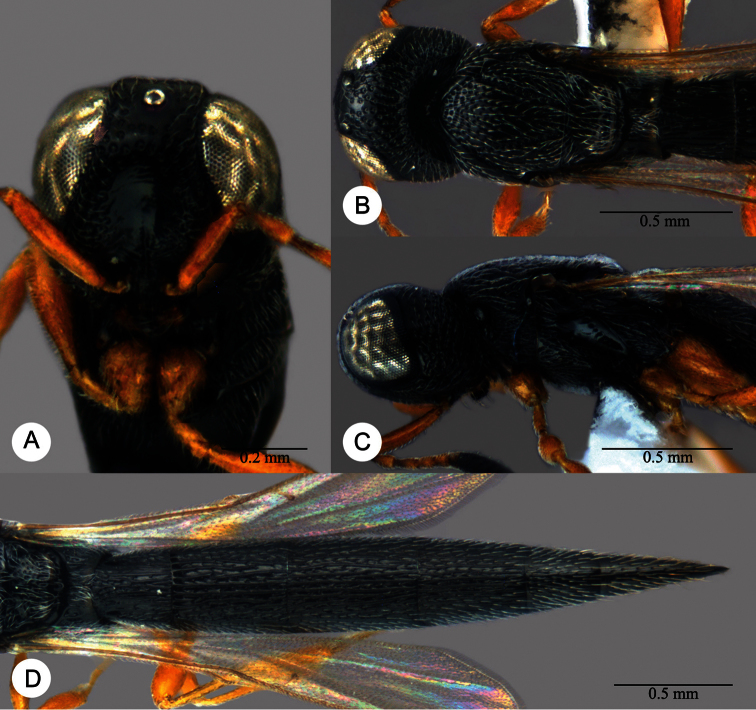
*Macroteleia livingstoni* Saraswat, female from Guangdong, Zhaoqing, Xiwanggu. **A** Head, anterior view **B** Head and mesosoma, dorsal view **C** Head and mesosoma, lateral view **D** Metasoma, dorsal view.

**Plate 46.**
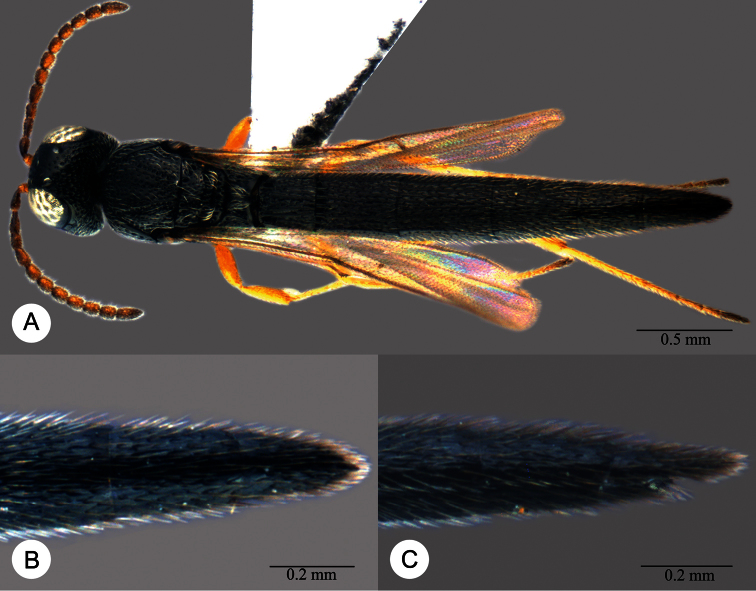
*Macroteleia livingstoni* Saraswat, male from Hainan, Baisha, Mt. Jiujialing. **A** Dorsal habitus **B** Apex of metasoma, dorsal view **C** Apex of metasoma, lateral view.

#### Diagnosis.

*Macroteleia livingstoni* is similar to *Macroteleia emarginata* in body shape and color, but can be distinguished by its smller size and reticulate gena. The male of *Macroteleia livingstoni* is also similar to that of *Macroteleia lamba*, but can be distinguished by the fact that the frons below the median ocellus is sparsely punctate with the interspaces smooth (densely punctate, interspaces coriaceous in *Macroteleia lamba*); ocellar triangle smooth, with scattered punctures (coriaceous, with scattered punctures in *Macroteleia lamba*).

#### Distribution.

China (Hubei, Guangdong, Hainan, Guizhou, Guangxi, Yunnan); India. Link to distribution map [http://hol.osu.edu/map-large.html?id=4831].

#### Material examined.

*Holotype*, ♀: **INDIA**: “Karnataka Survey, School of Entomology, St. John’s College, Agra-282002, India, 20.6.Walayar, M.S. Mani & Party, 9–11.V.1978”, “Holotype”, “*Macroteleia livingstoni* Saraswat, $” (deposited in USNM).

#### Other material.

**CHINA**: 1 ♀ Guangdong, Nanling National Nature Reserve, 24°54'N, 113°00'E, 8–17.VIII.2010, Huayan Chen, SCAU 000189; 3 ♀, Hubei, Huanggang, Mt. Dahu, 31°27'N, 114°32'E, VIII.2009, Chunhong Zheng, SCAU 000497–000499 (SCAU); 2 ♀ + 4 ♂, Guangdong, Chebaling National Nature Reserve, 24°43'N, 114°14'E, 21–23.VIII.2003, Jingxian Liu, SCAU 000190–000195 (SCAU); 3 ♀ + 1 ♂, Guangdong, Zijin County, Linjiang Town, 23°39'N, 114°41'E, 1.VIII.2003, Jingxian Liu, SCAU 000196–000199 (SCAU); 7 ♀ + 2 ♂, Guangdong, Meizhou, Fenxi Forestry Farm, 24°38'N, 116°47'E, 28.VII.2003, Jianuan Zhou, SCAU 000200–000208 (SCAU); 1 ♀, Guangdong, Qimuzhang Nature Reserve, 23°46'N, 115°18'E, 31.VII.2003, Jingxian Liu, SCAU 000209 (SCAU); 1 ♀, Guangdong, Boluo County, Yangcun Town, 23°25'N, 114°29'E, VIII. 2007, SCAU 000210 (SCAU); 48 ♀ + 1 ♂, Guangdong, Zhaoqing, Xiwanggu, 23°13'N, 112°31'E, 2–6.VIII.2010, sweeping, Huayan Chen, SCAU 000211–000259 (SCAU); 1 ♀ + 4 ♂, Guangdong, Guangzhou, Tianlu Lake, 23°13'N, 113°25'E, 24.VI.2003, Jingxian Liu, SCAU 000260–000264 (SCAU); 6 ♀ + 2 ♂, Hainan, Mt. Yinggeling, 18°49'N, 109°11'E, 18.X.2007, Jingxian Liu, SCAU 000265–000272 (SCAU); 2 ♀, Hainan, Mt. Yinggeling, 18°49'N, 109°11'E, 16–20.XI.2008, Huayan Chen, SCAU 000273, 000274 (SCAU); 7 ♀ + 2 ♂, Hainan, Mt. Yinggeling, 18°49'N, 109°11'E, 17.XII–20.XII.2010, Huayan Chen, SCAU 000275–000283 (SCAU); 10 ♀ + 11 ♂, Hainan, Baisha, Mt. Jiujialing, 19°14'N, 109°24'E, 18.VII.2010, sweeping, Huayan Chen, SCAU 000284–000304 (SCAU); 2 ♀, Hainan, Mt. Diaoluo, 18°39'N, 109°53'E, 29.V.2007, Bin Xiao, SCAU 000305, 000306 (SCAU); 1 ♀, Hainan, Mt. Diaoluo, 12–13.VII.2010, 18°39'N, 109°53'E, Huayan Chen, SCAU 000307 (SCAU); 1 ♀ + 1 ♂, Guizhou, Guiyang Forest Park, 26°33'N, 106°44'E, 23.IX.2007, Cuihong Xie, SCAU 000308, 000309 (SCAU); 1 ♀ + 1 ♂, Guangxi, Longshan Nature Reserve, 23°24.73'N, 108°31.92'E, 370 m, 1–2.VII.2011, yellow pan trap, Zaifu Xu, SCAU 000310, 000311 (SCAU); 4 ♀ + 6 ♂, Guangxi, Longshan Nature Reserve, 23°24.73'N, 108°31.92'E, 1–2.VII.2011, sweeping, Huayan Chen, SCAU 000312–000321 (SCAU); 1 ♂, Guangxi, Shanlin, Longshan Nature Reserve, 23°24.73'N, 108°31.92'E, 1–2.VII.2011, sweeping, Huiting Chen, SCAU 000322 (SCAU); 1 ♂, Yunnan, Yingjiang, Tongbiguan, 24°15'N, 97°49'E, 16.V.2009, Manman Wang, SCAU 000323 (SCAU).

### Macroteleia peliades

Kozlov & Lê

http://species-id.net/wiki/Macroteleia_peliades

Plates 47 50

Macroteleia peliades Lê 2000: 54, 62, 335 (original description, keyed).

#### Description.

*Female*. Body length 7.28–8.50 mm (n=5).

*Color*. Body black; mandible reddish brown; palpi yellow; legs yellow throughout; A1–A5 yellow, A6 dark brown, remainder of antenna black; fore wing hyaline.

*Head*. Transverse in dorsal view, 1.44–1.52× as wide as long, slightly wider than mesosoma; lateral ocellus contiguous with inner orbit of compound eye; POL 1.53–1.58× LOL; occipital carina continuous medially, irregularly crenulate; central keel absent (Plate 49A); medial frons smooth dorsally, punctate rugulose ventrally; ventrolateral frons rugose punctate; frons below median ocellus densely punctate; vertex densely punctate, becoming punctate reticulate posteriorly; gena coarsely punctate reticulate; length of A3 1.28–1.48× length of A2.

*Mesosoma*. Cervical pronotal area finely punctate; dorsal pronotal area densely coarsely punctate; lateral pronotal area smooth anteriorly, irregularly punctate posteriorly; netrion rugulose; notaulus narrow, foveolate; middle lobe of mesoscutum densely punctate, becoming punctate reticulate anteriorly, interspaces along notaulus in part with microsculpture; lateral lobes of mesoscutum irregularly punctate; mesoscutellum densely punctate throughout; metascutellum transverse (Plate 49B), posterior margin straight or slightly convex medially, longitudinally carinate; propodeum continuous medially (Plate 49B), not divided into two separated lobes, posterior margin narrowly notched medially, each side with several irregular longitudinal carinae medially, otherwise with rugose sculpture covered by dense, recumbent, white setae; upper mesepisternum with a row of robust longitudinal carinae below subalar pit; lower mesepisternum densely punctate to punctate rugulose; mesopleural depression smooth (Plate 49C); metapleuron longitudinally punctate rugulose.

*Legs*. Slender; hind femur somewhat swollen, 3.57–3.85× as long as its maximum width; hind tibia without spines over outer surface; hind basitarsus 8.18–9.09× as long as its maximum width.

*Wings*. Apex of fore wing extending as far as mid-length to anterior 2/3 of T4; R 1.68–1.96 × as long as r-rs, R1 1.64–1.94× length of R.

*Metasoma*. Posterior margin of transverse sulcus on T2 straight (Plate 49D); sublateral tergal carinae well developed on T1–T3; T1 longitudinally striate medially, with scattered punctures in interstices anteriorly, punctate rugulose laterally; T2, T3 longitudinally striate medially, with delicate punctures in interstices, punctate rugulose laterally; T4, T5 longitudinally striate, with numerous delicate punctures in interstices throughout; T6 punctate rugulose dorsally, densely longitudinally striate laterally, with scattered small punctures in interstices; length of T3 1.03–1.23× length of T6; T5 much longer than wide; S2–S6 sparsely longitudinally striate, with numerous punctures in interstices; prominent longitudinal median carina strongly developed on S2–S5.

**Plate 47.**
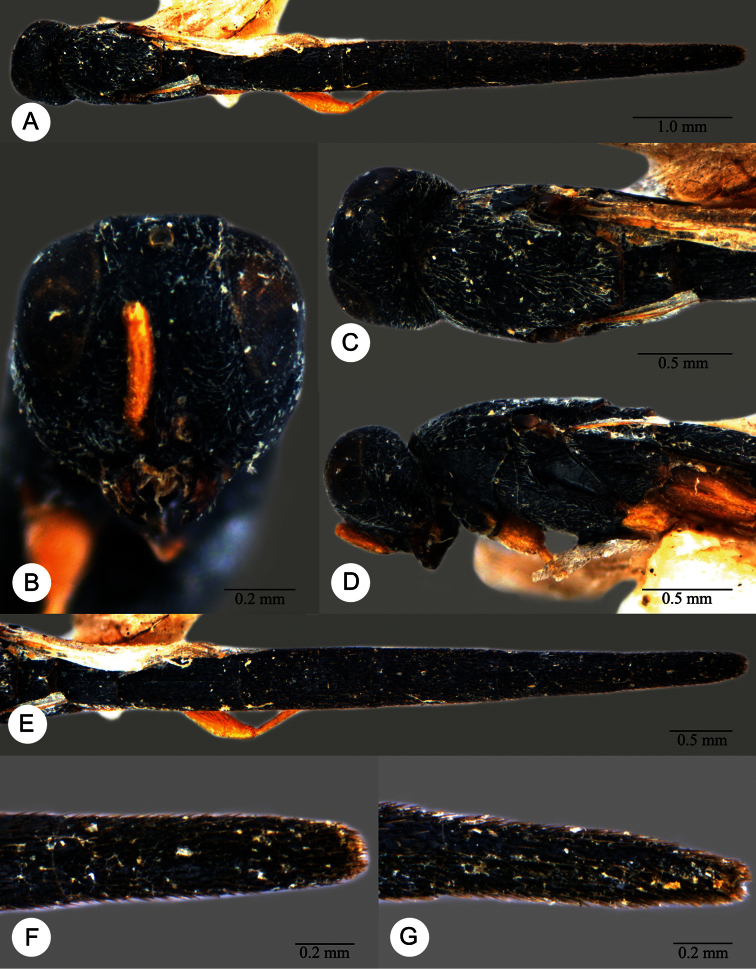
*Macroteleia peliades* Kozlov & Lê, holotype, male. **A** Dorsal habitus **B** Head, anterior view **C** Head and mesosoma, dorsal view **D** Head and mesosoma, lateral view **E** Metasoma, dorsal view **F** Apex of metasoma, dorsal view **G** Apex of metasoma, lateral view.

*Male*. Differing from female as follows: body length 7.00–8.00 mm (n=4); antenna yellow to brown, becoming darker apically; hind coxa yellow or dark brown to black; T1 sparsely longitudinally striate, with scattered punctures in interstices; T2–T7 densely longitudinally striate, with numerous punctures in interstices; T6 distinctely longer than wide; length of T6 1.30–1.96× length of T7; T7 transverse, apex truncate (Plates 47F, 50B); length of T7 equal to length of S7; S2–S5 sparsely longitudinally striate, with numerous punctures in interstices; S6, S7 longitudinally punctate rugulose.

**Plate 48.**
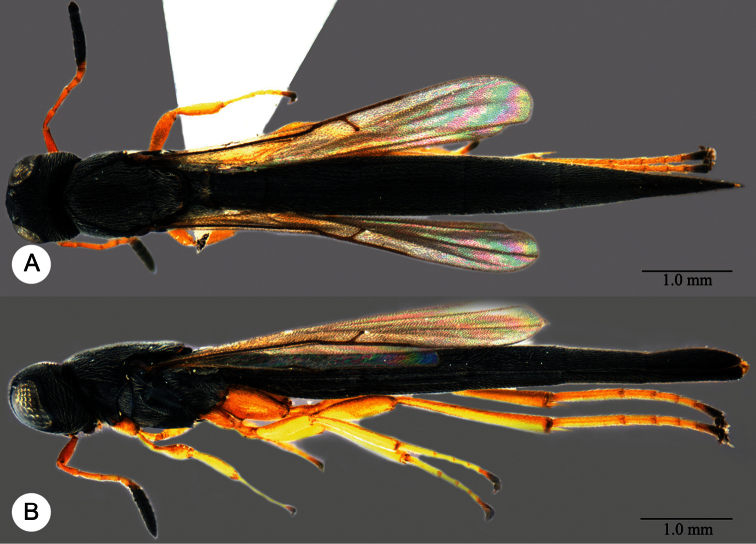
*Macroteleia peliades* Kozlov & Lê, female from Guangxi, Longshan Nature Reserve. **A** Dorsal habitus **B** Lateral habitus.

**Plate 49.**
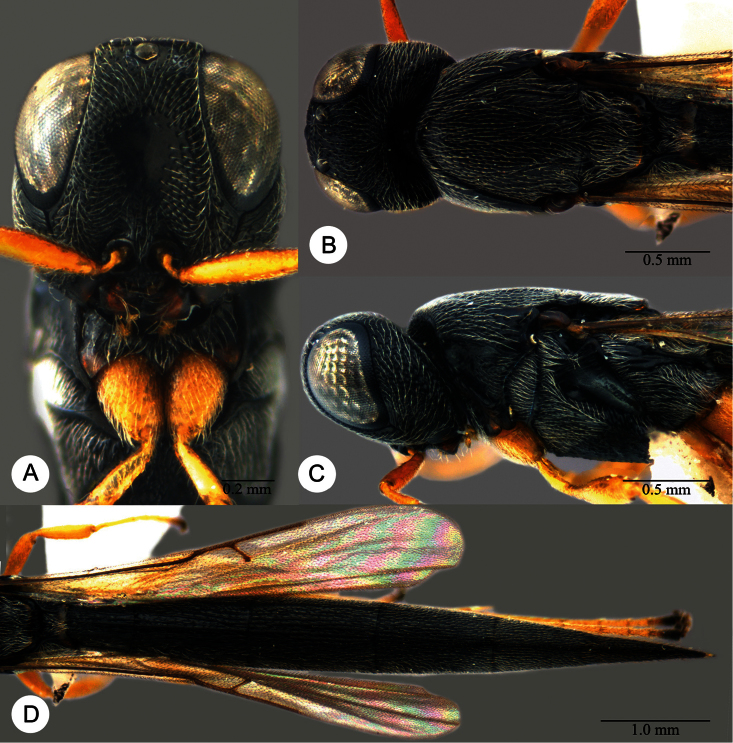
*Macroteleia peliades* Kozlov & Lê, female from Guangxi, Longshan Nature Reserve. **A** Head, anterior view **B** Head and mesosoma, dorsal view **C** Head and mesosoma, lateral view **D** Metasoma, dorsal view.

**Plate 50.**
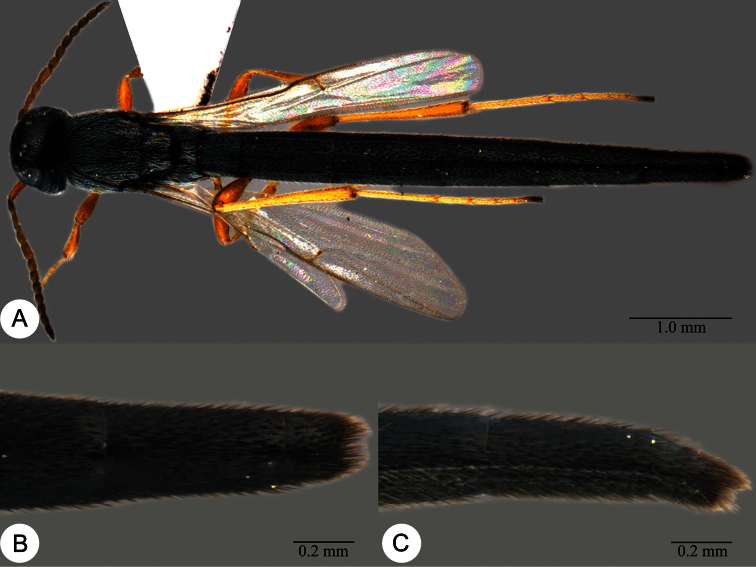
*Macroteleia peliades* Kozlov & Lê, male from Guangxi, Longshan Nature Reserve. **A** Dorsal habitus **B** Apex of metasoma, dorsal view **C** Apex of metasoma, lateral view.

#### Diagnosis.

This species can be easily separated from the other Chinese *Macroteleia* by its large size.

#### Distribution.

China (Zhejiang, Guangdong, Guangxi); Vietnam. Link to distribution map [http://hol.osu.edu/map-large.html?id=179764].

#### Material examined.

*Holotype*, ♂, **VIETNAM**: “Van Mai, HSB [=Hoa Binh], 30.V.1982”, “Holotypus ♂, *Macroteleia peliades* Kozlov et Lê, 84” (deposited in IEBR).

#### Other material.

**CHINA**: 1 ♀, Zhejiang, Mt. Qingliangfeng, 30°04'N, 118°52'E, 9.VIII.2005, Min Shi, SCAU 000024 (SCAU); 1 ♀, Guangdong, Chebaling National Nature Reserve, 24°43'N, 114°14'E, 25.V.2002, Jingxian Liu, SCAU 000025 (SCAU); 1 ♀ + 1 ♂, Guangdong, Mt. Nankun, 23°37.941'N, 113°50.182'E, 12.V.2004, Zaifu Xu, SCAU 000026, 000027 (SCAU); 1 ♀ + 1 ♂, Guangdong, Nanling National Nature Reserve, 24°54'N, 113°00'E, 16.VII–21.VII.2008, Zaifu Xu, SCAU 000028, 000029 (SCAU); 1 ♀ + 1 ♂, Guangxi, Longshan Nature Reserve, 23°24.727'N, 108°31.918'E, 1.VII–2.VII.2011, yellow pan trap, Zaifu Xu et al., SCAU 000030, 000031 (SCAU).

**Note.** Female of this species is described for the first time.

### Macroteleia rufa

Szelényi

http://species-id.net/wiki/Macroteleia_rufa

Plates 51 52

Macroteleia rufa Szelényi 1938: 91, 92 (original description); Kozlov and Kononova 1987: 94, 95 (keyed); Kozlov and Kononova 1990: 189, 190, 197 (description, keyed); Petrov 1994: 96 (comparison with *Macroteleia angelovi* Petrov); Kononova 1995: 70, 72 (keyed); Kononova and Petrov 2003: 605 (keyed); Kononova and Kozlov 2008: 231, 235 (description, keyed, synonym).
Macroteleia eremicola Priesner 1951: 137 (original description); Masner and Muesebeck 1968: 39 (type information); Kononova and Kozlov 2008: 235 (junior synonym of *Macroteleia rufa* Szelényi).

#### Description.

*Female*. Body length 4.55–6.62 mm (n=10).

*Color*. Head and mesosoma yellowish brown; metasoma with T1 and T6 variably dark brown to black, otherwise yellow; mandible dark brown; palpi yellow; legs yellow throughout; A1 yellow, A2–A5 dark brown, remainder of antenna black; fore wing hyaline.

*Head*. Transverse in dorsal view, 1.30–1.44× as wide as long, slightly wider than mesosoma; lateral ocellus contiguous with inner orbit of compound eye; POL 1.36–1.46× LOL; occipital carina continuous medially, irregularly punctate; central keel weakly developed above interantennal process (Plate 52A); medial frons punctate rugulose ventrally, irregularly smooth dorsally; ventrolateral frons punctate rugose; frons below median ocellus densely punctate; vertex densely punctate, interspaces in part with microsculpture; gena punctate rugose; length of A3 1.09–1.19× length of A2.

*Mesosoma*. Cervical pronotal area densely punctate; dorsal pronotal area areolate rugose; lateral pronotal area longitudinally striate dorsally, punctate rugulose ventrally; netrion punctate rugulose; notaulus narrow, irregularly foveolate; middle lobe of mesoscutum densely punctate, becoming denser anteriorly and at posterior end; lateral lobe of mesoscutum densely punctate, interspaces in part with microsculpture; mesoscutellum densely punctate throughout; metascutellum triangular (Plate 52B), posterior margin strongly produced medially, extending into space between propodeal lobes; propodeum narrowly divided into two subtriangular lobes (Plate 52B), each side with several irregular longitudinal carinae medially, otherwise punctate rugulose, covered by dense, recumbent, white setae; upper mesepisternum with a row of strong longitudinal carinae below subalar pit; lower mesepisternum longitudinally punctate rugulose; mesopleural depression smooth (Plate 52C); metapleuron longitudinally striate dorsoventrally, punctate rugulose medially.

*Legs*. Slender; hind femur weakly swollen, 4.10–4.37× as long as its maximum width; hind tibia without spines over outer surface; hind basitarsus 10.14–11.33× as long as its maximum width.

*Wings*. Apex of fore wing extending from as far as anterior third to mid-length of T4; R 1.38–2.08× as long as r-rs, R1 1.67–2.08× length of R.

*Metasoma*. Posterior margin of transverse sulcus on T2 strongly convex (Plate 52D); sublateral tergal carinae developed on T1–T3; T1 densely longitudinally striate medially, with scattered punctures in interstices anteriorly, rugulose laterally; T2–T3 densely longitudinally striate medially, with delicate punctures in interstices, punctate rugulose laterally; T4–T5 densely finely longitudinally striate throughout, with delicate punctures in interstices; T6 finely punctate dorsally, densely longitudinally striate laterally, with scattered small punctures in interstices; length of T3 0.82–0.94× length of T6; T5 distinctly longer than wide; S2–S4 longitudinally striate, with finely punctate rugulose interstices; S5–S6 longitudinally striate, with finely punctate interstices; prominent longitudinal median carinae present on S2–S5.

*Male*. No specimens were available for this study.

**Plate 51.**
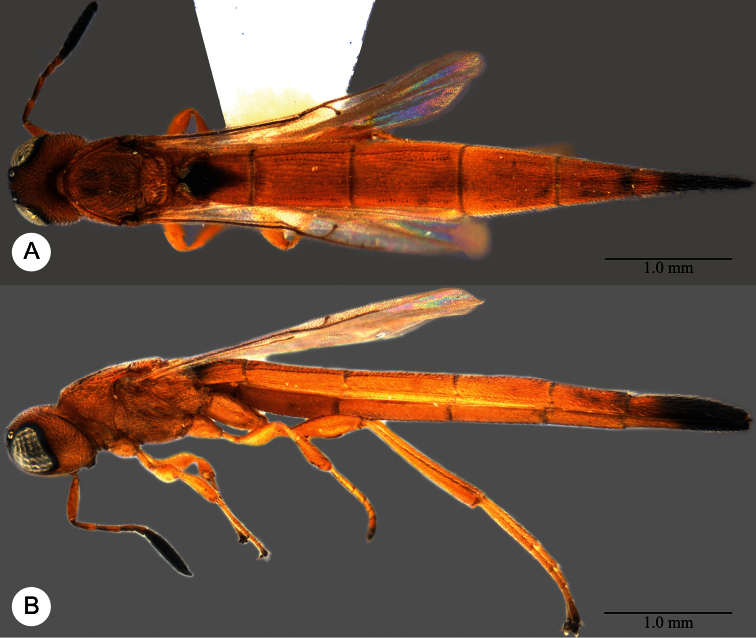
*Macroteleia rufa* Szelényi, female from Guangdong, Guangzhou, Tianlu Lake. **A** Dorsal habitus **B** Lateral habitus.

**Plate 52.**
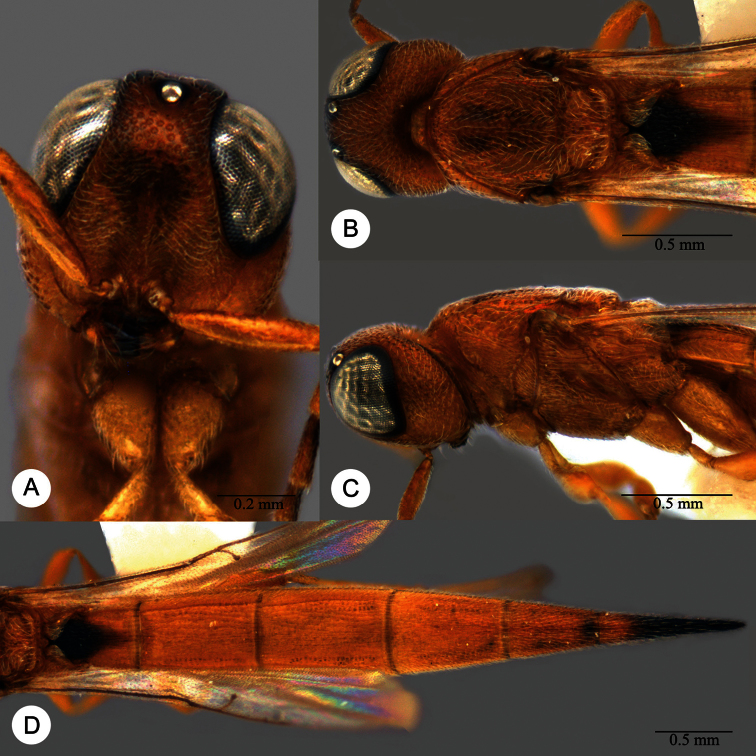
*Macroteleia rufa* Szelényi, female from Guangdong, Guangzhou, Tianlu Lake. **A** Head, anterior view **B** Head and mesosoma, dorsal view **C** Head and mesosoma, lateral view **D** Metasoma, dorsal view.

#### Distribution.

China (Guangdong, Hainan); Thailand. This species is also recorded in Egypt, Ukraine, Russia, Georgia and Tajikistan. Link to distribution map [http://hol.osu.edu/map-large.html?id=4863].

#### Material examined.

The *holotye* is deposited in Hungarian Nature History Museum, Budapest.

Other material. **CHINA**: 1 ♀, Guangdong, Zijin County, Linjiang Town, 23°39'N, 114°41'E, 1.VIII.2003, Jingxian Liu, SCAU 000044 (SCAU); 2 ♀, Guangdong, Xinfeng, Mt. Yunji, 24°04'N, 114°10'E, 19.VII.2003, Yanxia Song, SCAU 000045, 000046 (SCAU); 1 ♀, Guangdong, Mt. Nankun, 23°37.941'N, 113°50.182'E, 2.VII.2005, Zaifu Xu, SCAU 000047 (SCAU); 4 ♀, Guangdong, Guangzhou, Tianlu Lake, 23°13'N, 113°25'E, 6.X.2002, Zaifu Xu, SCAU 000048–000051 (SCAU); 1 ♀, Hainan, Mt. Yinggeling, 18°49'N, 109°11'E, 17–20.VII.2010, Huayan Chen, SCAU 000052 (SCAU). **THAILAND**: 1 ♀, Chiang Mai, Maerim, 10–12.III.2003, MT, R. A. Beaver, No. 26778 (RABC).

#### Comments.

The identification of this species is based on pictures of holotype of *Macroteleia rufa* (deposited in Hungarian Nature History Museum, Budapest) kindly provided by Dr. Ovidiu A. Popovici (University ‘Al. I. Cuza’ Iaşi) and his personal communication.

### Macroteleia salebrosa sp. n.

urn:lsid:zoobank.org:act:2016B64A-968B-4C39-A51F-DB4CDD750F75

http://species-id.net/wiki/Macroteleia_salebrosa

Plates 53 55

#### Description.

*Female*. Body length 4.76–5.90 mm (n=6).

*Color*. Body black; mandible reddish brown to black; palpi yellow; hind coxa dark brown to nearly black, remainder of legs yellow to light brown; A1–A6 yellow, remainder of antenna dark brown to black; fore wing hyaline.

*Head*. Transverse in dorsal view, 1.40–1.54× as wide as long, slightly wider than mesosoma; lateral ocellus contiguous with inner orbit of compound eye; POL 1.73–1.83× LOL; occipital carina interrupted medially; central keel well developed, extending onto interantennal process (Plate 54A); medial frons smooth dorsally, obliquely strigose ventrally; ventrolateral frons punctate rugose; frons below median ocellus and vertex punctate reticulate; gena dorsoventrally strigose below malar sulcus, otherwise punctate rugose; length of A3 1.07–1.15× length of A2.

*Mesosoma*. Cervical pronotal area densely punctate; dorsal pronotal area areolate; lateral pronotal area smooth dorsally, rugulose ventrally; netrion punctate rugulose; notaulus narrow, foveolate; middle lobe of mesoscutum densely punctate, becoming reticulate anteriorly; lateral lobes of mesoscutum moderately punctate throughout; mesoscutellum densely punctate throughout; metascutellum rectangular (Plate 54B), posterior margin slightly convex, extending to gap between propodeal lobes, irregularly areolate rugose; propodeum medially divided into two widely separated subtriangular lobes (Plate 54B), each side with rugose sculpture covered by dense, recumbent, white setae; upper mesepisternum with a row of fine longitudinal carinae below subalar pit; lower mesepisternum variably smooth to punctate rugulose; mesopleural depression smooth (Plate 54C); metapleuron smooth dorsally, punctate rugulose ventrally.

*Legs*. Robust; hind femur strongly swollen, 2.47–2.94× as long as its maximum width; hind tibia without spines over outer surface; hind basitarsus 4.50–5.00× as long as its maximum width.

*Wings*. Apex of fore wing extending beyond posterior margin of T5; R 1.50–1.89× as long as r-rs, R1 1.47–1.77× length of R.

*Metasoma*. Posterior margin of transverse sulcus on T2 slightly convex (Plate 54D); sublateral tergal carinae obscured by longitudinal surface striae; T1 punctate rugose anteriorly, densely striate posteriorly; T2–T5 densely longitudinally striate with numerous large delicate punctures in interstices; T6 punctate rugulose dorsally, longitudinally striate laterally, with scattered small punctures in interstices; length of T3 0.69–0.78× length of T6; T5 slightly longer than wide; S2–S6 densely longitudinally striate, with numerous punctures in interstices; prominent longitudinal median carina obscured by longitudinal surface striae.

*Male*. Differing from female as follows: body length 5.00 mm (n=2); A1–A5 yellow to brown, A6–A12 dark brown, becoming darker apically; propodeum continuous medially (Plate 55A), not divided into two separated lobes, posterior margin excavate medially, each side with several irregular longitudinal carinae medially, otherwise rugose; sublateral tergal carinae developed on T1–T2; T1 sparsely longitudinally striate, with scattered punctures in interstices anteriorly; T2–T6 longitudinally punctate rugulose; T7 transversely striate posteriorly; T6 slightly longer than wide; length of T6 2.56–2.86× length of T7; T7 transverse, apex truncate (Plate 55B); length of T7 0.83–0.84× length of S7; S2–S7 densely longitudinally striate, with numerous punctures in interstices; prominent longitudinal median carina present on S2–S4.

**Plate 53.**
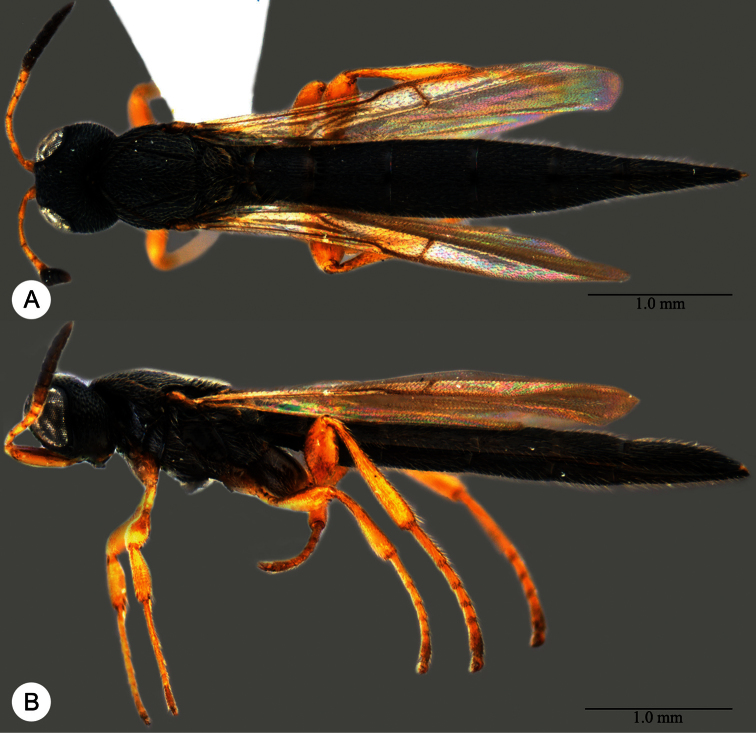
*Macroteleia salebrosa* sp. n., holotype, female. **A** Dorsal habitus **B** Lateral habitus.

**Plate 54.**
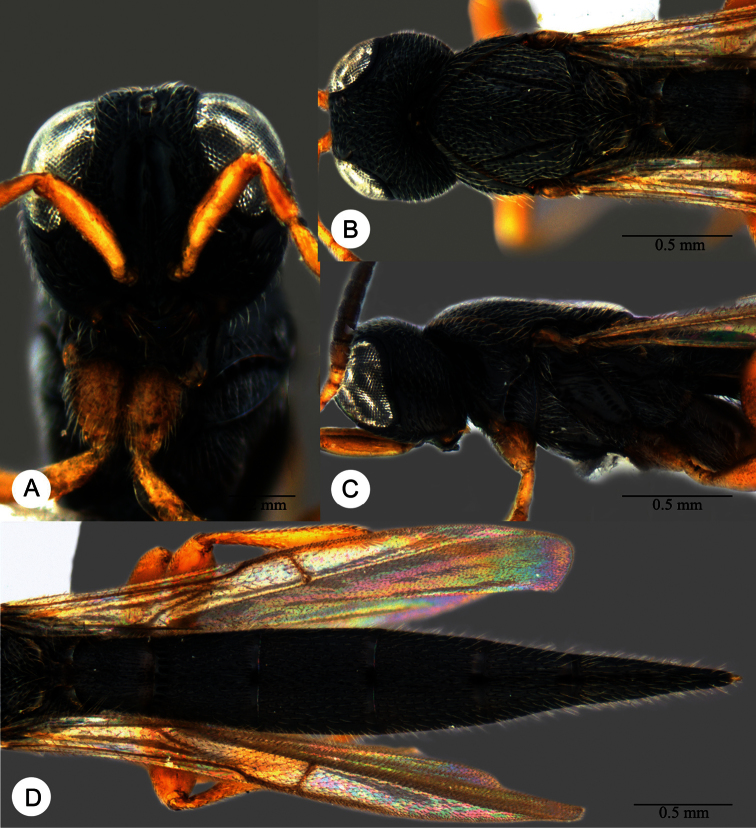
*Macroteleia salebrosa* sp. n., holotype, female. **A** Head, anterior view **B** Head and mesosoma, dorsal view **C** Head and mesosoma, lateral view **D** Metasoma, dorsal view.

**Plate 55.**
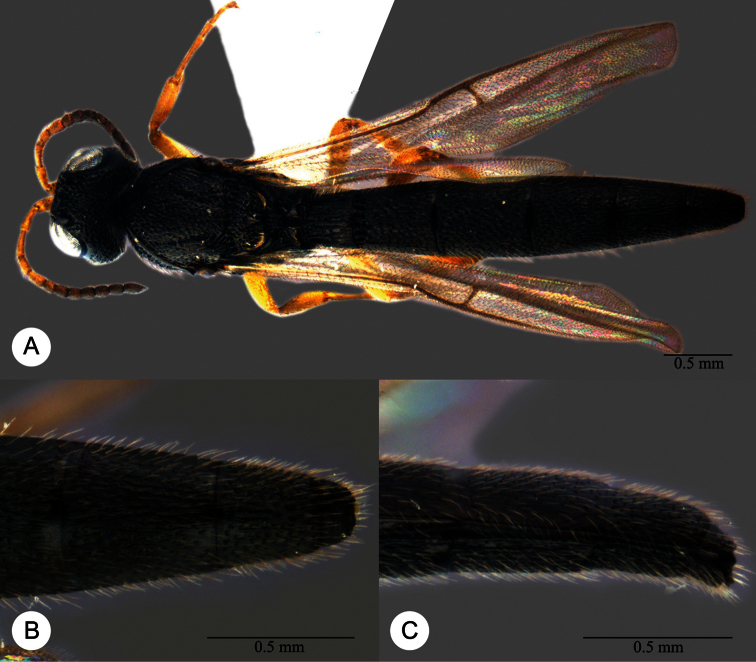
*Macroteleia salebrosa* sp. n., paratype, male. **A** Dorsal habitus **B** Apex of metasoma, dorsal view **C** Apex of metasoma, lateral view.

#### Etymology.

The name *salebrosa* refers to the coarse body sculpture of this species and is used as an adjective.

#### Distribution.

China (Zhejiang, Sichuan). Link to distribution map [http://hol.osu.edu/map-large.html?id=320506].

#### Material examined.

*Holotype*, ♀: **CHINA**: Zhejiang, Mt. Qingliangfeng, 9.VIII.2005, 30°04'N, 118°52'E, Hongying Zhang, SCAU 000016 (deposited in SCAU). *Paratypes*: 1 ♀, Zhejiang, Mt. Tianmu, Xianrending, 30°20.56'N, 119°26.03'E, 25–29.VII.2011, sweeping, Chengyuan Jin, SCAU 000017 (SCAU); 2 ♂, Zhejiang, Mt. Tianmu, Xianrending, 30°20.56'N, 119°26.03'E, 25–29.VII.2011, sweeping, Huayan Chen, SCAU 000018, 000019 (SCAU); 1 ♀, Zhejiang, Mt. Baishanzu, 27°45'N, 119°12'E, 1856m, 15.VIII.2003, Jingxian Liu, SCAU 000020 (SCAU); 3 ♀, Sichuan, Mt. Emei, 29°34'N, 103°26'E, 2.VIII.2006, Hongying Zhang, SCAU 000021–000023 (SCAU).

### Macroteleia semicircula sp. n.

urn:lsid:zoobank.org:act:5813FBA4-90E4-4A67-8ECF-A87E6EB7AF6E

http://species-id.net/wiki/Macroteleia_semicircula

Plates 56 58

#### Description.

*Female*. Body length 5.83–6.36 mm (n=4).

*Color*. Head yellow with upper frons and vertex dark brown to black; mesosoma variably yellow to dark brown; metasoma black; mandible dark brown; palpi yellow; legs yellow throughout; A1–A6 yellow, A7–A12 black; fore wing subhyaline.

*Head*. Transverse in dorsal view, 1.42–1.46× as wide as long, slightly wider than mesosoma; lateral ocellus contiguous with inner orbit of compound eye; POL 1.54–1.55× LOL; occipital carina continuous medially, coarsely and densely punctate; central keel well developed, extending onto interantennal process, slightly bifurcating dorsally (Plate 57A); medial frons smooth; ventrolateral frons punctate rugose; frons below median ocellus and vertex punctate reticulate; gena punctate rugose; length of A3 1.16–1.18× length of A2.

*Mesosoma*. Cervical pronotal area densely punctate; dorsal pronotal area areolate; lateral pronotal area anteriorly with several carinae subparallel to vertical epomial carina, otherwise smooth with sparse punctures; netrion finely punctate; notaulus deep, distinctly foveolate; mesoscutum densely punctate throughout; mesoscutellum sparsely punctate medially, densely punctate laterally; metascutellum semicircular (Plate 57B), extending to gap between propodeal lobes, irregularly areolate rugose; propodeum divided into two widely separated subtriangular lobes (Plate 57B), each side with rugose sculpture covered by dense, recumbent, white setae; upper mesepisternum with a row of robust longitudinal carinae below subalar pit; lower mesepisternum variably smooth to punctate rugulose; mesopleural depression smooth (Plate 57C); metapleuron smooth dorsally, punctate rugose ventrally.

*Legs*. Robust; hind femur strongly swollen, 2.31–2.46× as long as its maximum width; hind tibia without spines over outer surface; hind basitarsus 3.63–4.23× as long as its maximum width.

*Wings*. Apex of fore wing extending as far as mid-length of T5; R 1.38–1.60× as long as r-rs, R1 1.81–2.00× length of R.

*Metasoma*. Posterior margin of transverse sulcus on T2 strongly convex (Plate 57D); sublateral tergal carinae developed on T1–T3; T1 longitudinally punctate rugulose anteriorly, densely striate posteriorly; T2–T4 longitudinally punctate rugulose throughout; T5 –T6 finely punctate rugulose dorsally, densely longitudinally striate laterodorsally, with scattered small punctures in interstices; length of T3 0.63–0.66× length of T6; T5 slightly longer than wide; S2–S6 densely longitudinally striate, with coarse punctures in interstices; prominent longitudinal median carina present on S2–S4.

*Male*. Differing from female as follows: body length 4.12–5.56 mm (n=8); antenna yellow with A6–A11 brown; mesosoma variably brown to nearly black; fore wing slightly infuscate in basal half; propodeum continuous medially (Plate 58A), not divided into two separated lobes, posterior margin excavate medially, each side with several irregular longitudinal carinae medially, otherwise rugose; T1 sparsely longitudinally striate throughout; T7 largely smooth except finely rugulose posterolaterally; T6 wider than long or slightly longer than wide; length of T6 3.80–4.75× length of T7; T7 transverse, apex truncate (Plate 58B); length of T7 0.67–0.85× length of S7; S7 longitudinally punctate rugulose.

**Plate 56.**
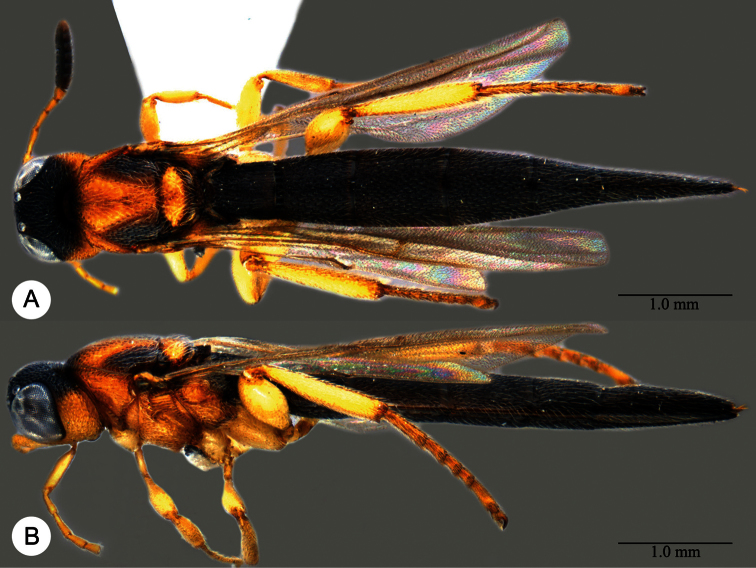
*Macroteleia semicircula* sp. n., holotype, female. **A** Dorsal habitus **B** Lateral habitus.

**Plate 57.**
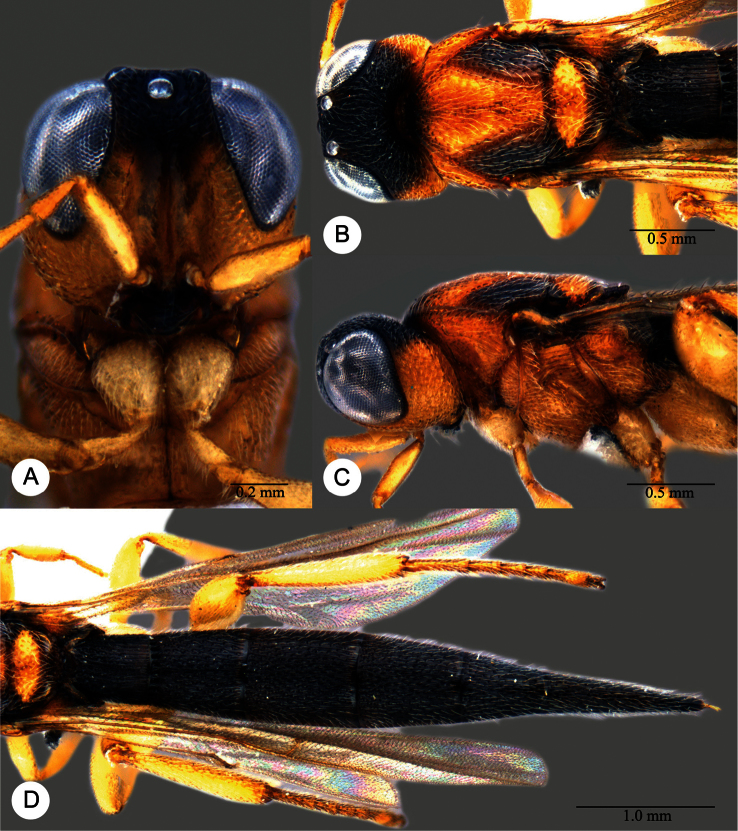
*Macroteleia semicircula* sp. n., holotype, female. **A** Head, anterior view **B** Head and mesosoma, dorsal view **C** Head and mesosoma, lateral view **D** Metasoma, dorsal view.

**Plate 58.**
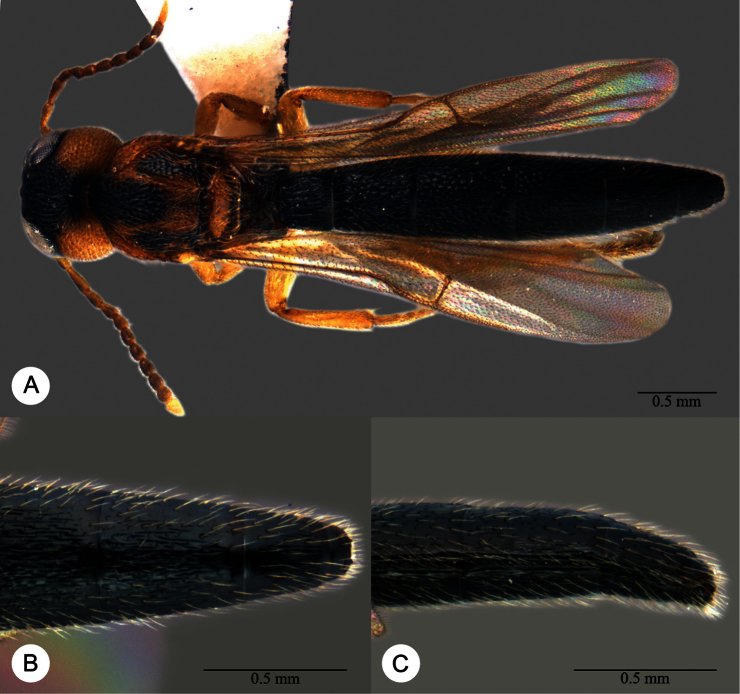
*Macroteleia semicircula* sp. n., paratype, male. **A** Dorsal habitus **B** Apex of metasoma, dorsal view **C** Apex of metasoma, lateral view.

#### Diagnosis.

*Macroteleia semicircula* shares the well-developed central keel and robust legs with *Macroteleia salebrosa* and *Macroteleia striatipleuron*, but can be distinguished by the semicircular metascutellum (rectangular in the latter two species).

#### Etymology.

The name *semicircula* refers to the semicircular metascutellum of this species and is used as a noun in apposition.

#### Distribution.

China (Guangdong, Hainan). Link to distribution map [http://hol.osu.edu/map-large.html?id=320507].

#### Material examined.

*Holotype*, ♀: **CHINA**: Guangdong, Nanling National Nature Reserve, 24°54'N, 113°00'E, 8–17.VIII.2010, sweeping, Huayan Chen, SCAU 000001 (deposited in SCAU). *Paratypes*: 2 ♂, Guangdong, Nanling National Nature Reserve, 24°54'N, 113°00'E, 9–18.VII.2004, Juanjuan Ma, SCAU 000002, 000003 (SCAU); 1 ♀, Hainan, Bawangling National Nature Reserve, 19°07'N, 109°03'E, 21.X.2007, Jiemin Yao, SCAU 000004 (SCAU); 1 ♀ + 1 ♂, Hainan, Bawangling National Nature Reserve, 19°07'N, 109°03'E, 21.V.2007, Jingxian Liu, SCAU 000005, 000006 (SCAU); 1 ♂, Hainan, Wuzhishan National Nature Reserve, 18°51'N, 109°39'E, 16–18.V.2007, Liqiong Weng, SCAU 000007 (SCAU); 1 ♂, Hainan, Wuzhishan National Nature Reserve, 16–18.V.2007, 18°51'N, 109°39'E, Jingxian Liu, SCAU 000008 (SCAU); 1 ♀, Hainan, Mt. Yinggeling, 18°49'N, 109°11'E, 24–25.X.2007, Jingxian Liu, SCAU 000009 (SCAU); 1 ♂, Hainan, Mt. Yinggeling, 18°49'N, 109°11'E, 22–25.V.2007, Jie Zeng, SCAU 000010 (SCAU); 2 ♂, Hainan, Mt. Yinggeling, 18°49'N, 109°11'E, 28.X.2007, Liqiong Weng, SCAU 000011, 000012 (SCAU).

### Macroteleia spinitibia sp. n.

urn:lsid:zoobank.org:act:86880BD0-AC42-48C1-A096-D6A1B191C7EE

http://species-id.net/wiki/Macroteleia_spinitibia

Plates 59 61

#### Description.

*Male*. Body length 6.08–6.17 mm (n=2).

*Color*. Head yellow; mesosoma variably yellow to brown; metasoma black; mandible dark brown; palpi yellow; legs yellow with hind tarsi brown; A1 and A12 yellow, A2–A7 brown, A8–A11 dark brown; fore wing hyaline with medial longitudinal infuscate streak extending to near apex of wing (Plate 60D).

*Head*. Transverse in dorsal view, 1.38–1.40× as wide as long, slightly wider than mesosoma; lateral ocellus contiguous with inner orbit of compound eye; POL 1.81–1.88× LOL; occipital carina continuous medially, densely punctate; central keel well developed (Plate 60A), extending onto interantennal process; medial frons largely smooth, finely strigose dorsally; remainder of frons punctate rugulose; vertex punctate reticulate, narrow interspaces with fine microsculpture; gena punctate rugulose; length of A3 1.04× length of A2.

*Mesosoma*. Cervical pronotal area punctate rugulose; dorsal pronotal area areolate; lateral pronotal area smooth anteriorly, densely punctate posteriorly; netrion rugulose; notaulus deep, distinctly foveolate; middle lobe of mesoscutum punctate reticulate throughout, narrow interspaces in part with fine microsculpture; lateral lobes of mesoscutum sparsely punctate throughout, interspaces with fine microsculpture; mesoscutellum densely punctate throughout; metascutellum transverse (Plate 60B), posterior margin concave medially, irregularly rugose; propodeum continuous medially, not divided into two separated lobes (Plate 60B), posterior margin convex, each side with several irregular longitudinal carinae medially, otherwise rugose; upper mesepisternum with a row of longitudinal fine striae below subalar pit; lower mesepisternum punctate rugulose; mesopleural depression longitudinally striate (Plate 60C); metapleuron longitudinally striate throughout.

*Legs*. Robust; hind femur strongly swollen, 2.86× as long as its maximum width; hind tibia with numerous semi–erect, yellow spines over outer surface (Plate 61D); hind basitarsus 6.53–6.67× as long as its maximum width.

*Wings*. Apex of fore wing extending as far as basal third of T5; R 1.50–1.53× as long as r-rs, R1 1.04–1.53× length of R.

*Metasoma*. Posterior margin of transverse sulcus on T2 straight (Plate 61A); sublateral tergal carinae well developed on T1–T2; T1 sparsely longitudinally striate, with delicate punctures in interstices; T2–T6 densely longitudinally striate, with numerous delicate punctures in interstices; T7 finely rugulose posterolaterally; T6 slightly longer than wide; length of T6 4.00–4.55× length of T7; T7 transverse, apex truncate (Plate 61B); length of T7 0.61–0.71× length of S7; S2–S6 densely longitudinally striate, with numerous punctures in interstices; S7 densely punctate; prominent longitudinal median carina present on S2–S6.

*Female*. Unknown.

**Plate 59.**
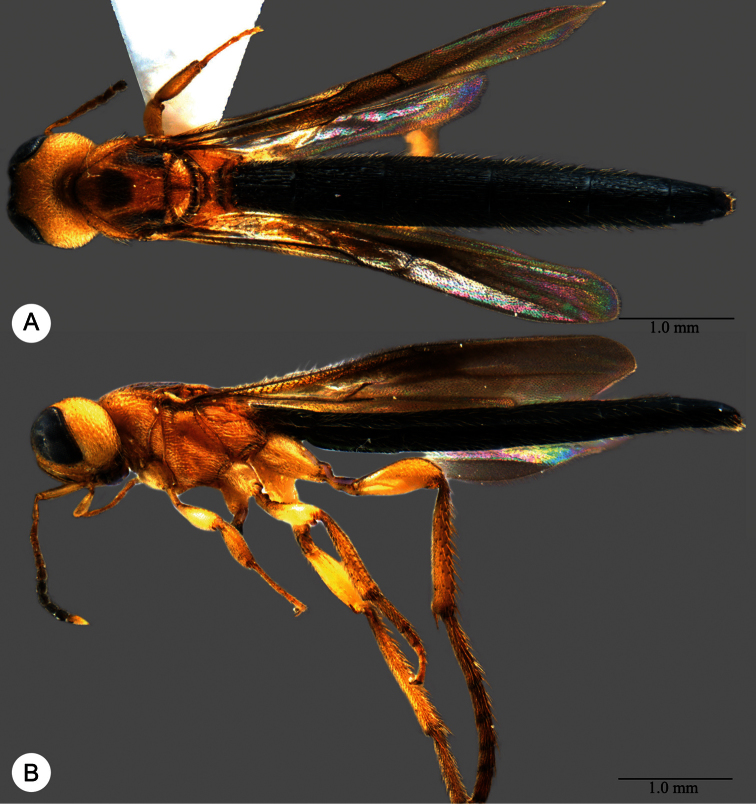
*Macroteleia spinitibia* sp. n., holotype, male. **A** Dorsal habitus **B** Lateral habitus.

**Plate 60.**
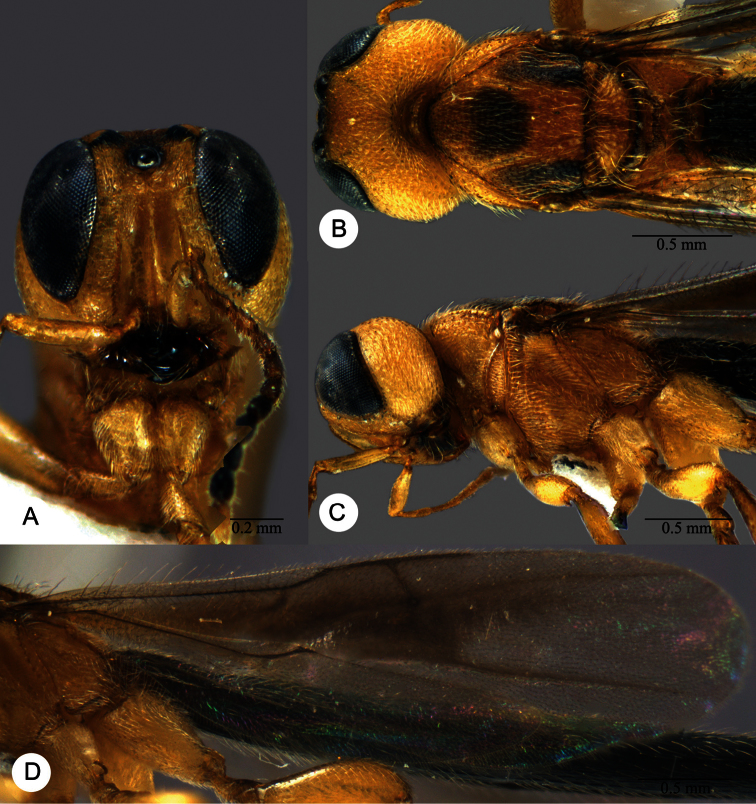
*Macroteleia spinitibia* sp. n., holotype, male. **A** Head, anterior view **B** Head and mesosoma, dorsal view **C** Head and mesosoma, lateral view **D** Fore and hind wing.

**Plate 61.**
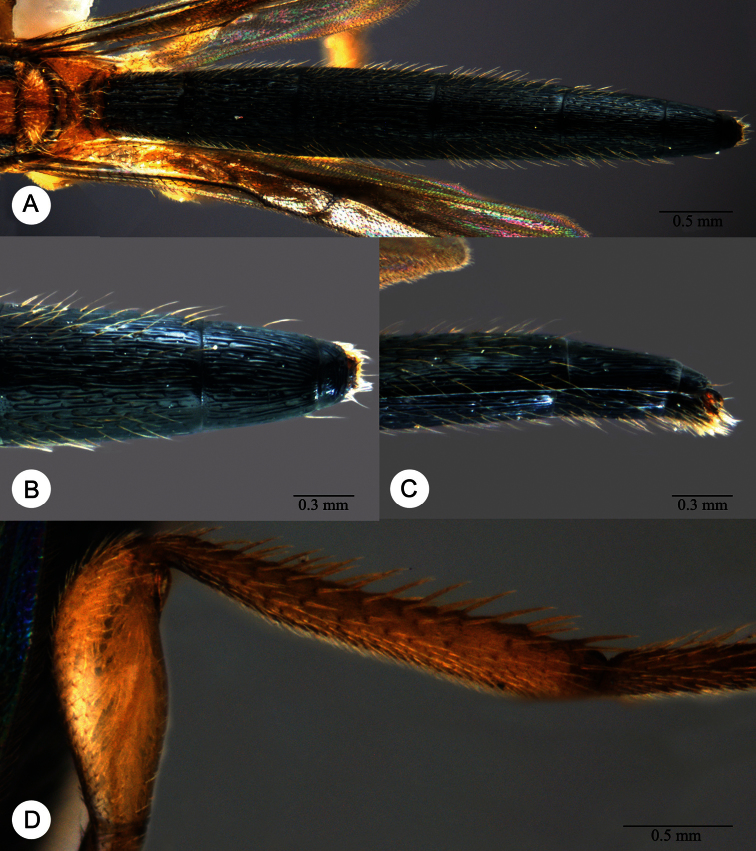
*Macroteleia spinitibia* sp. n., holotype, male. **A** Metasoma, dorsal view **B** Apex of metasoma, dorsal view **C** Apex of metasoma, lateral view **D** Hind femur and tibia.

#### Diagnosis.

The combination of infuscate fore wing, short R1 and spinulate hind tibia renders this species rather straightforward to identify.

#### Etymology.

The name *spinitibia* refers to hind tibia with numerous semi-erect spines over outer surface in this species. The epithet is used as a noun in apposition.

#### Distribution.

China (Hainan). Link to distribution map [http://hol.osu.edu/map-large.html?id=320508].

#### Material Examined.

*Holotype*, ♂: **CHINA**: Hainan, Bawangling National Nature Reserve, 19°07'N, 109°03'E, 7–11.VII.2006, Jingxian Liu, SCAU 000014 (deposited in SCAU). *Paratype*: 1 ♂, Hainan, Mt. Yinggeling, 18°49'N, 109°11'E, 16.XI.2008, Manman Wang, SCAU 000015 (SCAU).

### Macroteleia striatipleuron sp. n.

urn:lsid:zoobank.org:act:53384A4A-28B5-4F08-9CC6-EA8CBBBB5260

http://species-id.net/wiki/Macroteleia_striatipleuron

Plates 62 63

#### Description.

*Female*. Body length 6.50 mm.

*Color*. Body black; mandible dark brown; palpi yellow; legs yellow with hind tarsi brown; A1 yellow, A2-A4 brown, A5 dark brown, remainder of antenna black; fore wing subhyaline with medial longitudinal infuscate streak in basal half.

*Head*. Transverse in dorsal view, width 1.46× length, as wide as mesosoma; OOL short, 0.25× minimum ocellar width; POL 1.73× LOL; occipital carina continuous medially, coarsely and densely punctate; central keel well developed, extending onto interantennal process, slightly bifurcating dorsally (Plate 2A); medial frons smooth dorsally, obliquely strigose ventrally; remainder of frons and vertex punctate reticulate; gena punctate rugose; length of A3 1.03× length of A2.

*Mesosoma*. Cervical pronotal area punctate reticulate; dorsal pronotal area areolate; lateral pronotal area anteriorly with several carinae subparallel to vertical epomial carina, otherwise smooth with sparse punctures; netrion finely punctate rugulose; notaulus deep, distinctly foveolate; middle lobe of mesoscutum densely punctate, becoming punctate reticulate anteriorly and posteriorly; lateral lobes of mesoscutum densely punctate throughout; mesoscutellum densely punctate throughout; metascutellum rectangular (Plate 63A), posterior margin convex, extending to gap between propodeal lobes, irregularly areolate rugose; propodeum medially divided into two widely separated subtriangular lobes (Plate 63B), each side with rugose sculpture covered by dense, recumbent, white setae; upper mesepisternum with a row of robust longitudinal carinae below subalar pit; lower mesepisternum densely punctate; mesopleural depression longitudinally striate (Plate 63B); metapleuron punctate rugose throughout.

*Legs*. Robust; hind femur strongly swollen, 2.70× as long as its maximum width; hind tibia without spines over outer surface; hind basitarsus 3.75× as long as its maximum width.

*Wings*. Apex of fore wing extending as far as mid-length of T6; R 1.36 × as long as r-rs, R1 1.89× length of R.

*Metasoma*. Posterior margin of transverse sulcus on T2 slightly convex (Plate 63C); sublateral tergal carinae developed on T1–T2; T1 longitudinally punctate rugose anteriorly, densely striate posteriorly; T2–T5 longitudinally punctate rugulose throughout; T6 longitudinally striate throughout, with scattered small punctures in interstices; length of T3 0.81× length of T6; T5 slightly wider than long; S2–S6 densely longitudinally striate, with coarse punctures in interstices; prominent longitudinal median carina strongly developed on S2–S4.

*Male*. Unknown.

**Plate 62.**
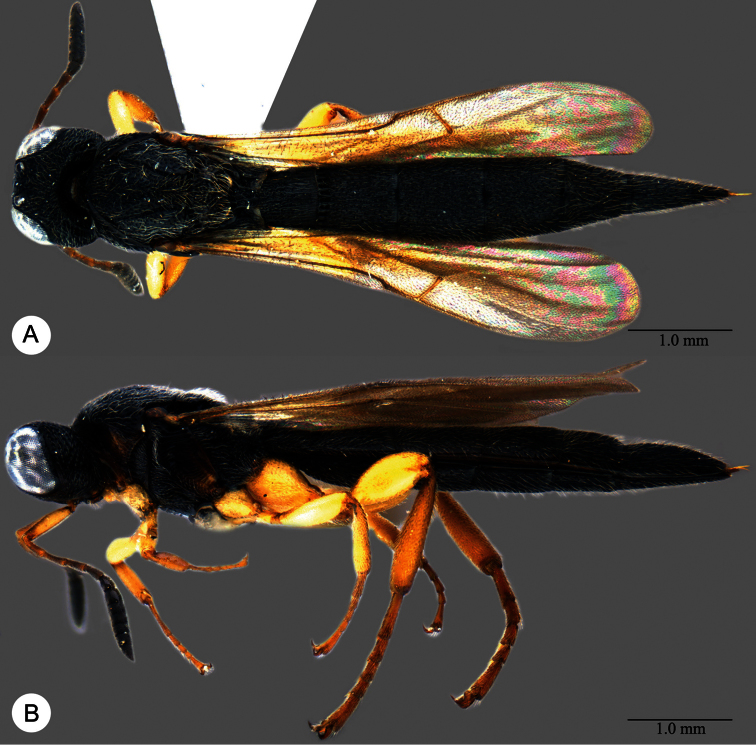
*Macroteleia striatipleuron* sp. n., holotype, female. **A** Dorsal habitus **B** Lateral habitus.

**Plate 63.**
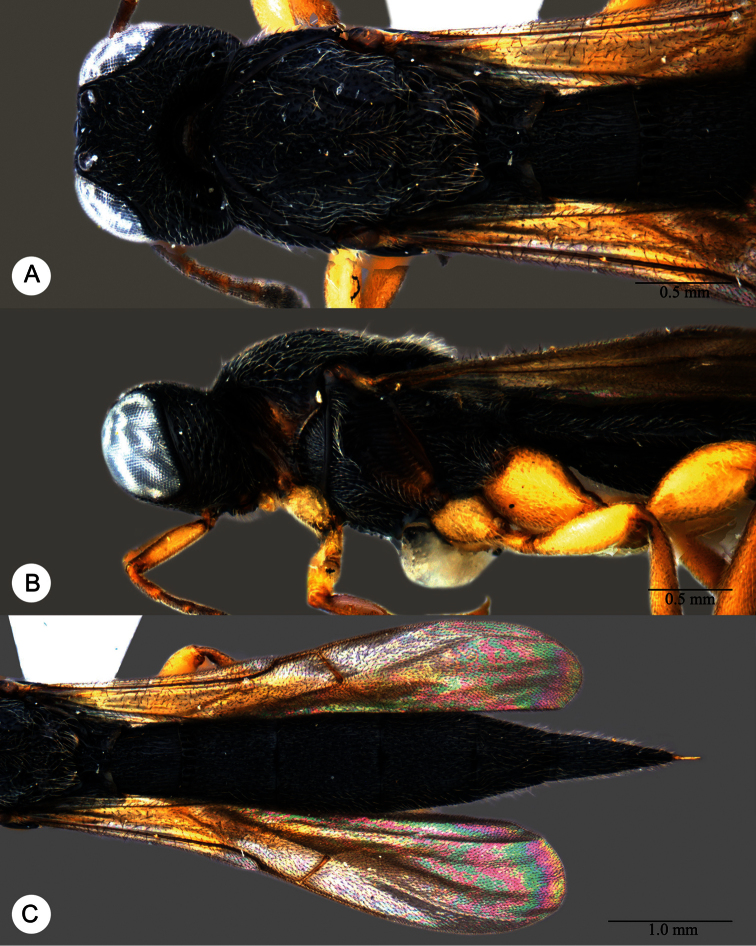
*Macroteleia striatipleuron* sp. n., holotype, female. **A** Head and mesosoma, dorsal view **B** Head and mesosoma, lateral view **C** Metasoma, dorsal view.

#### Diagnosis.

*Macroteleia striatipleuron* shares the well-developed central keel,rectangular metascutellum and robust legs with *Macroteleia salebrosa*, but can be distinguished by the medially continuous occipital carina (interrupted medially in *Macroteleia salebrosa*); longitudinally striate mesopleural depression (smooth in *Macroteleia salebrosa*); and yellow hind coxa (dark brown to nearly black in *Macroteleia salebrosa*).

#### Etymology.

The name *striatipleuron* refers to the longitudinal striae on mesopleural depression of this species and is used as a noun in apposition.

#### Distribution.

China (Guangdong). Link to distribution map [http://hol.osu.edu/map-large.html?id=320509].

#### Material examined.

*Holotype*, ♀: **CHINA**: Guangdong, Nanling National Nature Reserve, 24°54'N, 113°00'E, 8–17.VIII.2010, sweeping, Huayan Chen, SCAU 000013 (deposited in SCAU).

### Macroteleia striativentris

Crawford

http://species-id.net/wiki/Macroteleia_striativentris

Plates 64 69

Macroteleia striativentris Crawford 1910: 126 (original description); Kieffer 1913b: 323 (description, keyed); Kieffer 1914b: 298 (keyed); Kieffer 1926: 521, 527 (description, keyed); Baltazar 1966: 184 (listed, synonymy); Masner and Muesebeck 1968: 40 (type information); Lê 2000: 53, 63 (description, keyed).

#### Description.

*Female*. Body length 4.06–6.34 mm (n=20).

*Color*. Head and mesosoma black, metasoma dark brown to black; mandible dark brown; palpi yellow; legs pale brown throughout; A1 brown, A2–A5 dark brown, remainder of antenna black; fore wing hyaline.

*Head*. Transverse in dorsal view, 1.28–1.48× as wide as long, slightly wider than mesosoma; OOL short, 0.29–0.33× minimum ocellar width; POL 1.25–1.33× LOL; occipital carina interrupted medially; central keel absent or weakly developed above interantennal process (Plates 66C, 68A); medial frons punctate rugulose ventrally, irregularly smooth dorsally; ventrolateral frons punctate rugose; frons below median ocellus densely punctate; vertex densely punctate; gena punctate rugose; length of A3 0.83–0.90× length of A2.

*Mesosoma*. Cervical pronotal area densely punctate; dorsal pronotal area areolate; lateral pronotal area smooth anteriorly, punctate rugulose posteriorly; netrion punctate rugose; notaulus narrow, irregularly foveolate; middle lobe of mesoscutum densely punctate, becoming denser anteriorly and posteriorly; lateral lobes of mesoscutum densely punctate throughout; mesoscutellum densely punctate throughout; metascutellum distinctly transverse (Plates 65B, 66D, 68B), posterior margin slightly pointed medially, longitudinally carinate; propodeum continuous medially (Plates 65B, 66D, 68B), not divided into two separated lobes, posterior margin narrowly notched medially, each side with several irregular longitudinal carinae medially, otherwise punctate rugulose, covered by dense, recumbent, white setae; upper mesepisternum with a row of robust longitudinal carinae below subalar pit; lower mesepisternum punctate rugulose; mesopleural depression smooth (Plates 65C, 66E, 68C); metapleuron longitudinally striate throughout.

*Legs*. Slender; hind femur weakly swollen, 4.12–4.87× as long as its maximum width; hind tibia without spines over outer surface; hind basitarsus 10.60–12.50× as long as its maximum width.

*Wings*. Apex of fore wing extending as far as mid-length of T4; R 1.33–2.08× as long as r-rs, R1 1.90–2.38× length of R.

*Metasoma*. Posterior margin of transverse sulcus on T2 straight (Plates 65D, 66F, 68D); sublateral tergal carinae developed on T1–T3; T1 densely longitudinally striate medially, with scattered punctures in interstices anteriorly, punctate rugulose laterally; T2–T4 densely longitudinally striate medially, with delicate punctures in interstices, punctate rugulose laterally; T5–T6 finely punctate dorsally, densely longitudinally striate laterally, with scattered small punctures in interstices; length of T3 0.72–0.97× length of T6; T5 distinctly longer than wide; S2–S3 sparsely longitudinally striate, with finely punctate rugulose interstices; S4–S6 longitudinally striate, with finely punctate interstices; prominent longitudinal median carina present on S2–S5.

*Male*. Differing from female as follows: body length 5.22–5.57 mm (n=9); head brown to dark brown; mesosoma black; metasoma variably brown to black; A1 brown, remainder of antenna dark brown to black; T1 densely longitudinally striate medially, with rugulose sculpture in interstices anteriorly, punctate rugulose laterally; T2–T3 densely longitudinally striate medially, with delicate punctures in interstices, punctate rugulose laterally; T4–T7 longitudinally punctate rugulose; T6 distinctly longer than wide; length of T6 1.20–1.41× length of T7; T7 subtriangular, apex pointed (Plate 69B); length of T7 2.21–2.50× length of S7; S2–S5 longitudinally striate, with delicate punctures in interstices; S6–S7 longitudinally punctate rugulose.

**Plate 64.**
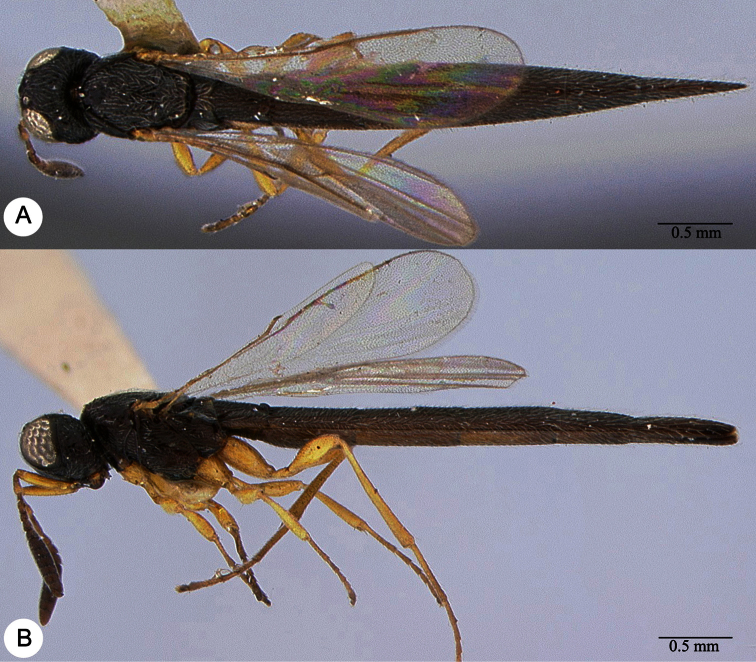
*Macroteleia striativentris* Crawford, holotype, female. **A** Dorsal habitus **B** Lateral habitus.

**Plate 65.**
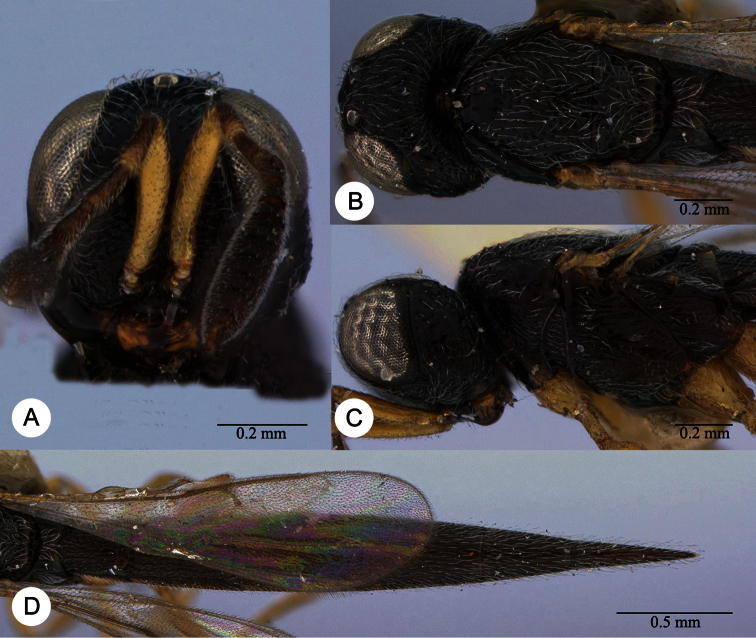
*Macroteleia striativentris* Crawford, holotype, female. **A** Head, anterior view **B** Head and mesosoma, dorsal view **C** Head and mesosoma, lateral view **D** Metasoma and fore wing, dorsal view.

**Plate 66.**
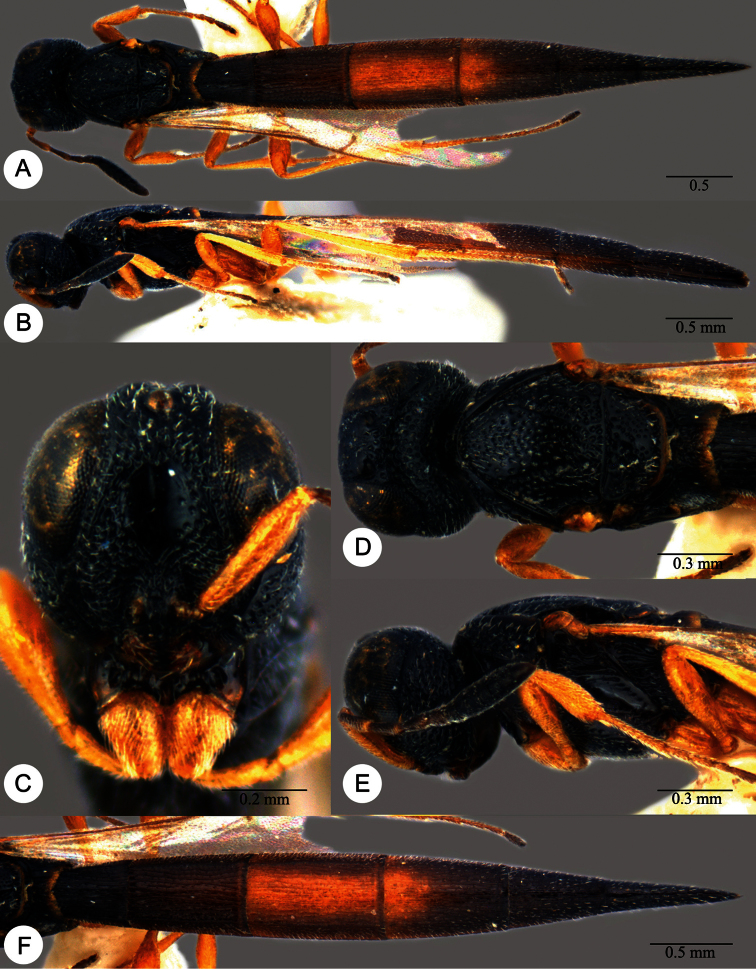
*Macroteleia fugacious* Kozlov & Lê, paratype, female. **A** Dorsal habitus **B** Lateral habitus **C** Head, anterior view **D** Head and mesosoma, dorsal view **E** Head and mesosoma, lateral view **F** Metasoma, dorsal view.

**Plate 67.**
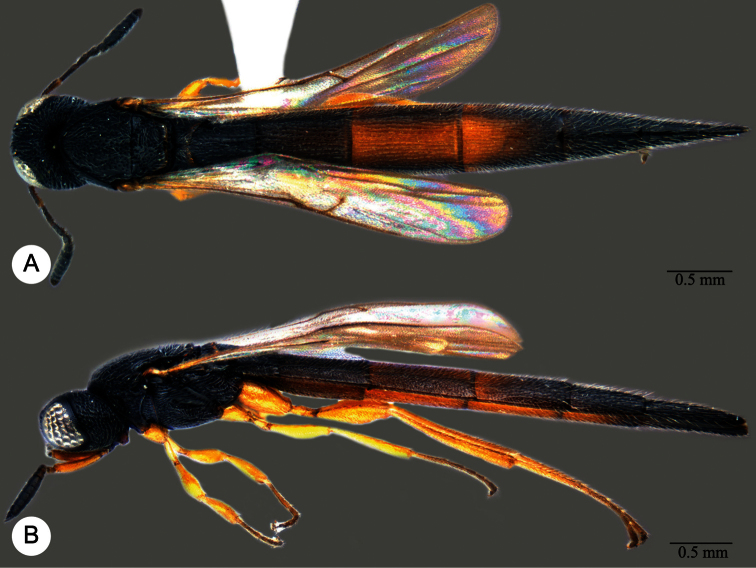
*Macroteleia striativentris* Crawford, female from Guangdong, Chebaling National Nature Reserve. **A** Dorsal habitus **B** Lateral habitus.

**Plate 68.**
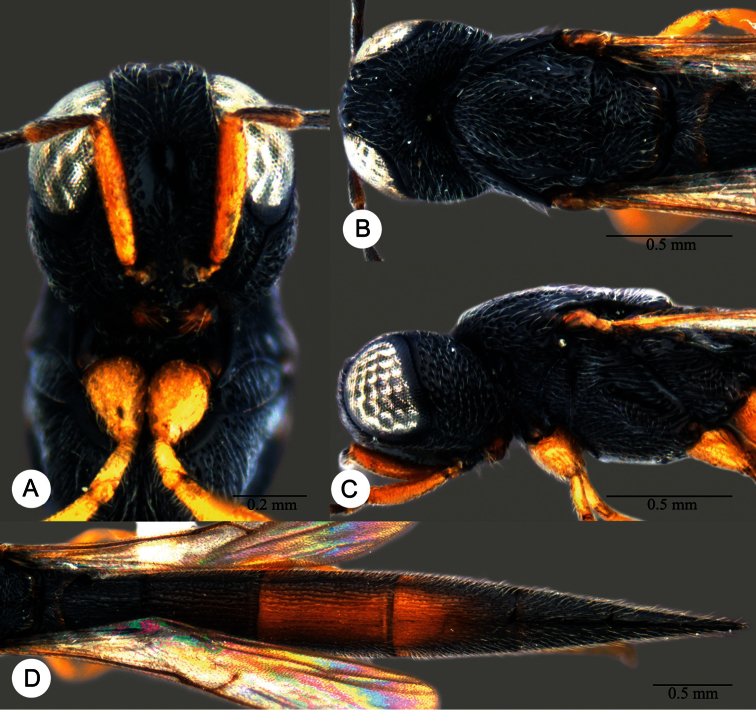
*Macroteleia striativentris* Crawford, female from Guangdong, Chebaling National Nature Reserve. **A** Head, anterior view **B** Head and mesosoma, dorsal view **C** Head and mesosoma, lateral view **D** Metasoma, dorsal view.

**Plate 69.**
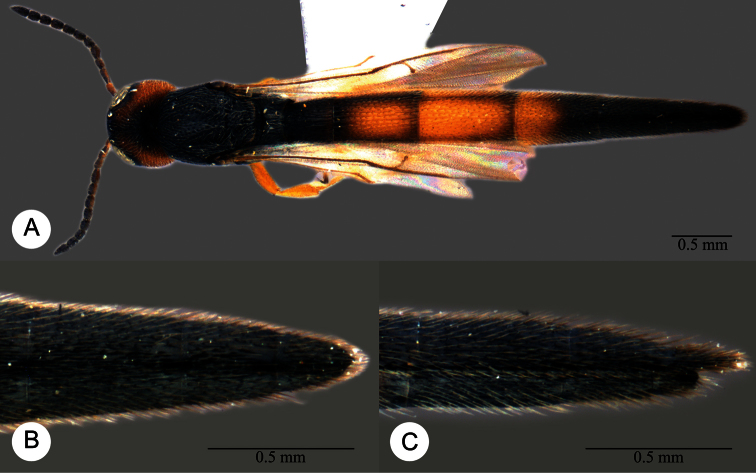
*Macroteleia striativentris* Crawford, male from Guangdong, Guangzhou, Tianlu Lake. **A** Dorsal habitus **B** Apex of metasoma, dorsal view **C** Apex of metasoma, lateral view.

#### Distribution.

China (Guangdong, Hainan, Yunnan); Vietnam; Thailand; Philippines. Link to distribution map [http://hol.osu.edu/map-large.html?id=4873].

#### Material examined.

*Lectotype*, ♀, **PHILIPPINES**: “Manila PI”, “Robt Brown Collector”, “*Macroteleia striativentris* ♀ Type Crawford”, “Lectotype ♀ *Macroteleia striativentris* Crawford by L. Masner, 1967”, “Type No 12896 U.S.N.M.” (deposited in USNM).

#### Other material.

**CHINA**: 1 ♀, Guangdong, Chebaling National Nature Reserve, 24°43'N, 114°14'E, 25.V.2002, Jingxian Liu, SCAU 000086 (SCAU); 1 ♂, Guangdong, Chebaling National Nature Reserve, 24°43'N, 114°14'E, 10.VII.2003, Zaifu Xu, SCAU 000087 (SCAU); 2 ♀, Guangdong, Fengkai County, Mt. Qiancengfeng, 23°28'N, 111°48'E, VIII.2003, Jingxian Liu, SCAU 000088, 000089 (SCAU); 3 ♀, Guangdong, Zijin County, Linjiang Town, 23°39'N, 114°41'E, 1.VIII.2003, Jingxian Liu, SCAU 000090–000092 (SCAU); 1 ♀, Guangdong, Mt. Tongleda, 23°10'N, 111°25'E, 12.VIII.2003, Jujian Chen, SCAU 000093 (SCAU); 4 ♀, Guangdong, Zhaoqing, Xiwanggu, 23°13'N, 112°31'E, 2–6.VIII.2010, sweeping, Huayan Chen, SCAU 000094–000097 (SCAU); 3 ♀, Guangdong, Zhaoqing, Xiwanggu, 23°13'N, 112°31'E, 2–6.VIII.2010, yellow pan trap, Huayan Chen, SCAU 000098–000100 (SCAU); 1 ♀, Guangdong, Guangzhou, Tianlu Lake, 23°13'N, 113°25'E, 24.VI.2003, Jingxian Liu, SCAU 000101 (SCAU); 8 ♂, Guangdong, Guangzhou, Tianlu Lake, 23°13'N, 113°25'E, 6.X.2002, Zaifu Xu, SCAU 000102–000109 (SCAU); 1 ♀, Hainan, Wuzhishan National Nature Reserve, 18°51'N, 109°39'E, 16–18.V.2007, Jingxian Liu, SCAU 000110 (SCAU); 1 ♀, Hainan, Mt. Yinggeling, 18°49'N, 109°11'E, 18.X.2007, Jingxian Liu, SCAU 000111 (SCAU); 3 ♀, Hainan, Mt. Yinggeling, 18°49´N, 109°11'E, 17–20.VII.2010, Huayan Chen, SCAU 000112–000114 (SCAU); 1 ♀, Yunnan, Lushui County, Shangjiang Town, 25°39'N, 98°52'E, 19.VII.2006, Jie Zeng, SCAU 000115 (SCAU); 2 ♀, Yunnan, Gaoligongshan National Nature Reserve, 24°49'N, 98°46'E, 1.VIII.2005, Juanjuan Ma, SCAU 000116, 000117 (SCAU). **THAILAND**: 1 ♀, Chiang Mai: Maerim, 10.IV.2003, FIT, R. A. Beaver, No. 27042 (RABC); 1 ♀, Chiang Mai: Maerim, 7.X.2003, FIT, R. A. Beaver, No. 28207 (RABC). **VIETNAM**: “Bac Thai, Phu Luong, Quan Chu, 23.IV.1986, A. Sarkov”, “Paratypus, *Macroteleia fugacious* sp. n.” (IEBR).

#### Comments.

*Macroteleia fugacious* was described by Lê (2000) based on five females and three males. He stated the holotype (female) and four paratypes (two females and two males) were deposited in the Institute of Ecology and Biological Resources (IEBR). Two other paratypes are deposited in the Zoological Institute in St. Petersburg. However, the second author found that the holotype of *Macroteleia fugacious* is housed in the collection of the Zoological Institude in St. Petersburg. When examining the types of Lê’s species of Scelioninae in IEBR, the first author only found one female and one male specimens labeled as paratypes of *Macroteleia fugacious*. The two paratypes, unfortunately, are different species: the male paratype belongs to *Macroteleia dolichopa*, and the female paratype belongs to *Macroteleia striativentris*. We were unable to examine the holotype of *Macroteleia fugacious* in this study, and therefore the identity of *Macroteleia fugacious* is uncertain.

## Acknowledgements

Thanks to Dr Lê Xuan Hue and Dr Khuat Dang Long (IEBR) for the loan of Vietnamese *Macroteleia* types; to Dr Nguyen Duc Tung (Hanoi University of Agriculture) for his kind help during the study of the Vietnamese *Macroteleia* species; to Dr R.A. Beaver (Thailand) for the loan of his specimens; to Dr Ovidiu A. Popovici (University ‘Al. I. Cuza’ Iaşi) for discussion; to M. Buffington and T. Nuhn for loans of the types deposited in Washington; to Prof. Junhua He and Prof. Xuexin Chen of Zhejiang University for their generous loan of their specimens of *Macroteleia*; and to Jingxian Liu, Liqiong Weng, Long Hu, Bin Xiao, Huiting Chen, Yali Cai, Chundan Hong, Jie Zeng, Juanjuan Ma, Jiemin Yao, and Chengyuan Jin for their help during field trips. This material is based upon work supported in part by the National Basic Research Program of China (No. 2013CB127600) and the National Science Foundation of USA under grant No. DEB-0614764 to N.F. Johnson and A.D. Austin.

## Supplementary Material

XML Treatment for Macroteleia

XML Treatment for Macroteleia boriviliensis

XML Treatment for Macroteleia carinigena

XML Treatment for Macroteleia crawfordi

XML Treatment for Macroteleia dolichopa

XML Treatment for Macroteleia emarginata

XML Treatment for Macroteleia flava

XML Treatment for Macroteleia gracilis

XML Treatment for Macroteleia indica

XML Treatment for Macroteleia lamba

XML Treatment for Macroteleia livingstoni

XML Treatment for Macroteleia peliades

XML Treatment for Macroteleia rufa

XML Treatment for Macroteleia salebrosa

XML Treatment for Macroteleia semicircula

XML Treatment for Macroteleia spinitibia

XML Treatment for Macroteleia striatipleuron

XML Treatment for Macroteleia striativentris

## Figures and Tables

**Table:** URI Table matching terms and concepts used in this revision with the Hymenoptera Anatomy Ontology database.<br/>

	A1	http://purl.obolibrary.org/obo/HAO_0000908
	A2	http://purl.obolibrary.org/obo/HAO_0000706
	A3	http://purl.obolibrary.org/obo/HAO_0001148
	A7	http://purl.obolibrary.org/obo/HAO_0001885
	A12	http://purl.obolibrary.org/obo/HAO_0001884
	antenna	http://purl.obolibrary.org/obo/HAO_0000101
	antennomere	http://purl.obolibrary.org/obo/HAO_0000107
	area	http://purl.obolibrary.org/obo/HAO_0000146
	body	http://purl.obolibrary.org/obo/HAO_0000182
	carina	http://purl.obolibrary.org/obo/HAO_0000188
	central keel	http://purl.obolibrary.org/obo/HAO_0000109
cpa	cervical pronotal area	http://purl.obolibrary.org/obo/HAO_0000194
	clava	http://purl.obolibrary.org/obo/HAO_0000203
	clypeus	http://purl.obolibrary.org/obo/HAO_0000212
	compound eye	http://purl.obolibrary.org/obo/HAO_0000217
	coxa	http://purl.obolibrary.org/obo/HAO_0000228
	depression	http://purl.obolibrary.org/obo/HAO_0000241
dpa	dorsal pronotal area	http://purl.obolibrary.org/obo/HAO_0000267
	egg	http://purl.obolibrary.org/obo/HAO_0000286
	epomial carina	http://purl.obolibrary.org/obo/HAO_0000307
	eye	http://purl.obolibrary.org/obo/HAO_0000217
	femur	http://purl.obolibrary.org/obo/HAO_0000327
	fore wing	http://purl.obolibrary.org/obo/HAO_0000351
	frons	http://purl.obolibrary.org/obo/HAO_0001523
	gena	http://purl.obolibrary.org/obo/HAO_0000371
	head	http://purl.obolibrary.org/obo/HAO_0000397
	hind coxa	http://purl.obolibrary.org/obo/HAO_0000587
	hind tibia	http://purl.obolibrary.org/obo/HAO_0000631
	hind wing	http://purl.obolibrary.org/obo/HAO_0000400
	inner orbit	http://purl.obolibrary.org/obo/HAO_0000419
	interantennal process	http://purl.obolibrary.org/obo/HAO_0000422
	lateral lobe of mesoscutum	http://purl.obolibrary.org/obo/HAO_0000466
	lateral ocellus	http://purl.obolibrary.org/obo/HAO_0000481
LOL	lateral ocellar line	http://purl.obolibrary.org/obo/HAO_0000480
lpa	lateral pronotal area	http://purl.obolibrary.org/obo/HAO_0000483
	malar sulcus	http://purl.obolibrary.org/obo/HAO_0000504
	mandible	http://purl.obolibrary.org/obo/HAO_0000506
	margin	http://purl.obolibrary.org/obo/HAO_0000510
	mesepisternum	http://purl.obolibrary.org/obo/HAO_0001872
med	mesopleural depression	http://purl.obolibrary.org/obo/HAO_0000326
	mesopleuron	http://purl.obolibrary.org/obo/HAO_0000566
	mesoscutellum	http://purl.obolibrary.org/obo/HAO_0000574
	mesoscutum	http://purl.obolibrary.org/obo/HAO_0001490
	mesosoma	http://purl.obolibrary.org/obo/HAO_0000576
	metapleuron	http://purl.obolibrary.org/obo/HAO_0000621
	metascutellum	http://purl.obolibrary.org/obo/HAO_0000625
	metasoma	http://purl.obolibrary.org/obo/HAO_0000626
	midlobe of mesoscutum	http://purl.obolibrary.org/obo/HAO_0000520
	netrion	http://purl.obolibrary.org/obo/HAO_0000644
	notauli (notaulus)	http://purl.obolibrary.org/obo/HAO_0000647
	occipital carina	http://purl.obolibrary.org/obo/HAO_0000653
	ocellus	http://purl.obolibrary.org/obo/HAO_0000661
ot	ocellar triangle	http://purl.obolibrary.org/obo/HAO_0000430
OOL	ocular ocellar line	http://purl.obolibrary.org/obo/HAO_0000662
	orbit	http://purl.obolibrary.org/obo/HAO_0000672
POL	posterior ocellar line	http://purl.obolibrary.org/obo/HAO_0000759
	process	http://purl.obolibrary.org/obo/HAO_0000822
	propodeum	http://purl.obolibrary.org/obo/HAO_0001248
	S1	http://purl.obolibrary.org/obo/HAO_0001997
	S2	http://purl.obolibrary.org/obo/HAO_0001829
	S3	http://purl.obolibrary.org/obo/HAO_0001831
	S4	http://purl.obolibrary.org/obo/HAO_0001832
	S5	http://purl.obolibrary.org/obo/HAO_0001833
	S6	http://purl.obolibrary.org/obo/HAO_0001834
	S7	http://purl.obolibrary.org/obo/HAO_0002185
	sculpture	http://purl.obolibrary.org/obo/HAO_0000913
	sternite	http://purl.obolibrary.org/obo/HAO_0001654
	sulcus	http://purl.obolibrary.org/obo/HAO_0000978
	T1	http://purl.obolibrary.org/obo/HAO_0000053
	T2	http://purl.obolibrary.org/obo/HAO_0000056
	T3	http://purl.obolibrary.org/obo/HAO_0000057
	T4	http://purl.obolibrary.org/obo/HAO_0000058
	T5	http://purl.obolibrary.org/obo/HAO_0000059
	T6	http://purl.obolibrary.org/obo/HAO_0000060
	T7	http://purl.obolibrary.org/obo/HAO_0000061
	tergite	http://purl.obolibrary.org/obo/HAO_0001783
	tibia	http://purl.obolibrary.org/obo/HAO_0001017
	tyloid	http://purl.obolibrary.org/obo/HAO_0001199
	vein	http://purl.obolibrary.org/obo/HAO_0001095
	vertex	http://purl.obolibrary.org/obo/HAO_0001077
	vertical epomial carina	http://purl.obolibrary.org/obo/HAO_0000307
	wing	http://purl.obolibrary.org/obo/HAO_0001089
